# Systematic identification and characterization of repressive domains in *Drosophila* transcription factors

**DOI:** 10.15252/embj.2022112100

**Published:** 2022-12-22

**Authors:** Loni Klaus, Bernardo P de Almeida, Anna Vlasova, Filip Nemčko, Alexander Schleiffer, Katharina Bergauer, Lorena Hofbauer, Martina Rath, Alexander Stark

**Affiliations:** ^1^ Research Institute of Molecular Pathology (IMP) Vienna BioCenter (VBC) Vienna Austria; ^2^ Vienna BioCenter PhD Program Doctoral School of the University of Vienna and Medical University of Vienna Vienna Austria; ^3^ Institute of Molecular Biotechnology (IMBA) Vienna BioCenter (VBC) Vienna Austria; ^4^ Medical University of Vienna Vienna BioCenter (VBC) Vienna Austria

**Keywords:** co‐repressors, high‐throughput screening, repressive domains, short linear motifs, transcription, Chromatin, Transcription & Genomics, Methods & Resources

## Abstract

All multicellular life relies on differential gene expression, determined by regulatory DNA elements and DNA‐binding transcription factors that mediate activation and repression via cofactor recruitment. While activators have been extensively characterized, repressors are less well studied: the identities and properties of their repressive domains (RDs) are typically unknown and the specific co‐repressors (CoRs) they recruit have not been determined. Here, we develop a high‐throughput, next‐generation sequencing‐based screening method, repressive‐domain (RD)‐seq, to systematically identify RDs in complex DNA‐fragment libraries. Screening more than 200,000 fragments covering the coding sequences of all transcription‐related proteins in *Drosophila melanogaster*, we identify 195 RDs in known repressors and in proteins not previously associated with repression. Many RDs contain recurrent short peptide motifs, which are conserved between fly and human and are required for RD function, as demonstrated by motif mutagenesis. Moreover, we show that RDs that contain one of five distinct repressive motifs interact with and depend on different CoRs, such as Groucho, CtBP, Sin3A, or Smrter. These findings advance our understanding of repressors, their sequences, and the functional impact of sequence‐altering mutations and should provide a valuable resource for further studies.

## Graphical abstract

NGS‐based RD‐seq screening method in flies reveals 195 repressive domains in known transcriptional repressors as well as in proteins, not previously linked to gene repression.

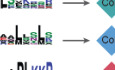

## Introduction

Higher organisms consist of many morphologically different cell types and organs that carry out different functions in the body. Almost all cells possess the same genetic information, yet still only express certain subsets of genes. Hence, a precise regulation of gene expression must take place. The first level of regulation is transcription—the copying of DNA into an RNA transcript by RNA polymerase II. Transcription is regulated by an intricate interplay between regulatory DNA elements, transcription factor (TF) and cofactor proteins, and the RNA polymerase II machinery: TFs bind in a sequence‐specific manner to regulatory DNA and recruit non‐DNA‐binding cofactors, that is, co‐activator or co‐repressor (CoR) proteins, that mediate transcription activating or repressing cues (Reiter *et al*, 2017; Shlyueva *et al*, 2014).

**Figure 1:**
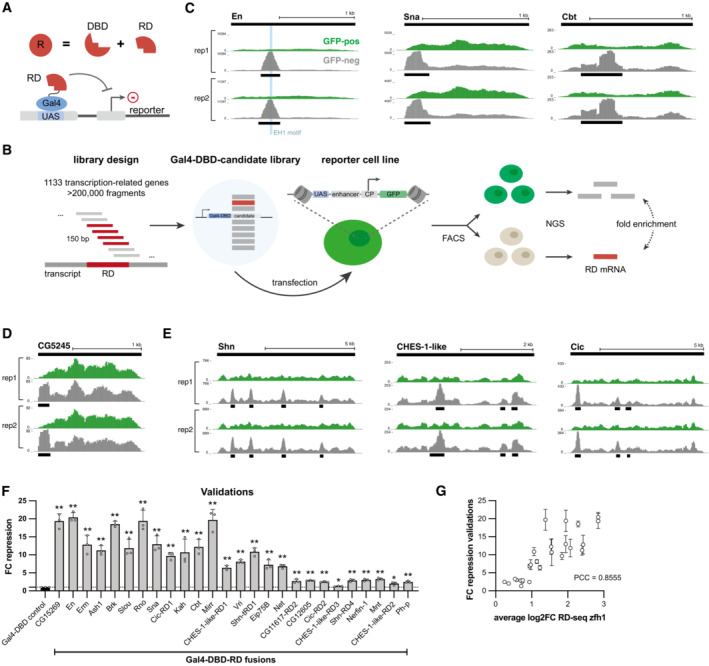
Repressive‐domain‐sequencing (RD‐seq) identifies RDs from a comprehensive pool of candidate fragments ARepressive TFs (R) consist of a DNA‐binding domain (DBD) and a repressive domain (RD), which is sufficient to repress transcription when tethered to a reporter, for example, via the Gal‐UAS system.BSchematic of the RD‐seq pipeline including the candidate library design, the reporter cell line and the RD‐seq workflow (CP: core promoter, FACS: fluorescence‐activated cell sorting, NGS: next‐generation sequencing).C–EUCSC genome browser tracks for two biological replicates of RD‐seq screens with the zfh1‐DSCP reporter cell line. Black bars on the top indicate the entire coding sequence of the respective factor. Shown is the normalized candidate fragment coverage from the fractions of GFP‐negative and GFP‐positive cells and small black bars indicating the detected RD region.FValidations of RD‐seq hits in comparison with the Gal4‐DBD control in the zfh1‐DSCP reporter cell line (mean fold change (FC) repression and individual values of 3 biological replicates, error bars: s.d., two‐tailed paired Student's *t*‐tests comparing to Gal4‐DBD control with * for *P* ≤ 0.05, ** for *P* ≤ 0.01).GComparison between validation FC repression values and average log2 FC in RD‐seq for each RD region in the zfh1‐DSCP reporter cell line (PCC: Pearson correlation coefficient; error bars: s.d. of 3 biological replicates of validations). Source data are available online for this figure.

TFs are modular proteins, consisting of a DNA‐binding domain (DBD) and an effector domain. The effector domain can be an activating domain (AD, also called tAD) or a repressive domain (RD) and can function independently of the full‐length TF (Brent & Ptashne, 1985; Lambert *et al*, 2018; Soto *et al*, 2022). Short RDs of, for example, 31 (Kruppel‐RD; Hanna‐Rose *et al*, 1997) or 55 (Engrailed‐RD; Han & Manley, 1993a) amino acids (AA) can be sufficient to mediate repression when tethered to DNA through a heterologous DBD like the DBD of the yeast transcription factor Gal4 (Gal4‐DBD; Fig 1A). Such tethering assays have allowed the identification of RDs of various repressive TFs such as Engrailed, Snail, Cabut, and others (Han & Manley, 1993b; Fisher *et al*, 1996; Hanna‐Rose *et al*, 1997; Tolkunova *et al*, 1998; Nibu *et al*, 1998b; Belacortu *et al*, 2012; Soto *et al*, 2022).

In addition to the identification of tADs and RDs for individual TFs, pooled screening methods have been developed to systematically identify protein effector domains (reviewed in Soto *et al*, 2022). Examples of such approaches include the identification of tADs within yeast, fly, and human transcription factors or in random peptides (Staller *et al*, 2018; Sanborn *et al*, 2021; Staller *et al*, 2022; Erijman *et al*, 2020; Arnold *et al*, 2018; Alerasool *et al*, 2022; Ravarani *et al*, 2018) or activating and repressing domains among Pfam‐annotated domains (Alerasool *et al*, 2020; Tycko *et al*, 2020). However, no systematic screen for RDs within the TF proteome of any species has been published to date.

The sufficiency of RDs to repress transcription implies that these short domains can specifically interact with and recruit CoRs such as Groucho (Gro), CtBP, and Sin3A (Chinnadurai, 2002; Jennings & Ish‐Horowicz, 2008; Chaubal & Pile, 2018). Interestingly, some known RDs contain short peptide motifs, which are required for RD function and are crucial for the interaction with specific CoRs. For instance, Engrailed and other repressors contain the approximately 10 AA‐long *engrailed homology‐1* (EH1) motif that interacts with the CoR Gro (Logan *et al*, 1992; Smith & Jaynes, 1996; Tolkunova *et al*, 1998). Similarly, the 5 AA short PxDLS motif occurs in the repressive TFs Snail and Knirps and recruits CtBP (Nibu *et al*, 1998a). Yet, how many RDs are explained by these motifs and whether there are other peptide motifs that mediate repression and/or recruit different CoRs remains elusive.

In this study, we established *repressive‐domain‐sequencing* (RD‐seq) to identify short 50 AA‐long RDs across all annotated transcription‐related proteins in *Drosophila melanogaster (Dmel)*. We recovered known and uncovered novel RDs in known repressors and in unannotated proteins. We further identified specific short peptide motifs—conserved from fly to human—and showed that RD function depends on these motifs. In addition, we used immunoprecipitation coupled to mass spectrometry and RNA‐interference (RNAi)‐mediated CoR depletion to link RDs and peptide motifs to specific CoRs, revealing RD‐CoR interactions and functional dependencies.

Our work provides a resource for *Drosophila* RDs as well as, the first step in building a systematic dictionary for repressors, their RDs and interacting CoRs—a valuable tool to comprehend the diverse mechanisms of transcriptional repression.

## Results

### RD‐seq identifies RDs of known and novel transcriptional repressors

To systematically identify RDs, we established RD‐seq, a next‐generation sequencing (NGS)‐based approach to identify RDs from a comprehensive pool of candidate fragments (Fig 1B). For this purpose, we adapted the tAD‐seq protocol (Arnold *et al*, 2018) and combined it with a synthetic candidate library and reporter cell lines that constitutively express GFP.

We generated a Gal4‐DBD‐fused candidate library consisting of over 200,000 150‐bp‐long DNA fragments coding for 50 AA. The candidates were designed to cover the protein‐coding open‐reading frames of 1,133 transcription‐related *Dmel* genes in a tiled fashion with steps of 6–15 bp, corresponding to 2–5 AA (Fig 1B; Dataset EV1, see library design in Material and Methods). Using CRISPR/Cas9, we created a *Dmel* S2 cell line with an integrated GFP‐expressing reporter‐gene cassette containing UAS sites to allow Gal4‐DBD‐mediated tethering of the candidates. Three days after transfection of the reporter cell line with the candidate library, we separated cells into GFP‐positive and GFP‐negative cells via fluorescent‐activated cell sorting (FACS), followed by NGS‐based quantification of the candidate mRNAs in GFP‐positive and GFP‐negative cells. Since GFP‐negative cells should contain candidates that repress transcription, we determined the enrichment of candidates in GFP‐negative over GFP‐positive cells, called RDs by their significant enrichment (*P* ≤ 1 × 10^−5^; FC ≥ 1.5; Fig 1B), and for subsequent analyses only considered RDs that were detected in two of two replicates (e.g., Fig 1C–E, see Material and Methods).

To capture different RDs, we performed RD‐seq screens with two different reporter cell lines in which GFP expression was driven by distinct enhancer‐promoter pairs, namely zfh1‐DSCP and ent1‐rps12 (Dataset EV2). We performed two replicates per cell line and collectively, the screens in the two cell lines resulted in a total of 195 unique RDs in 175 proteins (Dataset EV3). 114 of the RD‐seq hits (58%) are within known or putative repressors (references in Dataset EV3), including the known RDs in the well‐characterized repressive TFs Engrailed (En), Snail (Sna), and Cabut (Cbt; Tolkunova *et al*, 1998; Nibu *et al*, 1998a; Belacortu *et al*, 2012; Fig 1C). In the case of En, the peak summit of candidate enrichment coincided with the EH1 motif, known to be essential for the repressive activity (Tolkunova *et al*, 1998; Fig 1C blue bar in left panel). 79 RDs are in known or putative repressive TFs for which no RD had been mapped before (references in Dataset EV3). Moreover, we also found 81 RDs (42% of hits) in proteins that have not been implicated in repression so far, for example, RDs within 18 previously uncharacterized *Dmel* proteins such as the putative Zn‐finger TF CG5245 (Fig 1D). Interestingly, some proteins have multiple RDs, for example, Schnurri (Shn), for which several repressive regions have been described before (Cai & Laughon, 2009), but also CHES‐1‐like and Capicua (Cic) for which we identify three RDs each (Fig 1E). Overall, RD‐seq characterizes known as well as novel repressor proteins and maps RDs for both (Dataset EV3).

**Figure EV1:**
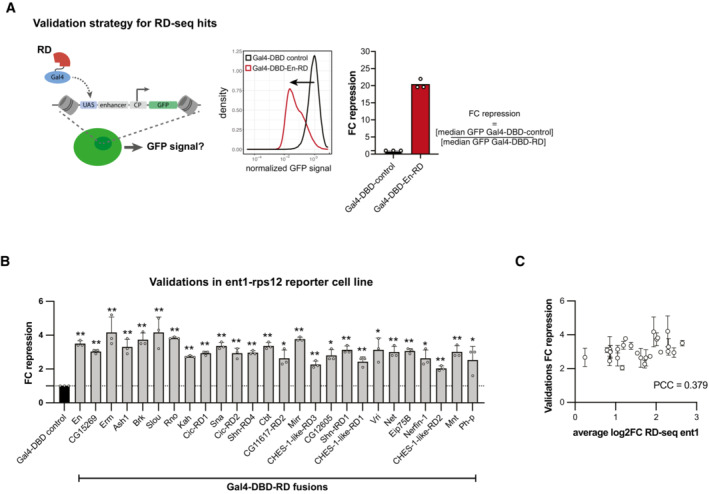
Validations of RD‐seq hits Validation strategy for RD‐seq hits. The reporter cell line is transfected with either the Gal4‐DBD‐fused RD or a Gal4‐DBD construct as a control, followed by assessment of the GFP signal by flow cytometry (left). The density distribution shows the normalized GFP signal of cells expressing either the Gal4‐DBD control or the Gal4‐DBD‐En‐RD construct (middle). The fold change (FC) repression is calculated as the ratio of the median GFP intensity of the Gal4‐DBD control and the Gal4‐DBD‐RD condition (mean FC repression and individual values of 3 biological replicates; right).Validations of RD‐seq hits in comparison with the Gal4‐DBD control in the ent1‐rps12 reporter cell line (mean FC repression and individual values of 3 biological replicates, error bars: s.d., two‐tailed, paired Student's *t*‐tests comparing to Gal4‐DBD control with * for *P* ≤ 0.05, ** for *P* ≤ 0.01).Comparison between validation FC repression values and average log2 FC in RD‐seq for each RD region in the ent1‐rps12 reporter cell line (PCC: Pearson correlation coefficient; error bars: s.d. of 3 biological replicates of validations). Source data are available online for this figure.

To validate RD‐seq hits and assess the method's specificity, we selected 26 of the 83 RDs that were detected in both reporter cell lines, including both strong and weak RDs from rank 1 to rank 82. We cloned a 150‐bp (50 AA) fragment per RD (Dataset EV4), individually recruited the 26 RDs to the integrated zfh1‐DSCP GFP reporter via the Gal4‐DBD and assessed changes in GFP expression through flow cytometry in comparison with a control condition (Gal4‐DBD alone; three independent replicates per RD and control). As a measure of the repressive strength of the RD, we calculated the fold change (FC) repression as the median GFP signal of cells with the Gal4‐DBD control versus cells with the Gal4‐DBD‐RD (Fig EV1A). In the zfh1‐DSCP reporter cell line, this validated all 26 hits (Student's *t*‐test *P* ≤ 0.05; FC > 1; Fig 1F; Dataset EV4) and their repressive strengths in the validation experiments correlated well with the RD‐seq enrichments (Pearson correlation coefficient (PCC) = 0.86, Fig 1G). Similarly, all 26 hits were validated in the ent1‐rps12 reporter cell line (Fig EV1B; Dataset EV4), yet the dynamic range was narrower, compressing the quantitative agreement to PCC = 0.38 (Fig EV1C). We therefore chose to use the zfh1‐DSCP reporter cell line for all subsequent analyses. Overall, these results validate RD‐seq as a high‐throughput method to identify RDs and assess their repressive strength quantitatively.

### RDs overlap both, IDRs and DBDs, and show a preference toward N‐terminal positions within a TF

**Figure 2:**
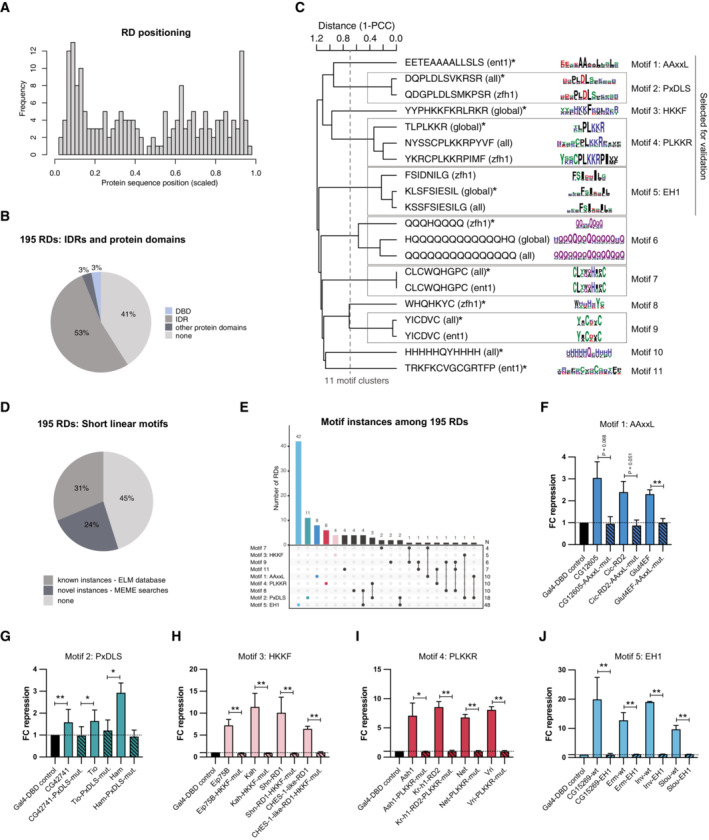
Characterization of RDs and RD dependency on short linear motifs AFrequency histogram of the position of the center of the 50 AA RD within its full‐length protein for all 195 RDs. Positions are scaled over the length of the respective protein sequences.BPie chart showing the overlap between RDs and intrinsically disordered regions (IDRs) according to the MobiDB‐lite database, DNA‐binding domains (DBDs), and other annotated protein domains from the Pfam or ProSitePatterns databases.CHierarchical clustering of MEME *de novo* motif discovery motif hits with distinct subsets of RDs (all, global, zfh1, and ent1). The tree was cut at height 0.7, resulting in 11 nonredundant distinct motifs (* motif versions used for FIMO searches).DPie chart showing ELM database CoR‐interacting motifs and MEME motif instances among the 195 RDs.ENumber of instances of 9 MEME motifs (excluding motif 6 and 10) among the 195 RDs and co‐occurrence of these motifs (N: total number of RDs with that motif).F–JValidation results for wild‐type and mutated RDs in the zfh1‐DSCP reporter cell line with the following conserved motifs: (F) Motif 1—AAxxL, (G) Motif 2—PxDLS, (H) Motif 3—HKKF, (I) Motif 4—PLKKR, (J) Motif 5—EH1 (mean FC repression values of 3 biological replicates, error bars: s.d., two‐tailed paired Student's *t*‐tests comparing RD wt vs. mutant with * for *P* ≤ 0.05, ** for *P* ≤ 0.01, or exact *P*‐value when not significant). Source data are available online for this figure.

**Figure EV2:**
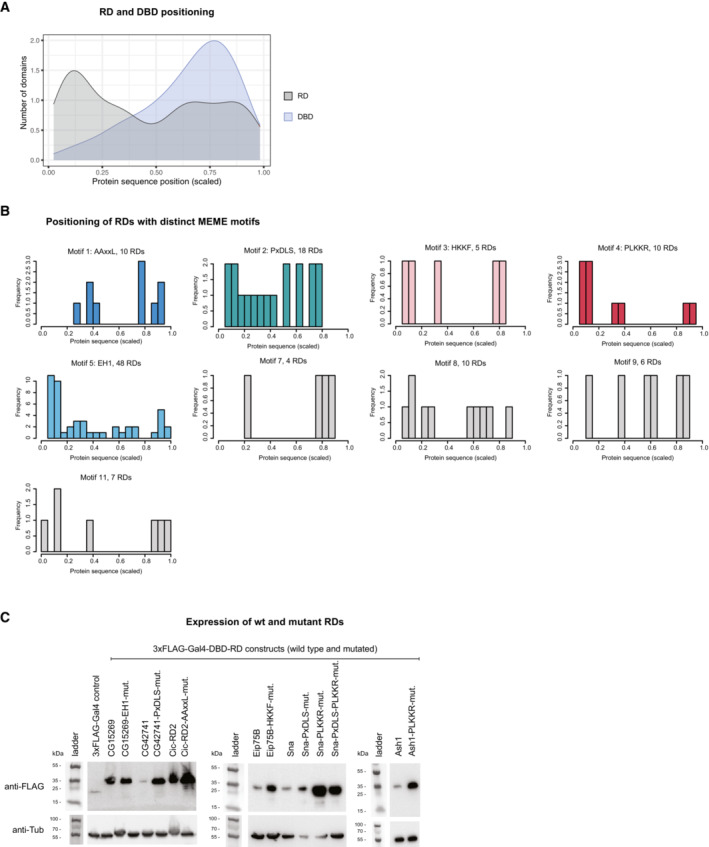
RD and DBD positioning and expression of mutated RDs Positioning of RDs and DBDs. Density distribution of the position of the center of the 50 AA RD or the DBD regions within their full‐length protein. Positions are scaled over the length of the respective protein sequences.Positioning of RDs with distinct motifs from MEME *de novo* motif searches. Shown are frequency histograms of the position of the center of the 50 AA RD within its full‐length protein for all RDs containing each motif type. Positions are scaled over the length of the respective protein sequences.Western blots for FLAG‐Gal4‐DBD‐tagged wild‐type and motif mutant RDs expressed in the zfh1‐DSCP reporter cell line (anti‐Tubulin as loading control). Source data are available online for this figure.

Having identified and validated many RDs, we next wondered where within the TFs' protein sequences they typically occur and analyzed the 195 RDs' positions relative to the proteins' N‐ and C‐termini. As illustrated by the RDs in CG5245, Shn, CHES‐1‐like and Cic above (Fig 1D and E), RDs occur at different positions within the full‐length proteins. Interestingly, however, they occur more frequently toward the N‐termini of TFs compared with the C‐termini or more intermediate positions (Fig 2A). While the functional significance of the N‐terminal positions remains unclear, the TFs' DBDs show the opposite trend with a preference toward the C‐termini of the proteins (Fig EV2A). These opposing trends suggest that RDs and DBDs are typically separate and nonoverlapping. Indeed, only 3% of RDs overlap with DBDs (Fig 2B), for example, the RD of the repressor Hang. In addition, 3% of RDs overlap with other annotated protein domains (Pfam and ProSitePatterns databases, for example, the Parp catalytic domain in Parp‐RD), 53% overlap with intrinsically disordered regions (IDRs; MobiDB‐lite database), while 41% fall into unannotated regions (Fig 2B; Dataset EV5). The large overlap of RDs and IDRs suggests an important role of these regions for repressive TFs, particularly because only 36% of all *Dmel* transcription‐related proteins and 28% of all *Dmel* proteins overlap with IDRs. The relevance of IDRs in repressors could be similar to the one reported for activating TFs (Boija *et al*, 2018; Chong *et al*, 2018; Sabari *et al*, 2018; Basu *et al*, 2020; Brodsky *et al*, 2020). Still, many RDs do not overlap with any known protein domains or other annotated protein features, emphasizing the need for better characterization of RDs and the protein sequence contexts in which they can function.

### RDs contain recurring short linear peptide motifs

As RDs can contain short peptide motifs that mediate repressor‐CoR interactions (Tolkunova *et al*, 1998; Nibu *et al*, 1998b), we sought to identify recurrent peptide motifs that could explain the RDs' repressive functions. We performed *de novo* motif discovery using MEME (Bailey *et al*, 2015) for all 195 RD‐seq hits and subsets (see Material and Methods), followed by clustering of similar motifs to obtain 11 distinct short peptide motifs (Fig 2C; Dataset EV6).

Among these, we found previously annotated short linear motifs (SLiMs) known to be important for repression and interaction with CoRs, such as AAxxL, PxDLS, and EH1 (Dataset EV7). The AAxxL motif resembles the Sin3A‐interacting domain (SID), which recruits the CoR Sin3A (Ayer *et al*, 1995, 1996; Kasten *et al*, 1996; Belacortu *et al*, 2012). The SID in the *Dmel* protein Cabut, for example, is FKMNRKRAAEVALPPVCT containing an AAXXL‐like motif (Belacortu *et al*, 2012). The PxDLS motif, for instance, PMDLS in Knirps is known to facilitate the recruitment of CtBP (Nibu *et al*, 1998a; Quinlan *et al*, 2006), while the EH1 (engrailed homology‐1) motif, for example, LAFSISNILSD in Engrailed is known to mediate the interaction with the CoR Groucho (Gro; Tolkunova *et al*, 1998; Copley, 2005). Motif 8 resembles the HCF‐1‐binding motif, which mediates interaction with the host cell factor‐1 (Hcf in *Dmel*) that has been implicated in both transcriptional activation and repression (Wysocka *et al*, 2003; Zargar & Tyagi, 2012).

In addition, motifs 7, 9, and 11 resemble zinc finger domains from the Pfam or ProSitePatterns databases, a domain type known to mediate DNA‐binding, protein–protein interactions (reviewed in Brayer & Segal, 2008), but also transcriptional repression (Tapia‐Ramírez *et al*, 1997; Lee *et al*, 2005). Two additional motifs were of low sequence complexity with multiple glutamine (motif 6) or histidine residues (motif 10), which have been observed in activating and repressing TFs (Salichs *et al*, 2009; Atanesyan *et al*, 2012; Ramazzotti *et al*, 2012). Hence, to avoid studying compositional biases of transcriptional regulators in general, we excluded the Q and H repeat motifs from further analysis and instead focused on the other 9 MEME motifs.

We also found two novel, previously unannotated motifs, motifs 3 and 4, that we termed PLKKR and HKKF, respectively. The two motifs are potentially novel SLiMs and both are positively charged, consistent with the positive charges in recently identified repressive domains (Tycko *et al*, 2020).

We next mapped the positions of all instances of the 9 main motifs within the 195 RDs (Dataset EV6), as well as instances of CoR‐interacting motifs from the ELM database (Dataset EV8). Of all 195 RDs, 55% contain at least one instance of these motif types, of which 24% could only be identified with the *de novo* defined motifs (Fig 2D). For the AAxxL motif, we find multiple novel instances, for example, in the RDs of Glut4EF, CG12605 and Cic (Dataset EV6). Interestingly, EH1 was the most abundant motif, present in 48 different RDs (Fig 2E). This large group of EH1 motif‐containing RDs is the main driver of the positional bias of RDs toward the N‐termini of the full‐length TFs (Figs 2A and EV2B). Moreover, some RDs contain combinations of peptide motifs, such as the RDs of Sna, Esg, and Wor that all contain both, the PxDLS and the PLKKR motif (Fig 2E).

### Short peptide motifs are required for RD function

Next, we assessed the necessity of five of the known and novel peptide motifs for the repressive activity of RDs by mutating the motifs to Alanine residues. We selected motif types 1 through 5 (i.e., AAxxL, PxDLS, HKKF, PLKKR, and EH1) and mutated between three and four different RDs per motif type (Dataset EV4 with all AA sequences). We first confirmed that the mutated RDs were still expressed at least to equal levels compared with the wild‐type RDs, such that changes in the repressive activity are not caused by impaired protein stability (Fig EV2C). Interestingly, many of the mutated RDs are present at even higher levels than the wild‐type RDs, suggesting that repressive function of RDs might be coupled to protein destabilization as has been reported for trans‐activating domains (Salghetti *et al*, 2000; Geng *et al*, 2012). For all instances of all motif types, we observed a loss of repressive activity upon motif mutation, rendering the mutated RD variants as ineffective as a Gal4‐DBD control (Fig 2F–J; Dataset EV4). For two AAxxL motif‐containing RDs, the loss of repression was not significant but still noticeable (Fig 2F). The results so far reveal known as well as novel short peptide motifs and show that RDs rely on such motifs to repress transcription.

### RDs with different peptide motifs bind distinct CoRs

Some of the recurrent peptide motifs that are essential for RD function have been described previously to facilitate the interaction between repressors and CoRs (Ayer *et al*, 1996; Tolkunova *et al*, 1998; Nibu *et al*, 1998a). After validating the motifs' necessity for RD function, we wanted to explore their mechanism of action, specifically the CoR proteins they might recruit. To determine the interactors of RDs with different motifs, we performed immunoprecipitations of RDs containing PxDLS, AAxxL, PLKKR, and HKKF motifs followed by quantitative mass spectrometry (IP‐MS). We excluded EH1 motif‐containing RDs, since this motif and its interaction with Gro have been studied extensively (Logan *et al*, 1992; Tolkunova *et al*, 1998; Copley, 2005; Jennings *et al*, 2006). IPs were performed using an anti‐FLAG antibody and nuclear lysate of *Dmel* S2 cells overexpressing 3xFLAG‐Gal4‐DBD‐tagged RDs with a specific peptide motif or 3xFLAG‐Gal4‐DBD as negative control. To ensure that each motif is in the sequence context of a functional RD, while also ensuring that binding partners of the motif rather than any individual RDs are characterized, we performed immunoprecipitation experiments with pools of four RDs that share the motif of interest and contained none of the other motifs at stringent thresholds. Some baits contained subthreshold motifs, which however differed substantially from the consensus (see Dataset EV9), and the pooling should dilute their possible contributions. In addition, mutations of the primary motifs within the baits rendered all of the tested ones inactive (Fig 2F–I), making it unlikely that other low‐scoring motifs contribute to the repressive activity or the IP‐MS results.

**Figure 3:**
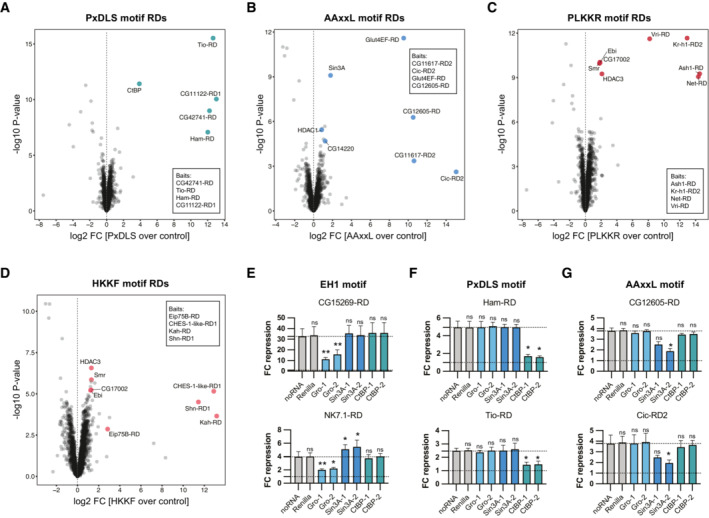
RD–CoR interactions and dependencies A–DVolcano plots showing results of immunoprecipitations followed by mass spectrometry (IP‐MS; 3 biological replicates) for pools of RDs with specific repressive motifs: (A) PxDLS, (B) AAxxL, (C) PLKKR, (D) HKKF. Highlighted are FLAG‐Gal4‐DBD‐tagged RDs used as baits (specified in insets) and their CoR interactors. Strongly de‐enriched proteins are unrelated to transcriptional regulation (e.g., the lysosomal protein CP1 Cysteine proteinase‐1).E–GValidations of RDs upon RNAi‐mediated depletion of CoRs in the zfh1‐DSCP reporter cell line (mean FC repression values of 3 biological replicates, error bars: s.d., two‐tailed paired Student's *t*‐tests comparing to noRNA control with * for *P* ≤ 0.05, ** for *P* ≤ 0.01, or ns for *P* > 0.05). The targeted CoR is shown on the x‐axis (2 different dsRNA constructs per CoR). The repressive motifs contained in the tested RDs are indicated above the panels. Source data are available online for this figure.

As expected, RDs containing the PxDLS motif enriched for the CoR CtBP (Nibu *et al*, 1998a; Fig 3A) and AAxxL motif‐containing RDs enriched for the Sin3A CoR complex members Sin3A, HDAC1, and CG14220 (Ayer *et al*, 1996; Brubaker *et al*, 2000; Belacortu *et al*, 2012; Fig 3B). RDs with the PLKKR motif interacted with four subunits of the Smrter CoR complex (orthologous to human NCoR/SMRT), namely Smr, CG17002, Ebi, and HDAC3 (Fig 3C). While the PLKKR motif has not been described as a CoR‐interacting SLiM, our IP‐MS results are consistent with two studies describing the interaction of *Dmel* Snail with the Smrter subunit Ebi through a YxxCPLKKRP sequence (Qi *et al*, 2008) and the human MeCP2 protein with the Ebi ortholog TBLR1 through an extended domain that contains a PIKKR sequence (Lyst *et al*, 2013; Kruusvee *et al*, 2017). Our data suggest that the PLKKR motif is a recurrent SLiM utilized by various repressive TFs, which likely mediate transcriptional repression through the Smrter CoR complex.

Interestingly, RDs with the HKKF motif also enriched for the Smrter complex (Fig 3D), consistent with a report that the repressive TF Shn interacted with Smrter via a NISRYLHKKFKRLASTTEVDS sequence (Cai & Laughon, 2009). This sequence not only contains an HKKF motif but also coincides with the first of four RDs we find within Shn (Figs 1E and 2H). Hence, similar to PLKKR, the HKKF motif is a SLiM likely utilized by various repressors to interact with the Smrter complex.

### RDs with distinct peptide motifs depend on different CoRs

**Figure EV3:**
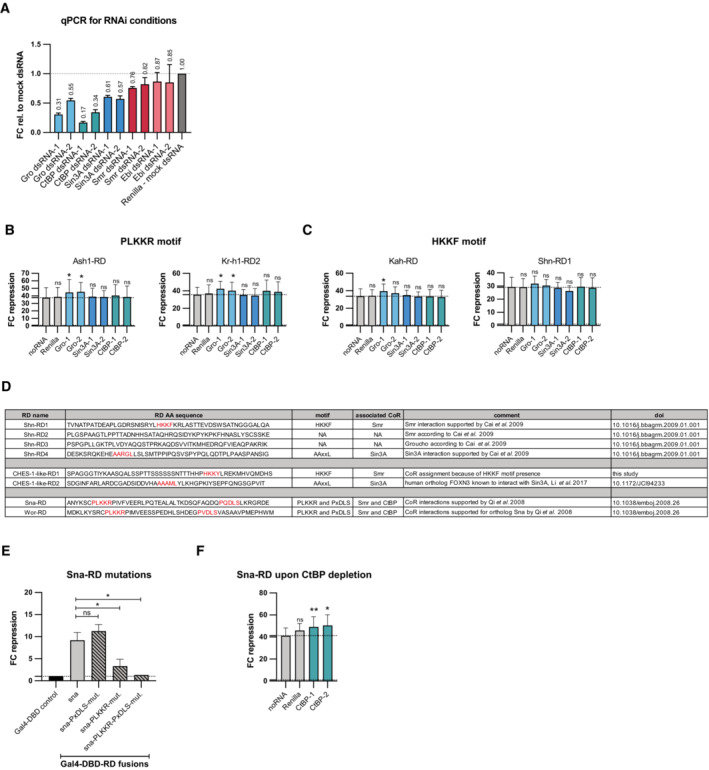
RNAi‐mediated co‐repressor depletion and repressors with multiple RDs and repressive motifs AAssessment of depletion of CoR mRNA with RNAi through reverse transcription quantitative PCR (RT–qPCR). Each CoR was targeted with 2 different dsRNA constructs (x‐axis). A dsRNA targeting Renilla was used as a negative control. Shown is the fold change (FC) relative to the control condition calculated with the Delta–Delta Ct Method (3 technical replicates, error bars: s.d.).B, CValidations of RDs upon RNAi‐mediated depletion of CoRs in the zfh1‐DSCP reporter cell line (mean FC repression values of 3 biological replicates, error bars: s.d., two‐tailed, paired Student's *t*‐tests comparing to noRNA control with * for *P* ≤ 0.05, ** for *P* ≤ 0.01, or ns for *P* > 0.05). Each CoR was targeted for depletion with 2 different dsRNA constructs (x‐axis). The repressive motif contained in the tested RD is indicated above the panels.DExamples of repressors with multiple RDs and RDs with multiple repressive motifs. Shown are RD sequences, presence of repressive motifs, their associated interacting CoRs and literature references.EValidation of wild‐type and mutant Sna‐RD in the zfh1‐DSCP reporter cell line (mean FC repression of 3 biological replicates, error bars: s.d., two‐tailed, paired Student's *t*‐tests comparing RD wt vs. mutant with * for *P* ≤ 0.05 or ns for *P* > 0.05).FValidation of Sna‐RD upon RNAi‐mediated depletion of CtBP in the zfh1‐DSCP reporter cell line (mean FC repression values of 3 biological replicates, error bars: s.d., two‐tailed paired Student's *t*‐tests comparing to noRNA control with * for *P* ≤ 0.05, ** for *P* ≤ 0.01, or ns for *P* > 0.05). CtBP was targeted for depletion with 2 different dsRNA constructs (x‐axis). Source data are available online for this figure.

We set out to corroborate the results of the IP‐MS experiments by assessing CoR requirements for RD function. We designed dsRNAs for the RNAi‐mediated depletion of four different CoRs by dsRNA transfection in *Drosophila* S2 cells. RT–qPCRs showed the successful depletion of Gro, CtBP, and Sin3A mRNAs through treatment with two distinct dsRNAs each (Fig EV3A). However, we could not sufficiently strongly deplete the transcripts of Smr or Ebi despite the use of two different dsRNA constructs each and therefore could not follow up on the dependency of RDs on the Smrter complex.

RNAi‐mediated CoR depletion revealed that EH1 motif‐containing RDs specifically depended on Gro but not on CtBP or Sin3A (Fig 3E). The depletion of Sin3A resulted in an increase in repressive activity for the NK7.1‐RD, which might be due to secondary effects of the loss of this CoR. PxDLS motif‐containing RDs depended on CtBP but not Gro or Sin3A (Fig 3F) and AAxxL motif‐containing RDs required Sin3A but not the other two CoRs (Fig 3G). Each of these dependencies was consistent with literature reports (Kasten *et al*, 1996; Nibu *et al*, 1998a; Jennings *et al*, 2006) or the IP‐MS results for the different motifs (Fig 3A and B). Interestingly, RDs with PLKKR or HKKF motifs maintained their repressive function in the absence of each of these 3 CoRs (Fig EV3B and C), which indicates that these motifs are independent of Gro, CtBP, and Sin3A, in line with their likely dependence on the Smrter CoR complex (Fig 3C and D). The RDs of Ash1, Kr‐h1, and Kah showed a slight increase in repression upon the loss of Gro, which might result from secondary effects of Gro depletion (Fig EV3B and C).

Overall, our experiments suggest that repressors mediate repression through short, conserved peptide motifs, which are required for the interaction with certain CoRs. Interestingly, some repressors contain multiple RDs that recruit different types of CoRs, for example, Schnurri with RDs for Gro, Sin3A, and Smrter, and CHES‐1‐like with RDs for Smrter and Sin3A (Figs 1E and EV3D). There are also cases in which RDs contain two distinct peptide motifs, such as PLKKR and PxDLS within the RD of Snail (Figs 2E and EV3D). Investigating different Snail‐RD mutants showed that both motifs contribute to the RD's repressive activity: Mutating the PxDLS motif alone did not impair RD function, and while mutating the PLKKR motif deceased RD function, only the simultaneous mutation of both motifs abolished it (Fig EV3E). Consistently, the RD of Sna remained functional when CtBP was depleted by RNAi and even showed slightly higher repressive activity (Fig EV3F), presumably because it was still able to recruit the Smrter complex via its PLKKR motif. These observations of proteins with multiple RDs and likely different interacting CoRs have interesting implications for how even single transcriptional repressors could act in different ways to achieve gene silencing.

### Fly RD motifs are conserved across species and predict human repressors

Some of the RD motifs and their interactions with CoRs are known to be conserved across species as distant as flies and mammals. This includes the EH1, PxDLS, and AAxxL motifs and their interaction with the human orthologs of Gro, CtBP, and Sin3A, which have been described for individual human proteins (Logan *et al*, 1992; Cook *et al*, 1999; Quinlan *et al*, 2006; Spittau *et al*, 2007; Belacortu *et al*, 2012).

**Figure 4:**
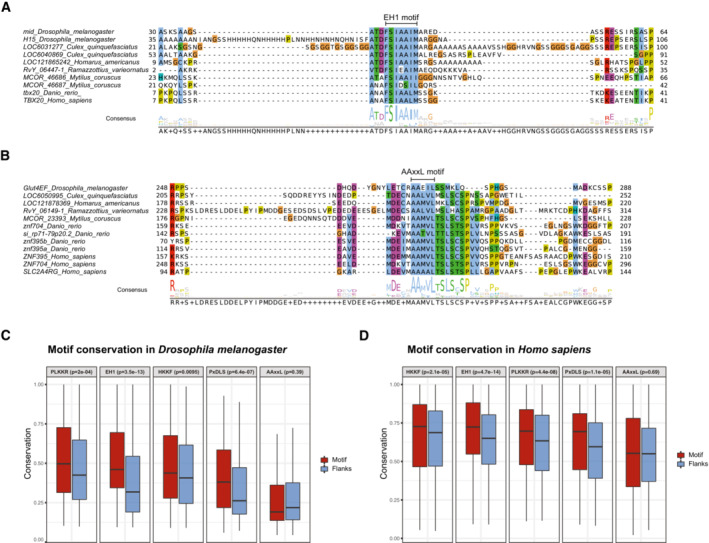
RDs and repressive peptide motifs are conserved across species A, BSequence alignment for a region of *Dmel* TFs (A) Mid and H15 containing the EH1 motif or (B) Glut4EF containing the AAxxL motif and the respective orthologous sequences from different species. Numbers on the left and right indicate the range of amino acids shown referring to the full‐length proteins. Consensus sequences are indicated at the bottom.C, DConservation of repressive motifs over their flanking regions for motif instances from all (C) *Dmel* and (D) human transcription‐related genes. Shown are box plots with average conservation scores of motif instances (red) and respective flanking regions (blue) for each motif type. N of instances for fly: 166 AAxxL, 127 EH1, 112 HKKF, 89 PLKKR, 117 PxDLS. N of instances for human: 261 AAxxL, 235 EH1, 256 HKKF, 208 PLKKR, 200 PxDLS. The box plots mark the median, upper and lower quartiles and 1.5× interquartile range (whiskers). Motif types are indicated on the top together with FDR‐corrected *P*‐values from two‐sided, paired Wilcoxon signed rank tests comparing the conservation of motif instances and their flanking regions.

**Figure EV4:**
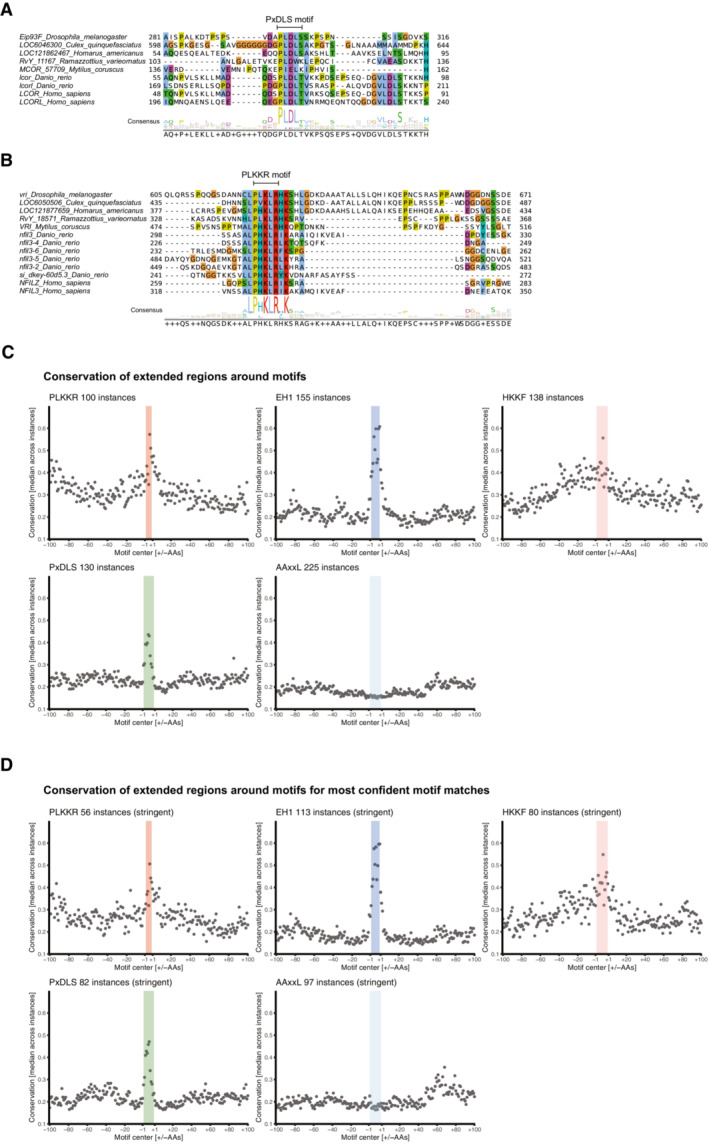
Sequence alignments of RDs and conservation of regions around repressive motifs A, BSequence alignments for a region of *Dmel* TFs (A) Eip93F containing the PxDLS motif and (B) Vri containing the PLKKR motif and the respective orthologous sequences from different species. Numbers on the left and right indicate the range of amino acids shown referring to the full‐length proteins. Consensus sequences are indicated at the bottom.C, DMetaplots showing the median conservation scores of positions within repressive motifs (highlighted in color) and ± 100 flanking amino acids for motif instances among fly transcription‐related proteins resulting from standard (C) and stringent (D) FIMO searches. See also Fig 4C and D for summary analyses.

To illustrate the deep conservation of individual instances of these motifs, we created sequence alignments for repressive TFs from *Dmel* containing repressive peptide motifs and the TFs' orthologs in different species over a wide phylogenetic range (Figs 4A and B, and EV4A and B). The alignment of Mid and its orthologs illustrates the conservation of the EH1 motif from insects to mammals (Fig 4A), as does the alignment of Eip93F and its orthologs for the PxDLS motif (Fig EV4A). The alignment of Glut4EF containing the AAxxL motif shows the strong conservation not only of the core AAxxL motif but also of the flanking sequences, suggesting that this motif might in fact be longer (Fig 4B). Lastly, also the PLKKR motif within Vri is strongly conserved in Vri's orthologs (Fig EV4B).

If instances of the repressive motifs EH1, AAxxL, PxDLS, PLKKR, and HKKF were indeed functional in fly and human TFs, they would on average be more highly conserved than expected in closely related insect or vertebrate species, respectively. This reasoning has previously been applied to short microRNA‐binding‐site sequences in flies and mammals (Brennecke *et al*, 2005; Lewis *et al*, 2005) or TF‐binding sites (e.g., Stark *et al*, 2007) and benefits from the better alignability of sequences between closely related species rather than distal ones. Following this reasoning, we created multiple protein sequence alignments of *Dmel* and human transcription‐related proteins within insect and vertebrate orthogroups, respectively. We next calculated conservation scores for each AA position of these transcription‐related proteins from *Dmel* (the same proteins as covered in the RD‐seq library, Dataset EV1) and human (based on Vaquerizas *et al*, 2009; Lambert *et al*, 2018; Dataset EV10) and assessed the conservation of the 5 different peptide motifs (Fig 4C and D) compared with immediately flanking sequences. In both insects and vertebrates, we observed significantly higher conservation of the EH1, PLKKR, PxDLS and HKKF motifs in comparison with their flanking regions (Fig EV4C and D). For HKKF motifs and—to a lesser extent PLKKR motifs—sequence conservation extends beyond the core motifs, albeit in a broad pattern that likely results from overall more highly conserved protein regions or lower sequence complexity rather than longer motifs.

Overall, the conservation of the RD motifs compared with the flanking sequences (Fig 4C and D) suggests that at least EH1, PxDLS, PLKKR, and HKKF are under purifying selection in both insects and vertebrates and thus likely functionally relevant. The AAxxL motif was not significantly more highly conserved than its flanks for both, insects, and vertebrates (Fig 4C and D). While this might be due to the motif being longer and extending into the flanks, as exemplified by Glut4EF (Fig 4B), this does not seem to be the case in general: across all motif instances, both the motif and the flanking regions show only background‐level conservation below the conservation seen for the other motifs (Fig EV4C), even when only considering highly stringent motif matches (Fig EV4D). Indeed, while some Sin3A‐interacting peptides found in the literature match the AAxxL motif, others only resemble AAxxL but do not strictly follow the pattern (Ayer *et al*, 1996; Cook *et al*, 1999; Brubaker *et al*, 2000; Belacortu *et al*, 2012; Chandru *et al*, 2018). For example, the Sin3A‐interacting domain of the *Dmel* repressor Cabut contains an AAEVAL core, which does not strictly follow the AAxxL pattern (Belacortu *et al*, 2012). Similarly, the Sin3A‐interacting domains of the human TIEG2 and TET1 contain EAVEAL and AIEAL sequences, respectively (Cook *et al*, 1999; Chandru *et al*, 2018). Therefore, even though the AAxxL motif validated experimentally and AAxxL motif‐containing RDs interacted with and depended on Sin3A (Figs 2F and 3B and G), its function is not reflected by increased conservation, presumably because the Sin3A‐interacting motif is more flexible.

Among the human transcription‐related proteins that contained repressor motifs (Datasets EV10 and EV11) were indeed many known repressors: For example, among the 30 highest scoring PxDLS motif matches, we found 19 proteins known to repress transcription through CtBP, for example, MECOM (also EVI1), ZFPM1, and PRDM16 (Izutsu *et al*, 2001; Katz *et al*, 2002; Kajimura *et al*, 2008; additional references in Dataset EV11). The highest scoring PLKKR matches include MeCP2, known to contain a PIKKR sequence and to interact with NCoR/SMRT (Kruusvee *et al*, 2017), and other proteins that have been implicated in repression but not been associated with any CoR, such as NSD2 and ASH1L (Nimura *et al*, 2009; Tanaka *et al*, 2011; Dataset EV11). Similar to the situation in *Dmel* (see Fig EV3D), some human proteins like BCL3 contain both the PxDLS and PLKKR motifs, suggesting that they recruit both, the CtBP and the NCoR/SMRT CoR complexes.

These analyses not only highlight the deep evolutionary conservation of repressive peptide motifs but also provide both, an annotation of human repressive TFs that contain such motifs and a resource to study human TF sequences and assess the potential functional impact of mutations in these proteins.

## Discussion

Transcriptional activation and repression are both crucial for gene regulatory programs in different cell types and under changing environmental conditions. Yet, while transcriptional activators and trans‐activating domains (tADs) have been studied extensively (Arnold *et al*, 2018; Ravarani *et al*, 2018; Staller *et al*, 2018, 2022; Erijman *et al*, 2020; Sanborn *et al*, 2021; Alerasool *et al*, 2022), our knowledge on transcriptional repressors, their RDs, and interacting CoRs remained limited. Here, we developed the high‐throughput assay RD‐seq, to systematically map RDs throughout the sequences of all transcription‐related proteins in *Dmel* (Fig 1). This identified 195 unique RDs in known repressors and proteins that have not been implicated in repression, providing the first comprehensive screen for RDs and a resource for RD–CoR associations.

We find that RDs contain short recurring peptide motifs required for the RDs' repressive functions (Fig 2), and these motifs recruit specific CoRs as demonstrated by IP‐MS and functional RD‐CoR dependencies (Fig 3). These include known examples, such as the well‐established EH1‐Gro and PxDLS‐CtBP interactions (Tolkunova *et al*, 1998; Nibu *et al*, 1998b; Ryu & Arnosti, 2003; Jennings *et al*, 2006) and the less well‐studied interaction of AAxxL and Sin3A (Ayer *et al*, 1996; Zhang *et al*, 2001; Belacortu *et al*, 2012). Furthermore, our study reveals two new recurrent SLiMs, PLKKR and HKKF, found in RDs that bind the Smrter CoR complex (Figs 2 and 3). This finding is consistent with two studies reporting the interaction between extended fly or human protein domains with the Smrter or NCoR/SMRT complex, respectively (Qi *et al*, 2008; Cai & Laughon, 2009; Kruusvee *et al*, 2017). Our results refine these studies to pinpoint PLKKR‐ and HKKF‐like motifs in these domains. Indeed, point mutations within MeCP2 that lead to the Rett syndrome (Lyst *et al*, 2013; Kruusvee *et al*, 2017) map to the PIKKR motif, highlighting the importance and potential disease association of RDs.

The lack of systematic annotations of RDs in fly TFs makes it difficult to evaluate the specificity and sensitivity of RD‐seq against an independent benchmark dataset. However, the candidate library contained fragments covering 438 TFs whose regulatory activity was assessed in a previous study (Stampfel *et al*, 2015). We found RDs in 79 of these TFs, of which 50 (63%) are repressors, which increases to 61 (77%) for TFs that are at least weakly repressive and 73 (92%) for TFs that are not activators (see Material and Methods). In addition, we recover a variety of RDs that have been mapped in studies on individual repressive TFs (Tolkunova *et al*, 1998; Hemavathy *et al*, 2004; Cai & Laughon, 2009; more references in Dataset EV3). These results suggest that RD‐seq is highly specific, consistent with the validation rate of 26 out of 26 RDs (Fig 1F). Of the 156 repressive TFs derived from Stampfel *et al* (2015), we found RDs for 50 (32%), and for the 43 strongly repressive TFs, we found RDs in 22 (51%). The recovery of RDs in these sets of TFs increased to 66 (42%) and 27 (63%), respectively, when calling RDs with a more lenient threshold in RD‐seq (see Material and Methods). The remaining repressors might require specific cellular or regulatory contexts to function or contain RDs that are too weak to be detected by RD‐seq, are bipartite, and/or are longer than the 50 AA fragments we screened.

**Figure EV5:**
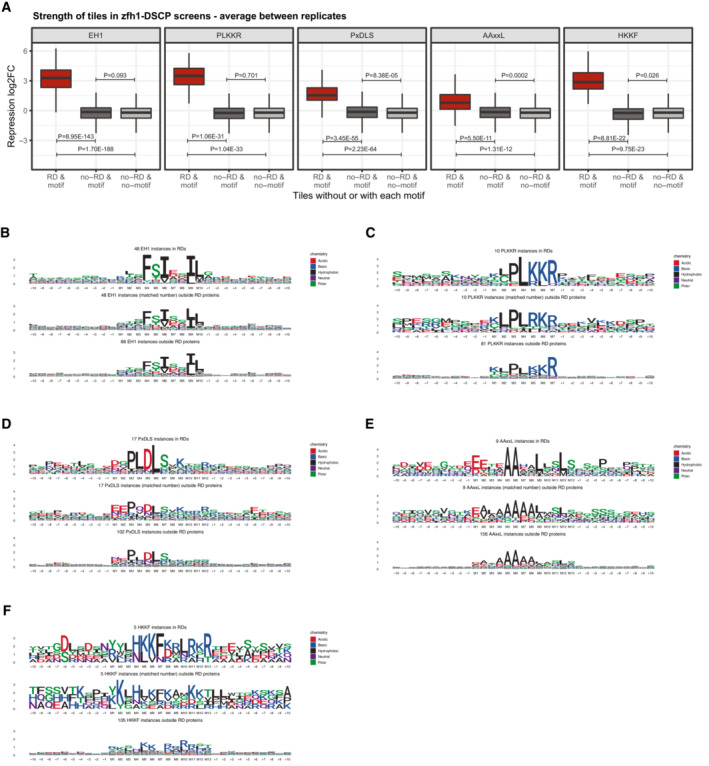
Analysis of non‐RD tiles with repressive motifs AAverage strength of tiles in RD‐seq screens with the zfh1‐DSCP reporter cell line for tiles in or outside RDs that have or do not have one of the repressive motifs indicated on the top. N of instances for RD&motif/no‐RD&motif/no‐RD&no‐motif: EH1 341/1124/204131, PLKKR 50/1109/204146, PxDLS 136/1303/203952, AAxxL 51/2327/202928, HKKF 33/1173/204082. The box plots mark the median, upper and lower quartiles and 1.5× interquartile range (whiskers). Differences between the groups of tiles were assessed with two‐sided Wilcoxon rank‐sum tests, and *P*‐values are shown within the plots.B–FAmino acid sequence logos for instances of repressive motifs within and outside RDs. For motif instances in non‐RD proteins, all instances and a number of instances matched to the number of instances within RDs are shown separately. Positions of the core motifs are indicated below the logos.

Interestingly, EH1, AAxxL, PxDLS, PLKKR, and HKKF motifs also occurred in candidate fragments that were not called as RDs. This might be due to different reasons: either these candidate fragments were repressive but did not pass the stringent threshold in RD‐seq, or the motifs occur in sequence contexts not permissive for function. Assessing the raw RD‐seq signals prior to thresholding, we observed that non‐RD tiles with repressive motifs had only background‐level signals similar to all other candidate fragments (Fig EV5A). This suggests that not all matches to repressive motifs function in any given sequence context. To assess potential differences between RD and non‐RD tiles with repressive motifs, we inspected amino acid preferences at the motif and flanking positions using sequence logos for RD and non‐RD occurrences of each of the five motifs (Fig EV5B–F). This revealed two trends: (i) functional motif instances match more stringently to the consensus motifs (Fig EV5B–F) and (ii) there seem to be amino acid preferences in the flanks of functional motif instances (e.g., serine residues surrounding the EH1 motif; Fig EV5B). As these trends were typically weak and rather diverse, and because they were only based on a small number of RD vs. non‐RD sequences, we could not derive definitive rules for the flanks of the different motifs. Determining the sequence requirements for the flanks of different repressive motifs is an interesting aim for future research.

A majority of the identified RDs (55%) contain recurrent motifs that might explain their CoR interactions and functions. The remaining 45% of RDs did not contain any of these SLiMs, suggesting that they function via rare motifs shared between only very few RDs (precluding the motifs' discovery by statistical over‐representation), via short(er) sequence patterns that are not amenable to statistical sequence analyses, or by entirely different means. Some RDs may use different motifs to recruit the same CoR, as has been described for the EH1 and the WRPW motifs that both recruit Gro (Fisher *et al*, 1996; Tolkunova *et al*, 1998). Other RDs may utilize entirely different sets of CoRs than the ones found and studied here. A recent study that maps RDs in human proteins (preprint: DelRosso *et al*, 2022) implicates short SUMOylation sites and SUMO interacting motifs in RD function, consistent with a role of SUMOylation in transcriptional repression (Ross *et al*, 2002; Rocca *et al*, 2017; Ninova *et al*, 2020; Andreev *et al*, 2022). We also find SUMOylation and SUMO interaction sites in RDs (Dataset EV8), but these motifs do not occur significantly more frequently in RDs than in other non‐RD fragments tested in the screen (Dataset EV8), presumably because such sites are short and/or have low information content and because SUMOylation may not be exclusively used in RDs. Which kind of repressive mechanisms different repressor‐CoR pairs utilize remains an open question for future research. Interestingly, we found several examples of repressors with multiple RDs harboring distinct repressive motifs and likely recruiting different CoRs (Figs 1E and EV3D). Such motif‐based modularity could allow for additive functions of transcriptional repressors.

Strikingly, the properties of RDs differ remarkably from those of tADs (Brent & Ptashne, 1985; Arnold *et al*, 2018). While many RDs contain conserved repressive motifs (Fig 4) that bind specific CoRs (Fig 3), tADs do not share recurrent motifs and are typically poorly conserved and difficult to predict (Erkina & Erkine, 2016; Erijman *et al*, 2020; Sanborn *et al*, 2021; Soto *et al*, 2022). Moreover, tADs have been described to show rather fuzzy and weak binding of their cofactors (Erijman *et al*, 2020; Sanborn *et al*, 2021) with variable binding interfaces (Sanborn *et al*, 2021). In contrast, the presence of recurrent conserved repressive motifs in RDs suggests well‐defined RD‐CoR interaction interfaces, which for some examples have indeed been described by structural studies (Nardini, 2003; Jennings *et al*, 2006; He *et al*, 2021). These differences in RD and tAD characteristics are interesting because they indicate that transcriptional activation and repression utilize different biochemical mechanisms and principles to cause opposite effects on gene expression.

Notably, the RD properties uncovered in *Dmel* are shared with human repressors: Repressive motifs found in *Dmel* are deeply conserved throughout evolution (Fig 4), and the annotation of RDs through such motifs—together with a study similar to ours for human TFs (preprint: DelRosso *et al*, 2022)—pose valuable resources for studying RDs and the impact of RD mutations, for example, in disease contexts. Understanding RDs and their interacting CoRs is particularly important at a time when interests are increasingly shifting from studying transcriptional activation toward the actors and mechanisms of transcriptional repression.

## Materials and Methods

### Reagents and Tools table

**Table:** 

Reagent/Resource	Reference or source	Identifier or Catalog number
**Experimental Models**		
Drosophila S2 cells	Thermo Fisher Scientific	R69007
**Recombinant DNA**		
ptAD‐seq‐ubi63E‐Gal4‐DBD	10.15252/embj.201798896	NA
zfh1‐DSCP‐Gal4‐DBD	This study	NA
zfh1‐DSCP‐Gal4‐dpse‐EBFP2	This study	NA
zfh1‐DSCP‐3xFLAG‐Gal4‐dpse‐EBFP2	This study	NA
zfh1‐DSCP‐GFP reporter donor	This study	NA
ent1‐rps12‐GFP reporter donor	This study	NA
sgRNA1‐Cas9	10.1038/s41594‐019‐0270‐6	based on Addgene #49330
sgRNA2‐Cas9	10.1038/s41594‐019‐0270‐6	based on Addgene #49330
**Antibodies**		
Mouse anti‐FLAG antibody	Sigma‐Aldrich	F1804‐200UG
Anti‐mouse HRP‐conjugated secondary antibody	Cell Signaling	7076
Rabbit anti‐Tubulin antibody	Abcam	ab18251
Anti‐rabbit HRP‐conjugated secondary antibody	Cell Signaling	7074
**Oligonucleotides and sequence‐based reagents**		
PCR primers	This study	Dataset EV12
Primers for RD‐seq library preparation	This study	Dataset EV12
**Chemicals, enzymes and other reagents**		
Schneider S2 cell medium	Gibco	21720024
Fetal bovine serum (FBS)	Sigma‐Aldrich	F7524
Penicillin‐Streptomycin	Gibco	15140122
MaxCyte Hyclone electroporation buffer	MaxCyte	EPB‐1
DNase I	Wothington Biochemical	LS006330
Express Five Serum‐free medium	Gibco	10486025
L‐Glutamine	Gibco	25030081
KAPA Hifi Hot Start Ready Mix	KAPA Biosystems	KK2602
In‐Fusion HD	Clontech	639650
Dynabeads Oligo‐dT25	Invitrogen	61002
TURBO Dnase	Invitrogen	AM2238
AMPure XP DNA beads	Beckman Coulter	A63881
SuperScript III Reverse Transcriptase	Invitrogen	18080085
RNase A	Thermo Fisher Scientific	EN0531
2‐Mercaptoethanol	Sigma‐Aldrich	63689
Mach1 cells	Invitrogen	C862003
Benzonase	Sigma‐Aldrich	E1014
2x Laemmli Sample Buffer	Bio‐Rad	1610737
4–15% Mini‐PROTEAN® TGX™ Precast Protein Gels	Bio‐Rad	4561083
Immun‐Blot PVDF Membrane	Bio‐Rad	1620177
Clarity Western ECL Blotting Substrate	Bio‐Rad	1705061
cOmplete™, EDTA‐free Protease Inhibitor Cocktail	Roche	COEDTAF‐RO
FLAG M2 Magnetic Beads	Sigma‐Aldrich	M8823
Lysyl Endopeptidase®, Mass Spectrometry Grade	FUJIFILM Wako Pure Chemical Corporation	121‐05063
Tris 2‐carboxyethyl phosphine hydrochloride	Sigma‐Aldrich	75259
Methyl methanethiosulfonate	Sigma‐Aldrich	64306
Trypsin Gold	Promega	V5280
Pierce™ Trifluoroacetic Acid (TFA), LC‐MS Grade	Thermo Fisher Scientific	85183
PepMap Acclaim HPLC column C18, 5 mm × 300 μm ID, 5 μm, 100 Å	Thermo Fisher Scientific	160454
PepMap Acclaim HPLC column C18, 500 mm × 75 μm ID, 2 μm, 100 Å	Thermo Fisher Scientific	164942
Q5® Hot Start High‐Fidelity 2X Master Mix	NEB	M0494L
T7 RNA Polymerase	Promega	P2075
Oligo(dt)20 primer	Invitrogen	18418020
Promega GoTaq qPCR Master Mix	Promega	A6001
**Software**		
GraphPad v9.4.1	NA	https://www.graphpad.com
BD FACS Diva v9.0	NA	https://www.bdbiosciences.com/
R (v3.5.1)	NA	https://www.r‐project.org/
Bowtie v.1.2.2	Langmead *et al* (2009)	https://bowtie‐bio.sourceforge.net/index.shtml
UCSC Genome Browser	Kent *et al* (2002)	https://genome.ucsc.edu/
ProSitePatterns	de Castro *et al* (2006)	https://prosite.expasy.org/
Pfam	Mistry *et al* (2021)	https://pfam.xfam.org/
MobiDB‐lite	Necci *et al* (2021)	http://old.protein.bio.unipd.it/mobidblite/
MEME v.5.1.1	Bailey *et al* (2015)	https://meme‐suite.org/meme/
TOMTOM v.5.4.1.	Gupta *et al* (2007)	https://meme‐suite.org/meme/tools/tomtom
FIMO searches v.5.4.1.	Grant *et al* (2011)	https://meme‐suite.org/meme/tools/fimo
ELM database (2022)	Kumar *et al* (2019)	http://elm.eu.org/
Proteome Discoverer v2.5.0.400	Thermo Fisher Scientific	https://www.thermofisher.com/
MSAmanda v2.0.0.16129	Dorfer *et al* (2014)	https://ms.imp.ac.at/index.php?action=home
Tool ptmRS, based on the tool phosphoRS	Taus *et al* (2011)	https://ms.imp.ac.at/index.php?action=home
apQuant	Doblmann *et al* (2018)	https://ms.imp.ac.at/index.php?action=home
Intensity‐based absolute quantification (iBAQ)	Schwanhäusser *et al* (2011)	https://ms.imp.ac.at/index.php?action=home
mafft (‐linsi, v7.427)	Katoh & Toh (2008)	https://mafft.cbrc.jp/alignment/software/
Jalview	Waterhouse *et al* (2009)	https://www.jalview.org/
Orthofinder v2.5.4	Emms & Kelly (2019)	https://github.com/davidemms/OrthoFinder
AAcon v1.1	Golicz *et al* (2018)	https://www.compbio.dundee.ac.uk/aacon/
**Other**		
MaxCyte STX Scalable Transfection System	MaxCyte	
BD FACSAria III cell sorter	BD Biosciences	
Qiagen RNeasy Mini Prep Kit	Qiagen	
Illumina NextSeq550 system	Illumina	
FACS BD LSR Fortessa	BD Biosciences	
NEB Monarch Gel Extraction kit	NEB	
NEB Monarch Nucleic Acid kit	NEB	
Power Blotter XL	Invitrogen	
ChemiDoc MP imaging system	Bio‐Rad	
Diagenode Bioruptor Sonicator	Diagenode	
UltiMate™ 3000 RSLCnano System nano HPLC system	Thermo Fisher Scientific	
Exploris 480 mass spectrometer with a FAIMS pro interfaces and a Nanospray Flex ion source	Thermo Fisher Scientific	
Invitrogen MEGAclear Transcription Clean‐Up Kit	Invitrogen	

### Methods and Protocols

#### RD candidate expression plasmids

##### RD‐seq plasmid backbone

The plasmid backbone for the RD‐seq candidate library was derived from ptAD‐seq‐ubi63E‐Gal4‐DBD (Arnold *et al*, 2018) by replacing the ubi63E enhancer with the zfh1 enhancer (from pGL3_zfh1_CP‐candidate_luc+; Addgene 86391) in between the KpnI (Thermo) and BglII (Thermo) restriction sites (Dataset EV2, RD‐seq backbone: zfh1‐DSCP‐Gal4‐DBD, primers in Dataset EV12). The plasmid contains the Gal4‐DBD followed by a poly‐glycine linker upstream of the candidate library insertion site, which consists of the *ccdB* suicide gene flanked by homology arms, which is followed by three stop codons. For details on how candidate fragments were integrated into the RD‐seq backbone, see *Candidate tiling library design and cloning*.

##### Validation plasmid backbone

For validation experiments, we introduced the fluorescent protein EBFP2 (source: Addgene 54665) in the RD‐seq plasmid backbone to be able to gate for transfected cells in flow cytometry (Dataset EV2, validation backbone: zfh1‐DSCP‐Gal4‐dpse‐EBFP2). As drivers for EBFP2 expression, we used the combination of the dpse‐102 enhancer together with the CG13116 promoter, which was utilized for strong expression in S2 cells in a previous study (Haberle *et al*, 2019). Dpse‐102 is an enhancer found in *Drosophila pseudoobscura* that drives strong expression in S2 cells (Arnold *et al*, 2014), and the CG13116 promoter is the promoter of the *Drosophila pseudoobscura* ortholog of the CG13116 gene and has been found to be strongly inducible in S2 cells (Arnold *et al*, 2017). An oligonucleotide with the EBFP2 gene, a stop codon and the SV40 poly‐A site synthesized by IDT (Dataset EV12: EBFP2‐stop‐polyA) was amplified with primers including overhangs for Gibson cloning (Dataset EV12: EGFP2_fw and _rv). The dpse‐102 enhancer and the CG13116 promoter were amplified from pAGW‐dpse‐GAL4‐DBD (Addgene 125153) with primers including overhangs for Gibson cloning (Dataset EV12: dpse‐CG13116‐promoter_fw and _rv). Using Gibson assembly (NEB), both fragments were integrated into the LguI‐linearized (Thermo) RD‐seq plasmid.

##### FLAG‐tag plasmid backbone

For testing the expression of mutated RDs in western blots and for IP‐MS experiments, the validation construct was further modified by introducing a sequence containing the 3xFLAG‐tag and a short Gly‐Ser linker upstream of the Gal4‐DBD (Dataset EV2; FLAG backbone: zfh1‐DSCP‐3xFLAG‐Gal4‐dpse‐EBFP2). To introduce “3xFLAG‐linker,” we performed a mutagenesis PCR using the primers [Phos]ATCGATTACAAGGATGACGATGACAAGGGTGGTGGTGGTAGTATGAAGCTACTGTCTTCTATCGAA and [Phos]GTCATGATCTTTATAATCACCGTCATGGTCTTTGTAGTCCATTTTGAAGTGGCCTGAAGTAAAGGA and the validation plasmid as template (25 μl KAPA HiFi HotStart ReadyMix (KAPA Biosystems KK2602), 1 μl 100 μM forward primer, 1 μl 100 μM reverse primer, template (10 ng/μl), 22 μl double‐deionized water; PCR conditions: 95°C 3 min, followed by 21 cycles, 98°C 20 s, 65°C 15 s, 72°C 6 min, and final extension 7 min).

After the PCR, the template plasmid was digested using DpnI (Thermo), followed by ligation of the overhanging ends and transformation into Mach1 (Thermo) bacterial cells.

To generate RD expression plasmids with the validation or FLAG backbone, RD fragments amplified from *Drosophila* embryonic cDNA were integrated between SgrDI (Thermo) and BsHTI (Thermo) restriction sites in the respective backbone plasmid via Gibson assembly (NEB) according to the manufacturer's protocol. “Gal4‐DBD control” constructs without an RD were created by annealing the two oligonucleotides CCGGCTGAAGTTGAG and TCGACTCAACTTCAG, encoding two stop codons, and inserting the resulting fragment in between the SgrDI and BsHTI restriction sites of the plasmid backbone.

#### *Drosophila* S2 cell culture and cell line generation

*Drosophil*a S2 cells were cultured as described before (Arnold *et al*, 2013).

To generate *Drosophila* S2 reporter cell lines, we integrated reporter constructs with 100‐bp upstream and downstream homology arms into the integration site at chr2L:9094918, which does not contain any genes, by CRISPR‐Cas9. The reporter constructs contained 14 UAS sites for Gal4‐DBD binding (source: Addgene 128010), an enhancer and a core promoter, the EGFP gene and the SV40 poly‐A site. We created 2 different reporters in which EGFP was driven by (i) the zfh1 enhancer and the *Drosophila* synthetic core promoter (zfh1‐DSCP) or (ii) the ent1 enhancer and the rps12 core promoter (ent1‐rps12; Dataset EV2). The zfh1 and the ent1 enhancers were found in a previous study mapping enhancers in *Dmel* with STARR‐seq testing enhancer candidates together with different promoters (Zabidi *et al*, 2015). The zfh1 enhancer is located within the *zfh1* gene and is a strong enhancer together with the DSCP, while the ent1 enhancer is located upstream of the gene and strongly activates rps12 (Zabidi *et al*, 2015). Elements can be found in Dataset EV2.

Two plasmids (based on the gRNA expression plasmid Addgene #49330) encoding Cas9 and single‐guide RNAs (sgRNA1: TGCCACATGCAACGCGGAGT, sgRNA2: GCGGAGTTGGAGTTTTGTAT) targeting the integration site were kindly received from the Brennecke Lab at IMBA Vienna (Batki *et al*, 2019). For the CRISPR‐Cas9‐mediated integration of the reporters, 50 × 10^6^ *Drosophila* S2 cells were co‐transfected with 3.5 μg reporter plasmid and 2.5 μg of each gRNA plasmid using the MaxCyte STX Scalable Transfection System. Cells were passaged for 7 days before the selection of GFP‐positive cells via fluorescent‐activated cell sorting (FACS) and plating in single‐cell dilutions for generating clonal cell lines. Cells were genotyped using primers binding upstream and downstream of the integration site (Dataset EV12: Chr2L_fw and _rv), and homozygous clones were selected.

#### Candidate tiling library design and cloning

Candidates for the tiling library were selected based on FlyTF, a database for known and putative *Drosophila melanogaster* transcription factors (Pfreundt *et al*, 2010) in which TFs are scored based on the presence of a DNA‐binding domain and experimental evidence for a function in transcription (score of 1–8, with score 1 for the most confident candidates). We used a version of the FlyTF database with 1,168 proteins (downloaded on December 5, 2018, for list refer to Dataset EV1). Of all 1,168 FlyTF proteins, 1,133 factors were selected and 150‐bp oligonucleotides were designed to tile the transcripts of these proteins (sliding windows of 6 nt for genes with FlyTF score of 1–4 and sliding window of 15 nt for genes with score of 5–8). This resulted in 209,495 distinct 150‐bp candidate fragments. The FlyTF genes, FlyTF scores, the candidate genes we selected, and the candidate fragments we designed can be found in Dataset EV1.

The library was cloned from a pool of 209,495 200 bp oligonucleotides synthetized by Twist Biosciences. Each oligonucleotide contained the 150‐bp candidate sequence described above flanked by the 25 bp of the partial Illumina i5 (TCCCTACACGACGCTCTTCCGATCT) and 25 bp of the partial i7 (AGATCGGAAGAGCACACGTCTGAAC) adaptor sequences serving as constant linkers for amplification and cloning. The oligonucleotide pool (diluted to 1 ng/μl) was amplified in 40 PCR reactions (98 °C for 45 s; followed by 14 cycles of 98 °C for 15 s, 65 °C for 30 s, and 72 °C for 10 s) using KAPA Hifi Hot Start Ready Mix (KAPA Biosystems KK2602) and primers (fw: TTGAGCATGCACCGGACACTCTTTCCCTACACGACGCTCTTCCGATCT and rev: ATCTATCTACGTCGAGTGACTGGAGTTCAGACGTGTGCTCTTCCGATCT) that extended the i5 and i7 adaptor sequences to the full length and added extra 15 bp to each of the adapters, serving as homology arms for directional cloning of the library into the RD‐seq plasmid (zfh1‐DSCP‐Gal4‐DBD, Dataset EV2) vector using In‐Fusion HD (Clontech 639650).

#### RD‐seq pipeline, RNA processing, and Illumina sequencing

*Drosophila* S2 reporter cells, cultured at 70–80% confluence, were transfected with the candidate library using the MaxCyte STX Scalable Transfection System. For one screen, seven OC‐400 processing assemblies were prepared with 200 × 10^6^ cells each in 400 μl MaxCyte Hyclone buffer mixed 1:1 with S2 culture medium without supplements and with 20 μg of the library. In total, for one screen 1.4 × 10^9^ cells were transfected with 140 ug library and for each reporter cell line (zfh1‐DSCP and ent1‐rps12) two biological replicates of RD‐seq screens were performed. S2 cells were electroporated with the preset protocol “Optimization 1,” subsequently mixed with 40 μl DNase I (2,000 U/ml) in a T175 cell culture flask, incubated for 30 min at 27°C, and resuspended in 30 ml complete S2 cell medium.

Three days after transfection, cells were separated into fractions of GFP‐positive and GFP‐negative cells via fluorescent‐activated cell sorting (FACS) on a BD FACSAria III cell sorter. For each experiment, 30 × 10^6^ GFP‐positive cells and approximately 8 × 10^6^ GFP‐negative were collected.

Total RNA of the different fractions was isolated using the Qiagen RNeasy Mini Prep Kit, followed by Poly‐A+ RNA enrichment with Dynabeads Oligo‐dT25 (Invitrogen) and a DNA digest with TURBO Dnase (Ambion). After RNA cleanup with AMPure XP DNA beads (Agencourt; ratio sample/beads 1:1.8), reverse transcription was performed with Superscript III (50°C for 60 min, 70°C for 15 min; Invitrogen 18080085) and a primer binding within the poly‐A site of candidate mRNAs (reverse_transcription_rv: CTCATCAATGTATCTTATCATGTCTG). Next, RNA was digested with RNase A (Thermo) for 1 h at 37°C, followed by bead cleanup of the cDNA (ratio sample/beads 1:1.4). All subsequent PCR reactions were prepared using the KAPA HiFi HotStart ReadyMix (KAPA Biosystems KK2602). A second strand PCR was performed with a primer binding upstream of the intron sequence, which is part of candidate mRNAs (2^nd^_strand_primer_fw: TTGGTAAAGCCACCATGGAAAAG*G; 98°C for 60 s, 65°C for 30 s, and 72°C for 90 s), followed by bead cleanup (ratio sample/beads 1:1.4). In the next step, unique molecular identifiers (UMIs) were introduced to the 3′ ends of DNA fragments in a linear PCR with a primer binding to the Illumina i7 adaptor sequence (UMI_primer_rv: CAAGCAGAAGACGGCATACGAGATNNNNNNNNNNGTGACTGGAGTTCAGACGTGT*G; 98°C for 60 s, 65°C for 30 s, 72°C for 90 s). After bead cleanup (ratio sample/beads 1:1.4), the generated fragments were PCR‐amplified (98°C 45 s, followed by 16 cycles, 98°C 15 s, 65°C 30 s, 72°C 70 s) using two candidate‐specific primers (junction_PCR_fw: AAGCCACCATGGAAAAG*G*C*C*A*T and junction_PCR_rv: CAAGCAGAAGACGGCATACG*A), one of which spans the splice junction of the mhc16 intron (5 and 1 nucleotides at the 3’ ends are protected by phosphorothioate bonds, respectively). After another bead cleanup (ratio sample/beads 1:1), candidate fragments were amplified (98°C 45 s, followed by 6–15 cycles, 98°C 15 s, 65°C 30 s, and 72°C 70 s) with the following primers: i5: aatgatacggcgaccaccgagatctacacXXXXXXXXacactctttccctacacgacgctcttccgatct (XXXXXXXX indicates the position of the index sequence for NGS; for i5 primers used in individual screens, see Dataset EV12) and the reverse primer seq_ready_rv: CAAGCAGAAGACGGCATACGAGA*T. PCR products were purified by Agencourt AMPure XP DNA beads (ratio sample/beads 1:0.9), pooled, and subjected to NGS.

All samples were paired‐end sequenced (PE36) by the NGS unit of the Vienna Biocenter Core Facilities GmbH (VBCF) on an Illumina NextSeq550 system, following the manufacturer's protocol.

#### Computational analysis of RD‐seq hits

##### Creation of dedicated bowtie index

A bowtie index was generated from the designed 150‐bp (50 amino acids) oligo sequences, flanked by upstream (“TCCCTACACGACGCTCTTCCGATCT”) and downstream (“AGATCGGAAGAGCACACGTCTGAAC”) adapters. This genome was used to create a custom bowtie index using Bowtie v.1.2.2 (Langmead *et al*, 2009). For visualization purposes in UCSC Genome Browser, we also created a linear genome containing selected ordered TFs, separated by 2,100 N's.

##### NGS read mapping and processing

Paired‐end sequencing reads were demultiplexed using specific barcodes and mapped to the dedicated bowtie index using Bowtie v.1.2.2 (Langmead *et al*, 2009; ‐X 150 ‐v 3 ‐m 1 ‐quiet ‐best ‐strata). The UMI sequence was incorporated into the read ID at the demultiplexing step. Mapped read pairs, fragments, were collapsed by oligoID and by UMI, that is, by removing duplicate fragments with identical coordinates if their UMIs differed by <= 2 out of the 10 nucleotides. To calculate position‐specific coverage for each frame, oligonucleotide‐centric coordinates were transformed into TFs‐centric coordinates and total coverage was calculated using the coverage function from R package GenomicRanges v.1.32.7 (Lawrence *et al*, 2013). Fragment coverage was visualized using the linear genome in the UCSC Genome Browser (Kent *et al*, 2002).

We calculated enrichments, hypergeometric *P*‐values, and Benjamini–Hochberg (BH)‐corrected false discovery rates [FDRs; all statistical calculations done in R (R Development Core Team, 2008)] between the coverage values in GFP− and GFP+ cells. To define repressive domain (RD) regions, we only considered regions with a minimal coverage of at least 10 fragments in GFP+ and GFP‐ cells and selected regions with a minimal enrichment of ≥1.5‐fold and a hypergeometric *P*‐value of ≤ 1 × 10^−5^ across a minimal length of ≥60 bp (20 amino acids), which we extended to include flanking coding sequences (CDS) until *P* > 1 × 10^−3^ over ≥60 bp (20 amino acids).

##### Intersection of RD‐seq hits

For each reporter cell line (zfh1‐DSP and ent1‐rps12), two replicate RD‐seq screens were performed. NGS mapping statistics for each screen can be found in (Dataset EV13). After determining RD regions for each RD‐seq screen, the hits of two replicates were intersected and only repressive regions detected in both replicates with a minimum overlap of 50% were kept for further analysis. Next, repressive regions from the screens with the zfh1‐DSCP and the ent1‐rps12 reporter cell line were intersected (RD regions with sequence overlaps of 50%, keeping only the longest RD) resulting in 195 unique RD regions, which were either detected using both reporter cell lines or only in one of the two. We recalculated the enrichments of each RD region in each screen to compare their strength between screens and reporters. Information on all 195 RDs can be found in Dataset EV3.

#### Assessment of sensitivity and specificity of RD‐seq

The lack of systematic annotations of RDs in fly TFs makes it difficult to evaluate the specificity and sensitivity of RD‐seq against an independent benchmark dataset. However, the candidate library contained fragments covering 438 TFs whose regulatory activity was assessed in a previous study (Stampfel *et al*, 2015). While many TFs could function as repressors in one of the 24 contexts tested by Stampfel *et al* (2015), we defined as repressors TFs that were consistently repressive (sum of scores across all contexts < ‐20) or strongly repressive (< ‐35), leading to 156 or 43 TFs, respectively. To allow the assessment of specificity, we additionally defined weakly repressive TFs and nonactivators as TFs with sum of scores of < ‐10 and ≤0, respectively. To allow the assessment of sensitivity, we additionally called RDs with a more lenient cutoff of (hypergeometric *P*‐value ≤ 1 × 10^−3^, minimal enrichment ≥ 1.2‐fold). The TFs from Stampfel *et al* (2015) and RDs detected within these TFs in RD‐seq with different cutoffs can be found in Dataset EV18.

#### RD validations

To validate RD‐seq hits, we cloned one of the most highly enriched 150‐bp candidates per RD region (sequences in Dataset EV4) into the Gal4‐DBD validation plasmid backbone zfh1‐DSCP‐Gal4‐dpse‐EBFP2 (described in *RD candidate expression plasmids*). All Gibson overhang primers used for the individual RDs can be found in Dataset EV12. A 25 × 10^6^ reporter cells in 50 μl MaxCyte Hyclone buffer mixed 1:1 with S2 culture medium without supplements were transfected with 2.5 μg Gal4‐DBD‐RD or Gal4‐DBD control plasmid using OC‐100 processing assemblies and the MaxCyte STX Scalable Transfection System on “Optimization 1.” After electroporation, cells were resuspended in 5 μl DNase I (2,000 U/ml) in a T25 cell culture flask, incubated for 30 min at 27°C, and resuspended in 5 ml complete S2 cell medium.

Three days after transfection, cells were submitted to flow cytometry analysis using a FACS BD LSR Fortessa (BD Biosciences). The GFP signal of transfected cells, gated based on EBFP2 expression as transfection control, was determined, and data analysis was performed with FACS Diva. As a measure of the repressive strength of the RD, we used the ratio of the medians between the GFP signal of cells expressing a Gal4‐DBD control construct without an RD and cells expressing the Gal4‐DBD‐RD and called it fold change (FC) repression (FC repression = median‐GFP[Gal4‐DBD control]/ median‐GFP[Gal4‐DBD‐RD]).

To assess the significance of RDs in the main validation set, we used two‐tailed, paired Student's *t*‐tests comparing the median log2 GFP values of the Gal4‐control to the Gal4‐RD condition for three independent biological replicates (*P* ≤ 0.05; FC >1 for validated). To determine differences between wild‐type and mutant RDs, we used two‐tailed, paired Student's *t*‐tests comparing the log2 of the FC repression values of wild‐type and mutant RDs of three independent biological replicates (*P* ≤ 0.05; FC >1 for validated). FC repression values from individual replicates and *P*‐values of the *T*‐tests can be found in Dataset EV4.

#### Analysis of RD and DBD positioning within full‐length proteins

We used the centered amino acid of each 50 AA RD (RD‐seq) and DBD (from ProSitePatterns and Pfam) as their position within the full‐length TF sequences, scaled over the length of the respective protein sequences to be comparable across proteins. To analyze DBD positioning, we only considered DBDs appearing in proteins that have an RD region according to RD‐seq (Dataset EV14).

#### Analysis of RD overlaps with known domains and IDRs

We used the full‐length protein sequences of all proteins for which an RD was detected in RD‐seq as input for ProSitePatterns (de Castro *et al*, 2006), Pfam (Mistry *et al*, 2021), and MobiDB‐lite (Necci *et al*, 2021) protein domain database searches. To assign a ProSitePatterns, Pfam, or MobiDB‐lite hit to an RD, we only selected those cases in which the RD (=50 AA most strongly enriched candidate fragment within the RD region) contains at least 50% of the domain or in which at least 50% of the RD is part of the annotated domain. ProSitePatterns and Pfam entries from protein families, not relevant for protein domain analysis, were removed. The resulting domain–RD overlaps can be found in Dataset EV5.

The prevalence of IDRs from the MobiDB‐lite database in RDs was compared with the prevalence of IDRs among 50 amino acid fragments (same size as the RDs) from *Dmel* transcription‐related proteins or all *Dmel* proteins (excluding sequences that overlap RDs), using the same overlap rules described above.

#### MEME and FIMO peptide motif searches among RD‐seq hits

The most repressive 150‐bp candidate fragments (= 50 AA‐long RDs) were used for MEME *de novo* motif analyses. For that, four different sets of RDs were created based on the preference of an RD region for the zfh1‐DSCP or the ent1‐rps12 reporter context. Preferences for one of the reporters were calculated by dividing the mean FC of the RD region detected in the RD‐seq screens using one reporter over the FC resulting from the RD‐seq screens with the other reporter. Subset information can be found in Dataset EV3 in the column “RD.region.preference.1.3fold.” RD regions with a > 1.3‐fold preference for the zfh1‐DSCP context were categorized as “zfh1” hits, while RD regions with a > 1.3‐fold preference for the ent1‐rps12 reporter were categorized as “ent1” hits. RDs without a preference were categorized as “global” hits. This resulted in four different RD sets that were separately subjected to MEME *de novo* motif searches (Bailey *et al*, 2015): (i) 195 RDs (all hits), (ii) 89 RDs without a preference, (iii) 43 zfh1 RDs, (iv) 63 ent1 RDs.

We ran MEME v.5.1.1 (Bailey *et al*, 2015) with the following parameters: *‐protein ‐oc*. *‐nostatus ‐time 18000 ‐mod zoops ‐nmotifs 25 ‐minw 4 ‐maxw 15 ‐objfun classic ‐markov_order 0*. This resulted in 22 motifs in each set with motif widths between 4 and 15 AA. Two motifs were removed since the enrichment derived solely from paralog proteins. To collapse redundant motifs by similarity, we computed the distances between all motif pairs using TOMTOM (kullback distance; Gupta *et al*, 2007) and performed hierarchical clustering using Pearson correlation as the distance metric and complete linkage using the hclust R function. The tree was cut at height 0.7, resulting in 11 nonredundant motif clusters that were manually annotated (Fig 2C; Dataset EV6). Some of the motifs were detected in multiple RD sets (e.g., EH1 motif was found in MEME searches with zfh1, global, and all RDs, see Fig 2C). Hence, for subsequent analysis, we selected one motif per group: Motif 1—ent1, Motif 2—all, Motif 3—global, Motif 4—global, Motif 5—global, Motif 6—zfh1, Motif 7—all, Motif 8—zfh1, Motif 9—all, Motif 10—all, and Motif 11—ent1. These MEME motifs were used as input for FIMO searches (v.5.4.1.) (Grant *et al*, 2011) with a stringent (*P* < 0.0001) or a lenient (*P* < 0.001) cutoff to determine the prevalence of the peptide motifs among all 195 RD‐seq hits. The results of the FIMO searches can be found in Dataset EV6. For the visualization of motif instances among RDs (Fig 2E), only instances from the FIMO searches with the stringent cutoff were used.

The same FIMO motif searches were performed across all non‐RD tiles to identify the ones with instances of repressive peptide motifs.

#### Amino acid sequence logos for instances of repressive motifs within and outside RDs

Motif instances in RDs or outside RD proteins were selected, and their extended sequences (motif core ± 10 amino acid) retrieved. For each set of motif instances, we calculated the frequency of each amino acid at each position and visualized it as amino acid sequence logos using the ggseqlogo function from the R package ggseqlogo (v.0.124).

#### Analysis of known SLiMs within RDs using the ELM prediction tool

We used the most repressive 150‐bp candidate fragment (= 50 AA‐long RDs) within each of the 195 RD regions detected in RD‐seq as input for ELM database searches for short linear motifs (SLiMs; Kumar *et al*, 2019; Dataset EV8). Next, we used the list of matches to high‐probability ELM patterns (*P* < 0.0002) and filtered for SLiMs that have been implicated in the interaction with co‐repressors: the EH1 motif (LIG_EH1_1), the WRPW motif (LIG_WRPW_2), the CtBP ligand motif (LIG_CtBP_PxDLS_1), the Sin3A‐interacting domain (LIG_Sin3_1), and the HCF‐1‐binding motif (LIG_HCF‐1_HBM_1; Dataset EV8).

The enrichment of each type of ELM pattern in RDs was quantified over all non‐RD nonoverlapping 50 amino acid tiles by two‐sided Fisher's exact test (Dataset EV8).

#### Analysis of known and novel SLiMs within RDs

We characterized the motif composition of each RD by integrating both annotated (from ELM) and *de novo* (from MEME) SLiMs (Fig 2D). We categorized an RD as having a known SLiM instance if containing an instance from ELM (CoR‐interacting motifs), while the remaining RDs with instances from MEME analysis (instances from FIMO with *P* < 0.00001, excluding motifs 6 and 10) not reported in ELM were considered as novel instances. The remaining RDs without any of these SLiMs were considered as unexplained.

#### Site‐directed mutagenesis of RD peptide motifs

To determine the requirement of peptide motifs discovered in MEME and FIMO searches for the function of RDs, residues within these motifs were mutated to Alanines (5 AA mutated to Ala in case of EH1, PXDLS, AAxxL, and PLKKR motifs, and 4 AA in case of the HKKF motif). The Gal4‐DBD‐RD validation plasmids with the wild‐type RD sequences were subjected to site‐directed mutagenesis using primers carrying the mutated version of the motifs in overhangs (primers see Dataset EV12). After PCR amplification with the KAPA HiFi HotStart ReadyMix (KAPA Biosystems KK2602; 95°C 3 min, followed by 21 cycles, 98°C 20 s, 65°C 15 s, 72°C 6 min, and final extension 7 min), amplicons were purified using the NEB Monarch Gel Extraction kit and template plasmids were DpnI‐digested (Thermo) followed by cleanup with the NEB Monarch Nucleic Acid kit. The ends created by the overhang primers were ligated, and Mach1 cells (Thermo) were transformed with the resulting plasmids. Mutated Gal4‐DBD‐RD constructs were used in validation experiments as described above. Wild‐type and mutant RD sequences and the validation results can be found in Dataset EV4.

#### Assessing RD expression in western blots

To monitor the expression of mutated RDs in comparison with the wild‐type RDs, wild‐type and mutant RDs were cloned into the FLAG‐Gal4‐DBD background (zfh1‐DSCP‐3xFLAG‐Gal4‐dpse‐EBFP2, Dataset EV2) as described under *RD candidate expression plasmids*. The zfh1‐DSCP reporter cell line was transfected with the FLAG‐Gal4‐DBD‐RD plasmids according to *RD validations*. Three days after transfection, 3 × 10^6^ cells were harvested, washed with PBS, and lysed in 30 μl lysis buffer (10 mM Tris pH8, 1 mM EDTA, 0.5 mM EGTA, 1% Triton x‐100, 0.1% SDS, 0.1% sodium deoxycholate, 140 mM NaCl, Roche cOmplete Protease Inhibitor, Benzonase; Sigma, 2.5 Units/μl) for 10 min on ice. 30 μl 2x Laemmli Sample Buffer (Bio‐Rad) with 5% b‐mercaptoethanol was added to the sample followed by incubation at 95°C for 5 min. Proteins were separated using SDS‐polyacrylamide gel electrophoresis (Bio‐Rad) and subsequently blotted (Power Blotter XL, Invitrogen) onto a PVDF membrane (Bio‐Rad). The membrane was blocked with 5% milk in TBS‐T (TBS with 1% Tween‐20) and incubated overnight at 4°C with the primary anti‐FLAG antibody (Sigma F1804‐200UG, 1:1,000 in 2.5% milk in TBS‐T). The membrane was washed three times with TBS‐T, followed by 1 h incubation with the HRP‐conjugated secondary antibody (Cell Signaling 7076 S, 1:10,000 in 2.5% milk in TBS‐T). After three washes in TBS‐T, the membrane was incubated with Clarity Western ECL Blotting Substrate (Bio‐Rad) and imaged with a ChemiDoc MP imaging system (Bio‐Rad). For a loading control, blots were probed with a primary anti‐Tubulin antibody (Abcam, ab18251).

#### Immunoprecipitation‐mass spectrometry (IP‐MS) experiments

RDs were cloned into the zfh1‐DSCP‐3xFLAG‐Gal4‐dpse‐EBFP2 plasmid backbone as described under *RD candidate expression plasmids*. Plasmids encoding RDs with a specific peptide motif were mixed in an equal molar ratio to create RD plasmid pools (PxDLS: CG42741‐RD, Tio‐RD, Ham‐RD, CG11122‐RD1; AAxxL: CG11617‐RD2, Cic‐RD2, Glut4EF‐RD, CG12605‐RD; PLKKR: Ash1‐RD, Kr‐h1‐RD2, Net‐RD, Vri‐RD; HKKF: Eip75B‐RD, CHES‐1‐like‐RD1, Kah‐RD, Shn‐RD1). As a control, we used a 3xFLAG‐Gal4‐DBD construct without an RD sequence. We prepared three independent biological replicates per condition. For each replicate of an IP‐MS experiment, 200 × 10^6^ *Drosophila* S2 cells in 400 μl MaxCyte Hyclone buffer mixed 1:1 with S2 culture medium without supplements were transfected with 30 μg 3xFLAG‐Gal4‐DBD control plasmid or 30 μg of an RD plasmid pool using OC‐400 processing assemblies and the MaxCyte STX Scalable Transfection System on “Optimization 1.” After electroporation, cells were resuspended in 40 μl DNase I (2,000 U/ml) in a T175 cell culture flask, incubated for 30 min at 27°C, and resuspended in 30 ml complete S2 cell medium.

One day after transfection, cells were harvested, washed in PBS, and incubated in buffer A (10 mM Tris pH 7.5, 2 mM MgCl_2_, 3 mM CaCl2, Sigma cOmplete EDTA‐free Protease Inhibitor Cocktail) for 15 min at 4 °C followed by centrifugation. The pellet was resuspended and incubated for 30 min at 4 °C in buffer B (10 mM Tris pH 7.5, 2 mM MgCl_2_, 3 mM CaCl2, 0.5% IGEPAL CA‐630, 10% Glycerol, 1 mM DTT, Sigma cOmplete EDTA‐free Protease Inhibitor Cocktail). After centrifugation, the nuclear pellet was resuspended in buffer C (40 mM HEPES pH 7.6, 4 mM MgCl_2_, 0.6% Triton X‐100, 0.5% IGEPAL CA‐630, 20% Glycerol, 1 mM DTT, Sigma cOmplete EDTA‐free Protease Inhibitor Cocktail) with 100 mM NaCl and incubated for 30 min at 4 °C, followed by centrifugation. The supernatant containing the nucleoplasm was collected, and the remaining chromatin pellet was resuspended in buffer C with 300 mM NaCl and subjected to sonication with a Diagenode Bioruptor Sonicator for 10 min at low intensity. After centrifugation, the supernatant was transferred to the nucleoplasmic fraction. FLAG M2 Magnetic Beads (Sigma, M8823) were equilibrated in buffer C with 150 mM NaCl. Nuclear lysate was added to the beads for immunoprecipitation overnight at 4 °C. Afterward, the beads were washed three times in buffer C with 150 mM NaCl, followed by four washes in nondetergent buffer (20 mM Tris pH 7.5, 130 mM NaCl).

Beads were resuspended in 80 μl of 100 mM ammonium bicarbonate (ABC), supplemented with 800 ng of lysyl endopeptidase (Lys‐C, Fujifilm Wako Pure Chemical Corporation), and incubated for 4 h on a Thermo‐shaker with 1,200 rpm at 37°C. The supernatant was transferred to a fresh tube and reduced with 1 mM Tris 2‐carboxyethyl phosphine hydrochloride (TCEP, Sigma) for 30 min at 60°C and alkylated in 4 mM methyl methanethiosulfonate (MMTS, Fluka) for 30 min at room temperature. Subsequently, the sample was digested with 800 ng trypsin (Trypsin Gold, Promega) at 37°C overnight. The digest was acidified by the addition of trifluoroacetic acid (TFA, Pierce) to 1%. A similar aliquot of each sample was analyzed by LC–MS/MS.

##### nanoLC‐MS/MS analysis

The nano HPLC system (UltiMate 3000 RSLC nano system, Thermo Fisher Scientific) was coupled to an Exploris 480 mass spectrometer equipped with a FAIMS pro interfaces and a Nanospray Flex ion source (all parts Thermo Fisher Scientific). Peptides were loaded onto a trap column (PepMap Acclaim C18, 5 mm × 300 μm ID, 5 μm particles, 100 Å pore size, Thermo Fisher Scientific) at a flow rate of 25 μl/min using 0.1% TFA as mobile phase. After 10 min, the trap column was switched in line with the analytical column (PepMap Acclaim C18, 500 mm × 75 μm ID, 2 μm, 100 Å, Thermo Fisher Scientific) operated at 30°C. Peptides were eluted using a flow rate of 230 nl/min, starting with the mobile phases 98% A (0.1% formic acid in water) and 2% B (80% acetonitrile, 0.1% formic acid) and linearly increasing to 35% B over the next 120 min.

The Exploris mass spectrometer was operated in data‐dependent mode, performing a full scan (m/z range 350–1,200, resolution 60,000, target value 1E6) at three different compensation voltages (CV‐45, ‐60, ‐75), followed each by MS/MS scans of the most abundant ions for a cycle time of 0.9 (CV ‐45, ‐60) or 0.7 (CV ‐75) seconds per CV. MS/MS spectra were acquired using a collision energy of 30, isolation width of 1.0 m/z, resolution of 30.000, target value of 2E5 and intensity threshold of 2.5E4, and maximum injection time of 100 ms. Precursor ions selected for fragmentation (include charge state 2–6) were excluded for 45 s. The monoisotopic precursor selection filter and exclude isotopes feature were enabled.

##### IP‐MS data processing

For peptide identification, the RAW files were loaded into Proteome Discoverer (version 2.5.0.400, Thermo Scientific). All MS/MS spectra were searched using MSAmanda v2.0.0.16129 (Dorfer *et al*, 2014). The peptide and fragment mass tolerance was set to ±10 ppm, and the maximal number of missed cleavages was set to 2, using tryptic enzymatic specificity without proline restriction. Peptide and protein identification was performed in two steps. For an initial search, the RAW files were searched against the database dmel‐all‐translation‐r6.43.fasta (Flybase.org, 22232 sequences; 20,321,723 residues), supplemented with common contaminants and sequences of tagged proteins of interest, using the following search parameters: beta‐methylthiolation of cysteine was set as a fixed modification, oxidation of methionine as variable modification. The result was filtered to 1 % FDR on protein using the Percolator algorithm (Käll *et al*, 2007) integrated into Proteome Discoverer. A subdatabase of proteins identified in this search was generated for further processing. For the second search, the RAW files were searched against the created subdatabase using the same settings as above plus considering additional variable modifications: Phosphorylation on serine, threonine, and tyrosine, deamidation on asparagine and glutamine, and glutamine to pyro‐glutamate conversion at peptide N‐terminal glutamine, and acetylation on protein N‐terminus were set as variable modifications. The localization of the post‐translational modification sites within the peptides was performed with the tool ptmRS, based on the tool phosphoRS (Taus *et al*, 2011). Identifications were filtered again to 1 % FDR on protein and PSM level; additionally, an Amanda score cutoff of at least 150 was applied. Peptides were subjected to label‐free quantification using IMP‐apQuant (Doblmann *et al*, 2018). Proteins were quantified by summing unique and razor peptides or only unique peptides and applying intensity‐based absolute quantification (iBAQ; Schwanhäusser *et al*, 2011). FLAG‐Gal4‐DBD‐RD bait proteins were filtered to be identified by a minimum of 2 PSMs in at least one sample. All other proteins were filtered to be identified by a minimum of three quantified peptides in at least one sample. Protein‐abundances‐normalization was done using sum normalization. Differential abundance protein analysis between each RD group and Gal4‐DBD constructs was performed using limma (Smyth, 2004), considering all replicates. The results of the differential abundance analysis can be found in Dataset EV9.

#### RNAi‐mediated depletion of co‐repressors

For RNAi‐mediated depletion of CoRs, two distinct long dsRNAs targeting each CoR, without off‐target effects, were selected from UP‐TORR (Hu *et al*, 2013; https://www.flyrnai.org/up‐torr/). As a negative control, we used a dsRNA targeting the Renilla Luciferase, which is not expressed in *Drosophila* S2 cells (sequences in Dataset EV15). Primers including the T7 promoter sequence (TAATACGACTCACTATAGGG) in their overhangs (Dataset EV15) were used to amplify these dsRNA‐complementary sequences from *Drosophila* genomic DNA with the Q5® Hot Start High‐Fidelity 2X Master Mix (NEB). The PCR product was precipitated in 1 volume isopropanol and 1/10 3 M sodium acetate for 5 min at room temperature, followed by centrifugation for 20 min at 18,000 *g* at 4°C, a wash with 70% ethanol and resuspension in nuclease‐free water. Subsequently, the fragments were transcribed with the T7 RNA Polymerase (Promega) at 37°C overnight. After DNase digest (Turbo DNase I Ambion) at 37°C for 1 h, the RNA was purified in a phenol‐chloroform extraction. Samples were treated with 1 volume of Acid‐Phenol‐Chloroform (Roti‐Aqua‐P/C/I) for 5 min at room temperature followed by centrifugation and recovery of the aqueous phase. The RNA was precipitated by adding 2.5 volumes 100% ethanol and 1/10 volume 3 M sodium acetate and incubation at ‐20°C for 30 min. After centrifugation and washing with 70% ethanol, the RNA was purified using the Invitrogen MEGAclear Transcription Clean‐Up Kit.

*Drosophila* zfh1‐DSCP reporter cells were transfected with the Gal4‐DBD‐RD plasmid or the Gal4‐DBD control plasmid according to *RD validations*, using 5 μg instead of 2.5 μg plasmid for 25 × 10^6^ cells. Sixteen hours after transfection, cells were harvested, washed twice in PBS, and resuspended in serum‐free medium (ExpressFive SFM [Invitrogen], 16 mM Glutamine [Gibco]). For each condition, 0.75 × 10^6^ cells in 500 μl serum‐free medium were seeded into 12‐well tissue culture plates; 20 μg dsRNA was added and incubated for 1 h at 27°C, before adding 1 ml full medium (ExpressFive SFM Invitrogen, 16 mM Glutamine, 10% FBS [Sigma‐Aldrich], and 1% penicillin–streptomycin [Gibco]) to each well. Three days after dsRNA treatment, cells were submitted to flow cytometry analysis as in *RD validations*. The fold change (FC) repression was determined as the ratio of the median GFP signal of transfected cells compared between cells expressing the Gal4‐DBD control and cells expressing the Gal4‐DBD‐RD, both treated with the same dsRNA.

The log2 FC repression values of the “noRNA” condition and the different dsRNA treatments were compared for three independent replicates with two‐tailed, paired Student's *t*‐tests (*P* ≤ 0.05; FC > 1 for validated). FC repression values and *P*‐values can be found in Dataset EV15.

Reverse transcription quantitative PCR (RT–qPCR) was performed to assess the depletion of the endogenous CoRs. Three days after treatment of nontransfected reporter cells as described above, cells were harvested, followed by total RNA isolation with the Quiagen RNeasy Mini Kit and DNA digest with Ambion Turbo DNaseI. The RNA was reverse transcribed using Oligo(dt)20 primer (Invitrogen, 18418020) and SuperScript III Reverse Transcriptase (Invitrogen). qPCR with three technical replicates per condition was performed with the Promega GoTaq qPCR Master Mix (qPCR primers in Dataset EV15). qPCR was analyzed using the Delta–Delta Ct Method (Livak & Schmittgen, 2001). Conditions with primers targeting the rps12 gene were used as a housekeeping gene control. In brief, the following equations were used: DeltaCt = mean Ct CoR primers – mean Ct rps12 primers; DeltaDeltaCt = DeltaCt – RenillaDeltaCt; FC = 2^(‐ DeltaDeltaCt).

#### Sequence alignments for RD‐containing repressors

Orthologs of *Drosophila* proteins harboring RDs with specific repressive motifs were detected in the NCBI protein or UniProt reference database, based on NCBI blast searches applying significant e‐values (<0.001) and considering reciprocal best hits (Altschul, 1997; Agarwala *et al*, 2018; Bateman *et al*, 2021). In addition to fruit fly (*Drosophila melanogaster*), six other species were selected for a long evolutionary distance and presence in all four motifs, namely southern house mosquito (*Culex quinquefasciatus*), American lobster (*Homarus americanus*), a tardigrade (*Ramazzottius varieornatus*), a bivalve (*Mytilus coruscus*), zebrafish (*Danio rerio*), and human (*Homo sapiens*). Alignments were performed with mafft (‐linsi, v7.427; Katoh & Toh, 2008) and visualization in Jalview (ClustalX coloring scheme; Waterhouse *et al*, 2009). Accessions and gene names are given in Dataset EV16. Gene names are according to Uniprot or NCBI nomenclature.

#### Analysis of motif conservation in fly and human proteins

To measure the conservation of each amino acid of *Drosophila melanogaster* and human transcription‐related proteins, we first identified groups of orthologous proteins (= orthogroups) across a range of species from either the Panarthropoda clade for comparison with *Drosophila* or the vertebrate clade for comparison to human with Orthofinder (Emms & Kelly, 2019) and used these groups for multiple sequence alignments.

Sixty‐four species of the Panarthropoda clade and 40 species from the vertebrate clade were selected from the UniProt reference proteomes (Bateman *et al*, 2021; Dataset EV17). Orthogroups were detected using OrthoFinder for the clades individually, with diamond ultrasensitive mode and an e‐value threshold of 0.001, version 2.5.4 (Emms & Kelly, 2019).

In the Panarthropoda set, 590 orthogroups had all species present and were used to infer a rooted species tree with STAG and to build hierarchical orthogroups (HOGs) in OrthoFinder (preprint: Emms & Kelly, 2018). We used the list of 1,133 transcription‐related proteins from *Drosophila melanogaster* (Dataset EV1). We only processed orthogroups containing equal or less than 150 entries and 1,024 orthogroups of the root node (N0, Panarthropoda). 1,072 of the *Drosophila* transcription‐related proteins fulfilled these criteria. Four more orthogroups (9 transcription factors) were derived from the N2 node (insects) and one more orthogroup from the N6 node (Endopterygota).

In the vertebrates set, 3,775 orthogroups contained all species and were used for the species tree. The human transcription factor list contained 2,754 IDs (Dataset EV10) that were mapped to 2,740 UniProt entries. 2,259 orthogroups (2,470 UniProt IDs) were retrieved from the root N0 (vertebrates) node, 29 orthogroups (116 IDs) with the N6 node (tetrapods), and 5 orthogroups (38 IDs) with the N14 node (mammals).

All orthogroup sequences were aligned with mafft (‐linsi mode, v7.427; Katoh & Toh, 2008) and the sequence conservation score calculated with AAcon (KARLIN method, results normalized with values between 0 and 1; see Golicz *et al*, 2018).

We next mapped the positions of all instances of the five main SLiMs (EH1, PLKKR, HKKF, PxDLS, and AAxxL) within the protein sequence of *Drosophila* and human transcription‐related factors using FIMO (as described in section *MEME and FIMO peptide motif searches among RD‐seq hits*, FIMO *P* < 0.0001). We quantified the conservation of each instance as the averaged conservation of its amino acids and compared it with the average conservation of the flanking amino acids (sequences with same total length as the motifs up‐ and downstream of motif instance; Fig 4C and D). We further analyzed the conservation of extended regions around the 5 motifs (±100 AA from the center of each motif) for all motif instances (from FIMO with *P* < 0.0001) or only for high confidence hits (FIMO with *P* < 0.00005) and show the median conservation per position across all instances, centered on the motifs (Fig EV4C and D). Each dot in the panels represents the median conservation for the particular position centered around the motif.

#### FIMO searches among human transcription‐related proteins

In order to predict RDs in human proteins, we used minimal MEME motifs of the PxDLS, PLKKR, EH1, HKKF, and AAxxL motifs found in fly as input for FIMO searches (v.5.4.1; Grant *et al*, 2011) among human transcription‐related genes (Vaquerizas *et al*, 2009; Lambert *et al*, 2018; Dataset EV10). Some motifs were detected in multiple RD sets, for example, the EH1 motif was found in MEME searches with zfh1, global, and all RDs (see Fig 2C and Material and Methods *MEME and FIMO peptide motif searches among RD‐seq hits*). For the FIMO searches among human proteins, we selected one motif per group: Motif 1 AAxxL—ent1, Motif 2 PxDLS—all, Motif 3 HKKF—global, Motif 4 PLKKR—global, and Motif 5 EH1—global (see Dataset EV6 for minimal MEME motifs). These are the same minimal MEME motifs that were used to determine motif instances among RDs found in fly. We used a stringent (*P* < 0.0001) cutoff to determine the prevalence of these peptide motifs among human transcription‐related genes (Vaquerizas *et al*, 2009; Lambert *et al*, 2018; Dataset EV10). The results of the FIMO searches among human transcription‐related genes can be found in Dataset EV11.

## Author contributions

**Loni Klaus:** Conceptualization; formal analysis; funding acquisition; validation; investigation; visualization; methodology; writing—original draft; writing—review and editing. **Bernardo P de Almeida:** Software; formal analysis; visualization; writing—review and editing. **Anna Vlasova:** Data curation; software; formal analysis. **Filip Nemčko:** Methodology; writing—review and editing. **Alexander Schleiffer:** Formal analysis. **Katharina Bergauer:** Resources; investigation. **Lorena Hofbauer:** Investigation; writing—review and editing. **Martina Rath:** Investigation. **Alexander Stark:** Conceptualization; supervision; funding acquisition; methodology; writing—review and editing.

## Disclosure and competing interests statement

The authors declare that they have no conflict of interest.

## Supporting information



Expanded View Figures PDF

Dataset EV1

Dataset EV2

Dataset EV3

Dataset EV4

Dataset EV5

Dataset EV6

Dataset EV7

Dataset EV8

Dataset EV9

Dataset EV10

Dataset EV11

Dataset EV12

Dataset EV13

Dataset EV14

Dataset EV15

Dataset EV16

Dataset EV17

Dataset EV18

Source Data for Expanded View

PDF+

Source Data for Figure 1

Source Data for Figure 2

Source Data for Figure 3

## Data Availability

Datasets produced in this study are available in the following databases:
RD‐seq raw sequencing data: Gene Expression Omnibus (GEO) GSE207374 https://www.ncbi.nlm.nih.gov/geo/query/acc.cgi?acc=GSE207374
Mass spectrometry raw data, MobiDB‐lite search data, FIMO motif instances for all tiles, sequences used to make the AA sequence logos, *Dmel* and human protein conservation scores: zenodo 6786955 https://doi.org/10.5281/zenodo.6786955
Tracks with read coverage and RD regions of RD‐seq screens: UCSC Genome Browser https://genome.ucsc.edu/s/bernardo.almeida/RDseq_manuscript
