# Supplementary material for: Systematic identification and characterization of repressive domains in Drosophila transcription factors
Source: EMBO J. 2022 Dec 22;42(3):e112100. doi: 10.15252/embj.2022112100 (PMC9890238; doi:10.15252/embj.2022112100)
Supplement: Supplementary file 1 — Expanded View Figures PDF [file EMBJ-42-e112100-s013.pdf]

Expanded View Figures

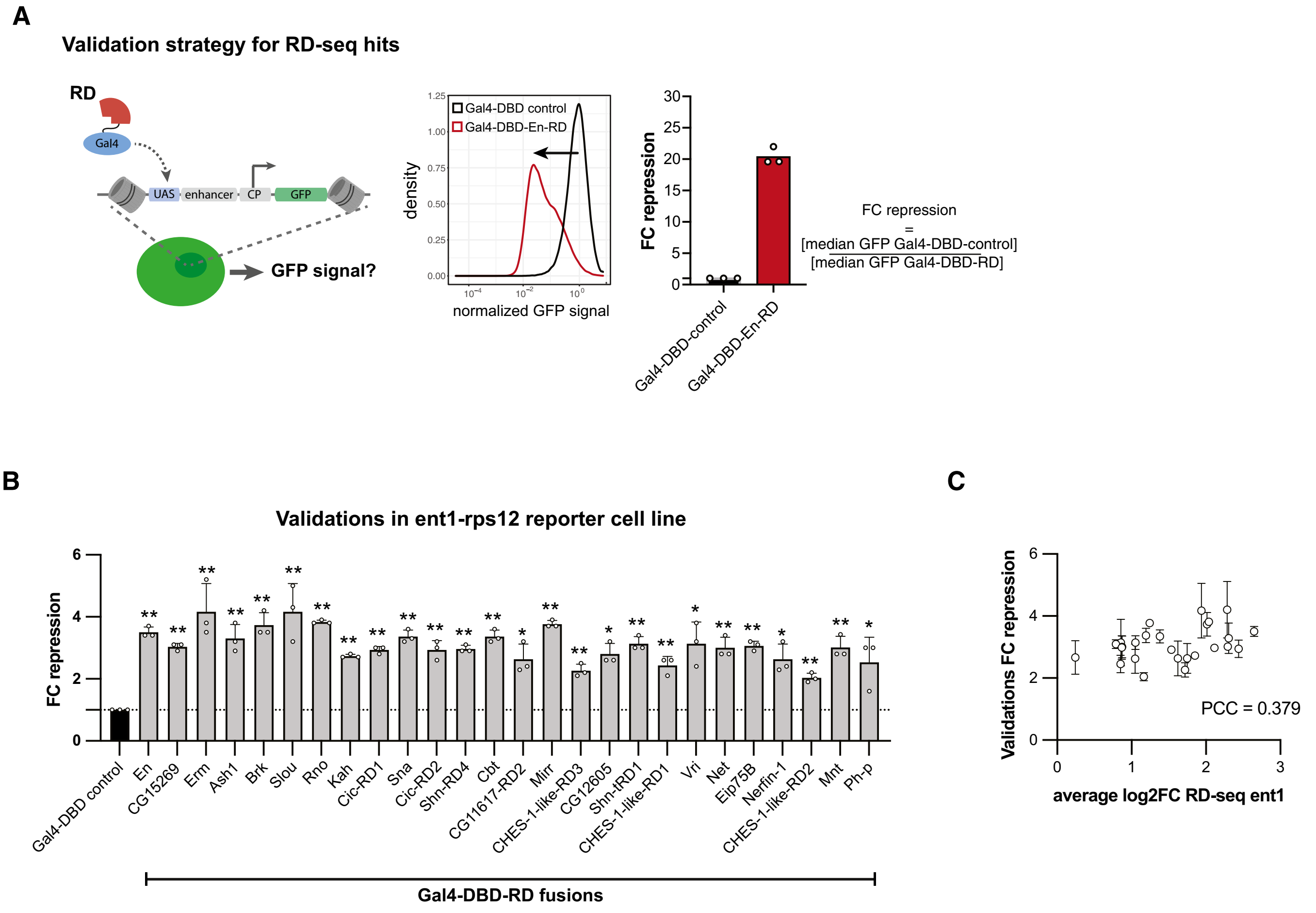

Figure EV1. Validations of RD-seq hits.

A Validation strategy for RD-seq hits. The reporter cell line is transfected with either the Gal4-DBD-fused RD or a Gal4-DBD construct as a control, followed by assessment of the GFP signal by flow cytometry (left). The density distribution shows the normalized GFP signal of cells expressing either the Gal4-DBD control or the Gal4-DBD-En-RD construct (middle). The fold change (FC) repression is calculated as the ratio of the median GFP intensity of the Gal4-DBD control and the Gal4-DBD-RD condition (mean FC repression and individual values of 3 biological replicates; right).

B Validations of RD-seq hits in comparison with the Gal4-DBD control in the ent1-rps12 reporter cell line (mean FC repression and individual values of 3 biological replicates, error bars: s.d., two-tailed, paired Student's *t*-tests comparing to Gal4-DBD control with * for $P \leq 0.05$, ** for $P \leq 0.01$).

C Comparison between validation FC repression values and average log2 FC in RD-seq for each RD region in the ent1-rps12 reporter cell line (PCC: Pearson correlation coefficient; error bars: s.d. of 3 biological replicates of validations).

Source data are available online for this figure.

Figure EV2. RD and DBD positioning and expression of mutated RDs.

A Positioning of RDs and DBDs. Density distribution of the position of the center of the 50 AA RD or the DBD regions within their full-length protein. Positions are scaled over the length of the respective protein sequences.

B Positioning of RDs with distinct motifs from MEME *de novo* motif searches. Shown are frequency histograms of the position of the center of the 50 AA RD within its full-length protein for all RDs containing each motif type. Positions are scaled over the length of the respective protein sequences.

C Western blots for FLAG-Gal4-DBD-tagged wild-type and motif mutant RDs expressed in the zfh1-DSCP reporter cell line (anti-Tubulin as loading control).

Source data are available online for this figure.

© 2022 The Authors

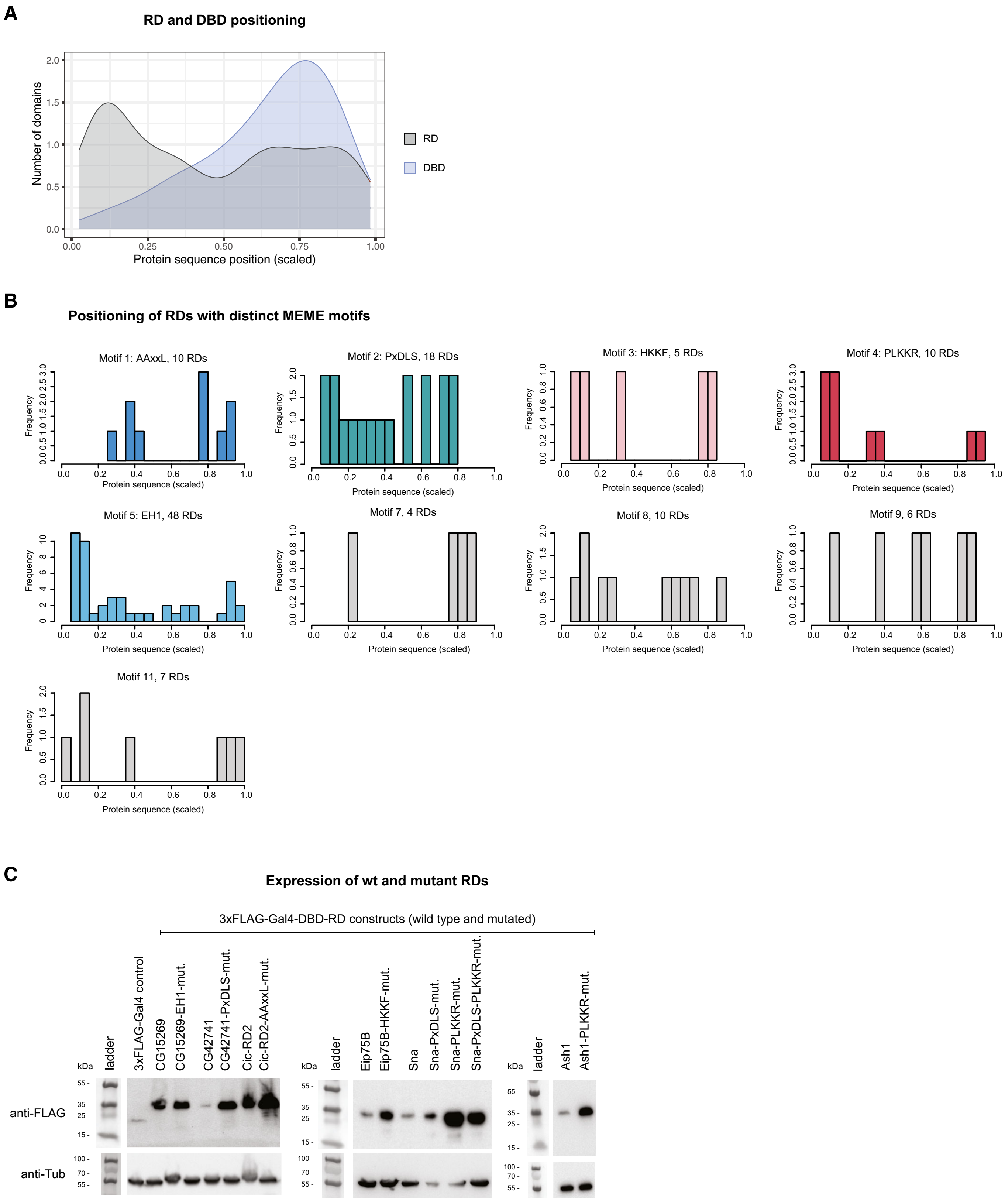

Figure EV2.

 © 2022 The Authors

Figure EV3. RNAi-mediated co-repressor depletion and repressors with multiple RDs and repressive motifs.

A Assessment of depletion of CoR mRNA with RNAi through reverse transcription quantitative PCR (RT–qPCR). Each CoR was targeted with 2 different dsRNA constructs (x-axis). A dsRNA targeting Renilla was used as a negative control. Shown is the fold change (FC) relative to the control condition calculated with the Delta–Delta Ct Method (3 technical replicates, error bars: s.d.).

B, C Validations of RDs upon RNAi-mediated depletion of CoRs in the zfh1-DSCP reporter cell line (mean FC repression values of 3 biological replicates, error bars: s.d., two-tailed, paired Student's *t*-tests comparing to noRNA control with * for $P \leq 0.05$, ** for $P \leq 0.01$, or ns for $P > 0.05$). Each CoR was targeted for depletion with 2 different dsRNA constructs (x-axis). The repressive motif contained in the tested RD is indicated above the panels.

D Examples of repressors with multiple RDs and RDs with multiple repressive motifs. Shown are RD sequences, presence of repressive motifs, their associated interacting CoRs and literature references.

E Validation of wild-type and mutant Sna-RD in the zfh1-DSCP reporter cell line (mean FC repression of 3 biological replicates, error bars: s.d., two-tailed, paired Student's *t*-tests comparing RD wt vs. mutant with * for $P \leq 0.05$ or ns for $P > 0.05$).

F Validation of Sna-RD upon RNAi-mediated depletion of CtBP in the zfh1-DSCP reporter cell line (mean FC repression values of 3 biological replicates, error bars: s.d., two-tailed paired Student's *t*-tests comparing to noRNA control with * for $P \leq 0.05$, ** for $P \leq 0.01$, or ns for $P > 0.05$). CtBP was targeted for depletion with 2 different dsRNA constructs (x-axis).

Source data are available online for this figure.

A

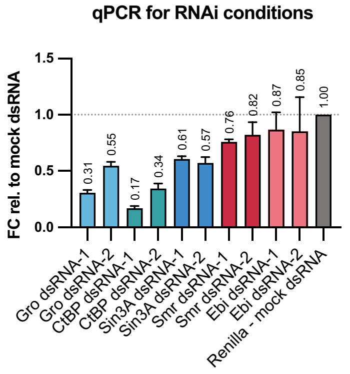

B

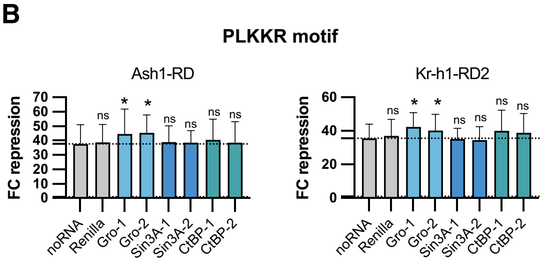

C

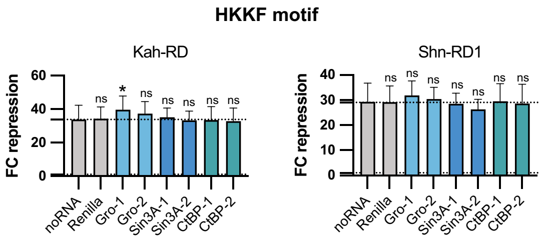

D

RD name	RD AA sequence	motif	associated CoR	comment	doi
Shn-RD1	TVNATPATDEAPLGDRSNISRYLHKKFKRLASTTEVDSWSATNGGGALQA	HKKF	Smr	Smr interaction supported by Cai *et al.* 2009	10.1016/j.bbagrm.2009.01.001
Shn-RD2	PLGSPAAGTLPPTTADNHHSATAQHRQSIDYKPYKPKFHNASLYSCSSKE	NA	NA	Smr according to Cai *et al.* 2009	10.1016/j.bbagrm.2009.01.001
Shn-RD3	PSPGPLLGKTPLVDYAQQSTPRKAQDSVVITKMHEDRQFVIEAQPAKRIK	NA	NA	Groucho according to Cai *et al.* 2009	10.1016/j.bbagrm.2009.01.001
Shn-RD4	DESKSRQKEHEAARGLLSLSMTPPIPQSVSPYPQLQDTPLPAASPANSIG	AAxxL	Sin3A	Sin3A interaction supported by Cai *et al.* 2009	10.1016/j.bbagrm.2009.01.001
CHES-1-like-RD1	SPAGGGTIYKAASQALSSPTTSSSSSSNTTTHHPHKKYLREKMHVQMDHS	HKKF	Smr	CoR assignment because of HKKF motif presence	this study
CHES-1-like-RD2	SDGINFARLARDCGADSIDDVHAAAAMLYLKHGPKIYSEPFQNGSGPVIT	AAxxL	Sin3A	human ortholog FOXN3 known to interact with Sin3A, Li *et al.* 2017	10.1172/JCI94233
Sna-RD	ANYKSCPLKKRPIVFVEERLPQTEALALTKDSQFAQDQPQDLSLKRGRDE	PLKKR and PxDLS	Smr and CtBP	CoR interactions supported by Qi *et al.* 2008	10.1038/emboj.2008.26
Wor-RD	MDKLKYSRCPLKKRPIMVEESSPEDHLSHDEGPVDLSVASAAVPMEPHWM	PLKKR and PxDLS	Smr and CtBP	CoR interactions supported for ortholog Sna by Qi *et al.* 2008	10.1038/emboj.2008.26

E

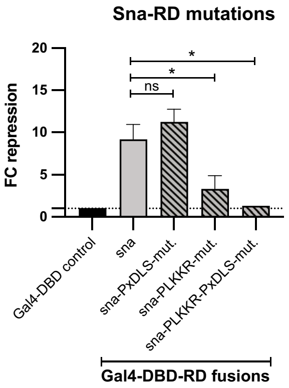

F

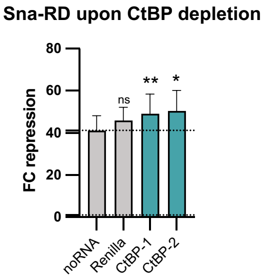

Figure EV3.

 © 2022 The Authors

Figure EV4. Sequence alignments of RDs and conservation of regions around repressive motifs.

A, B Sequence alignments for a region of *Dmel* TFs (A) Eip93F containing the PxDLS motif and (B) Vri containing the PLKKR motif and the respective orthologous sequences from different species. Numbers on the left and right indicate the range of amino acids shown referring to the full-length proteins. Consensus sequences are indicated at the bottom.

C, D Metaplots showing the median conservation scores of positions within repressive motifs (highlighted in color) and ± 100 flanking amino acids for motif instances among fly transcription-related proteins resulting from standard (C) and stringent (D) FIMO searches. See also Fig 4C and D for summary analyses.

© 2022 The Authors

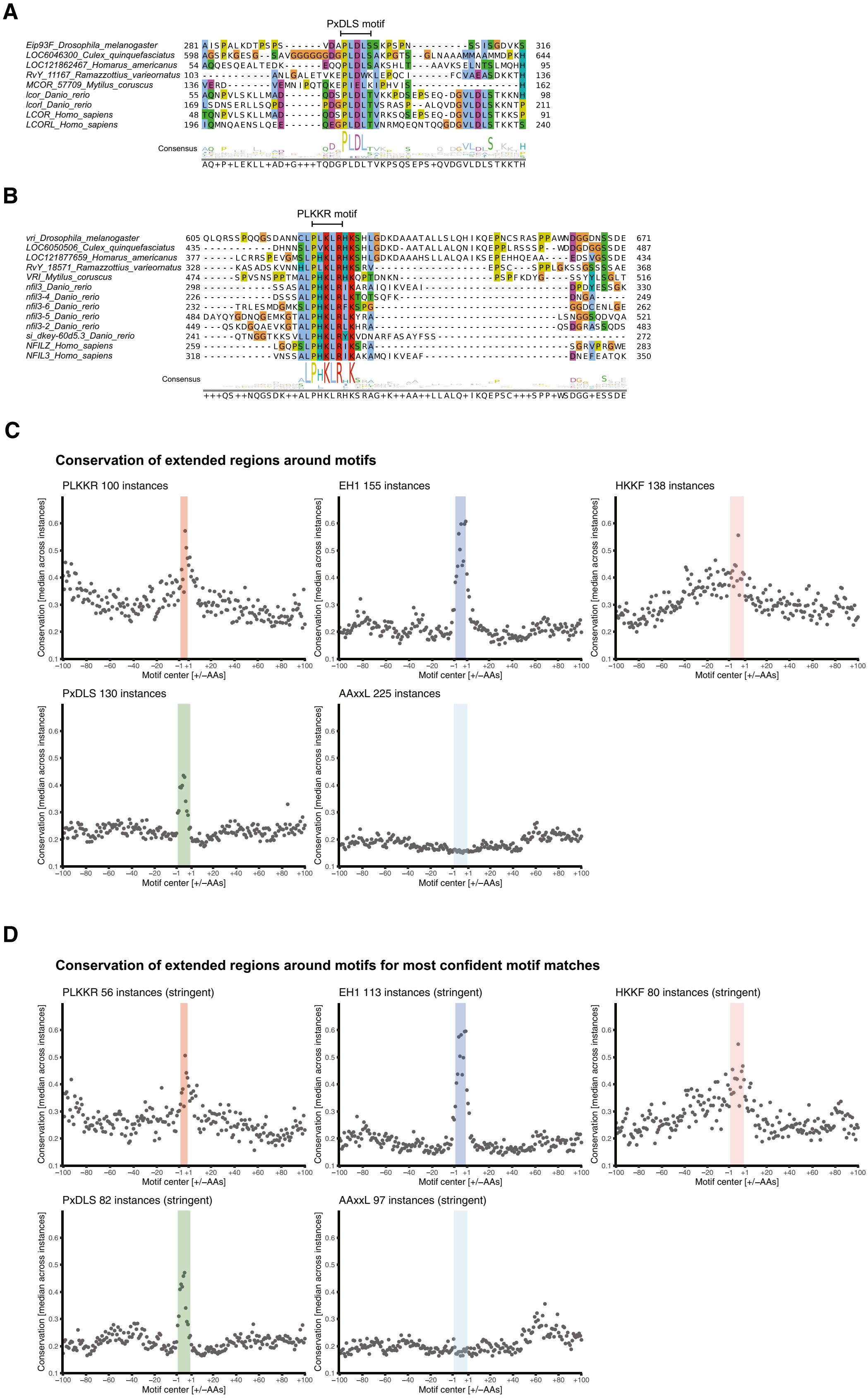

Figure EV4.

 © 2022 The Authors

Figure EV5. Analysis of non-RD tiles with repressive motifs.

A Average strength of tiles in RD-seq screens with the zfh1-DSCP reporter cell line for tiles in or outside RDs that have or do not have one of the repressive motifs indicated on the top. N of instances for RD&motif/no-RD&motif/no-RD&no-motif: EH1 341/1124/204131, PLKKR 50/1109/204146, PxDLS 136/1303/203952, AAxxL 51/2327/202928, HKKF 33/1173/204082. The box plots mark the median, upper and lower quartiles and 1.5× interquartile range (whiskers). Differences between the groups of tiles were assessed with two-sided Wilcoxon rank-sum tests, and *P*-values are shown within the plots.

B–F Amino acid sequence logos for instances of repressive motifs within and outside RDs. For motif instances in non-RD proteins, all instances and a number of instances matched to the number of instances within RDs are shown separately. Positions of the core motifs are indicated below the logos.

© 2022 The Authors

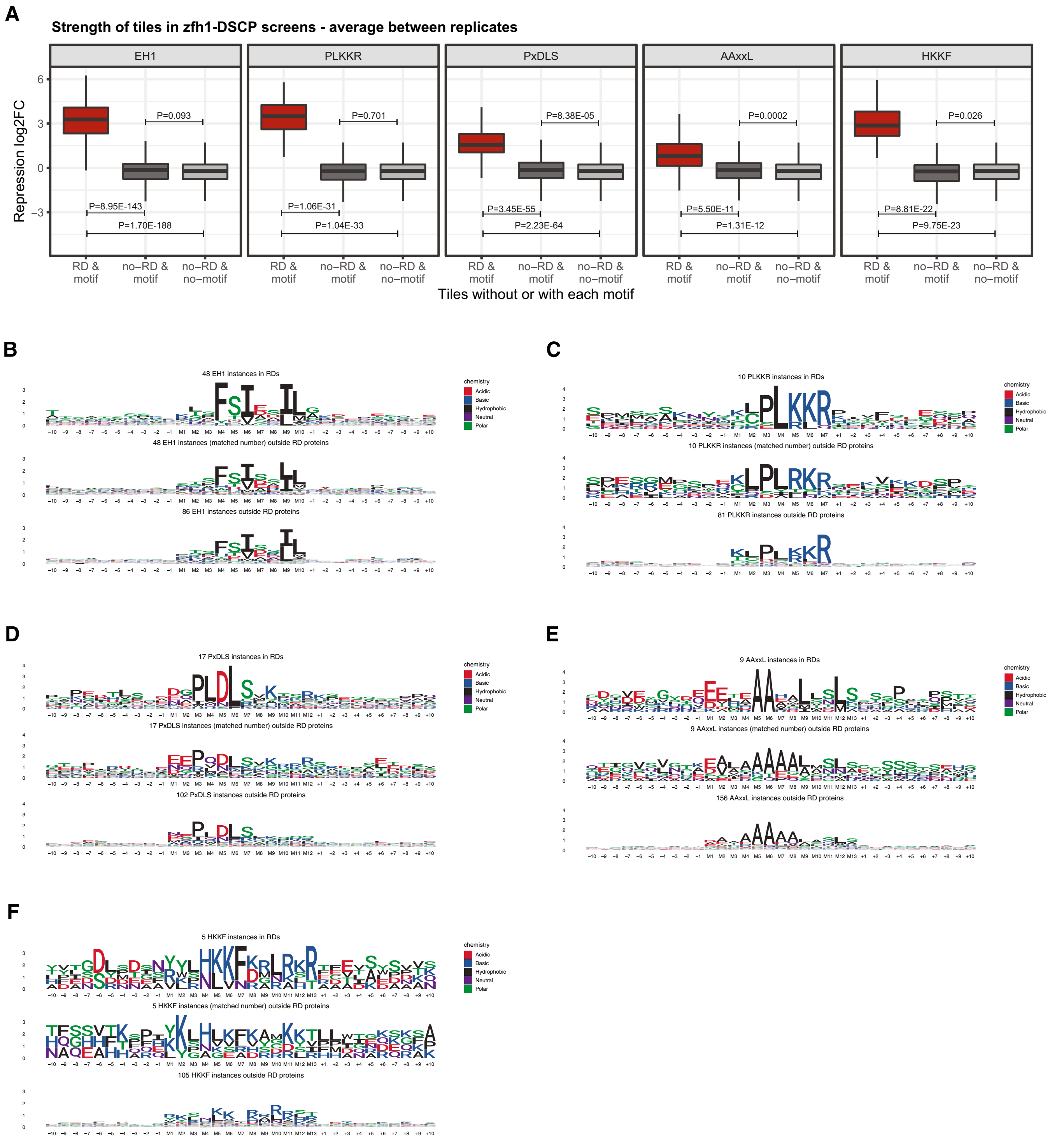

Figure EV5.

© 2022 The Authors
