# Supplementary material for: Systematic identification and characterization of repressive domains in Drosophila transcription factors
Source: EMBO J. 2022 Dec 22;42(3):e112100. doi: 10.15252/embj.2022112100 (PMC9890238; doi:10.15252/embj.2022112100)
Supplement: Supplementary file 20 — Source Data for Expanded View [file EMBJ-42-e112100-s016.zip › FigureEV2/Source_Data_FigEV2C/readme.docx]

Fig EV2C -Source Data

All Western Blots:

- Ladder: PageRuler™ Plus Prestained Protein Ladder, 10 to 250 kDa #26619
- Anti-FLAG antibody: Sigma F1804-200UG
- Anti-Tubulin antibody: Abcam ab18251
- All blots were first imaged for anti-FLAG and afterwards imaged for anti-Tubulin without stripping. Therefore in some cases bands coming from the anti-FLAG imaging are still visible in the anti-Tubulin blots.

Blot1:

- 3xFLAG-Gal4-DBD control
- CG15269-RD
- CG15269-RD-EH1-mut
- CG42741-RD
- CG42741-RD-PxDLS-mut
- Cic-RD2
- Cic-RD2-AAxxL-mut

Blot2:

- Eip75B-RD
- Eip75B-RD-HKKF-mut
- Sna-RD
- Sna-RD-PxDLS-mut
- Sna-RD-PLKKR-mut
- Sna-RD-PxDLS-PLKKR-mut

Blot3:

- Ash1-RD
- Ash1-RD-PLKKR-mut
