# Supplementary figures and images for: Systematic identification and characterization of repressive domains in Drosophila transcription factors
Source: EMBO J. 2022 Dec 22;42(3):e112100. doi: 10.15252/embj.2022112100 (PMC9890238; doi:10.15252/embj.2022112100)

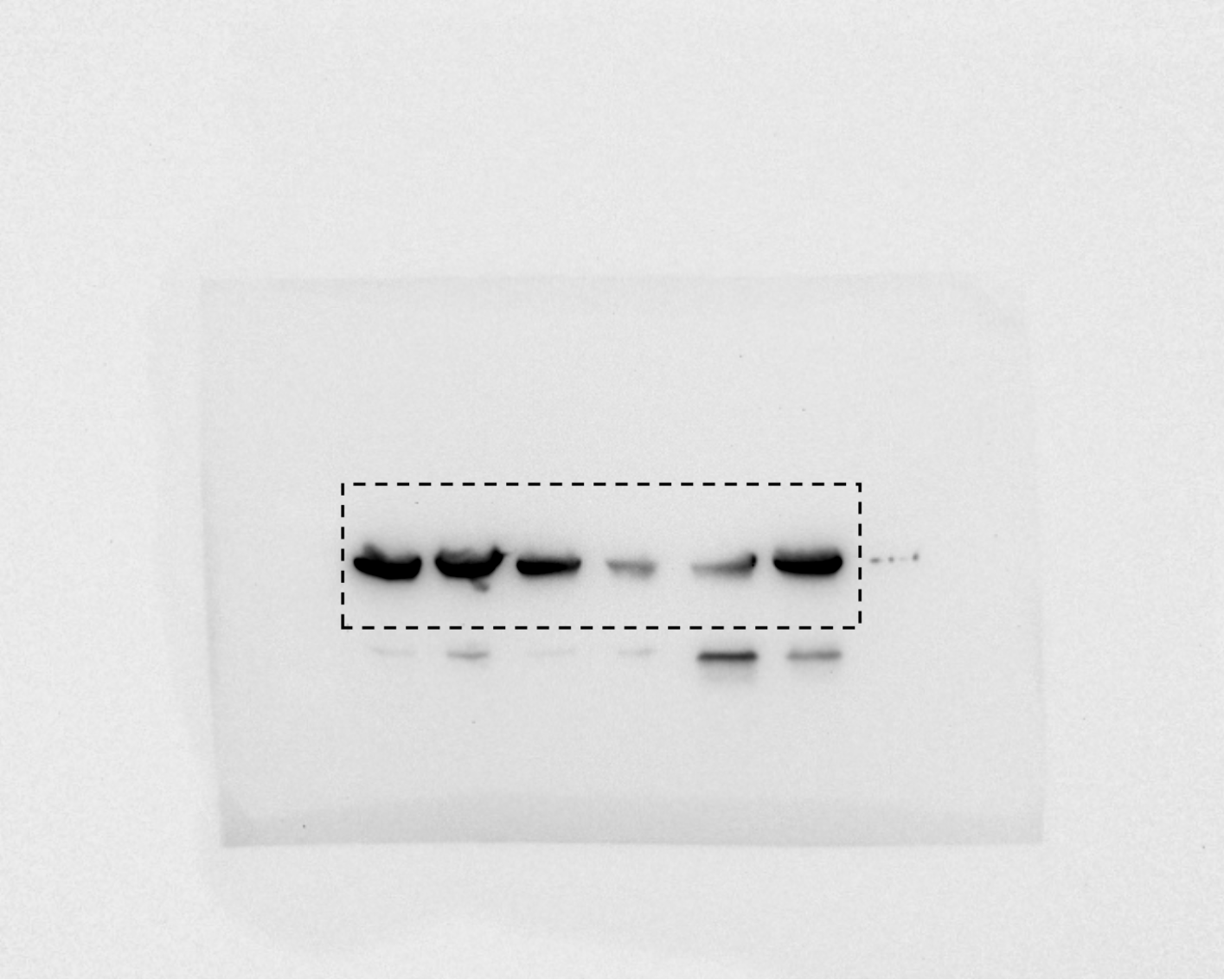

Supplement: Supplementary file 20 — Source Data for Expanded View [file EMBJ-42-e112100-s016.zip › FigureEV2/Source_Data_FigEV2C/Blot2/Blot2_anti-Tub.tif]

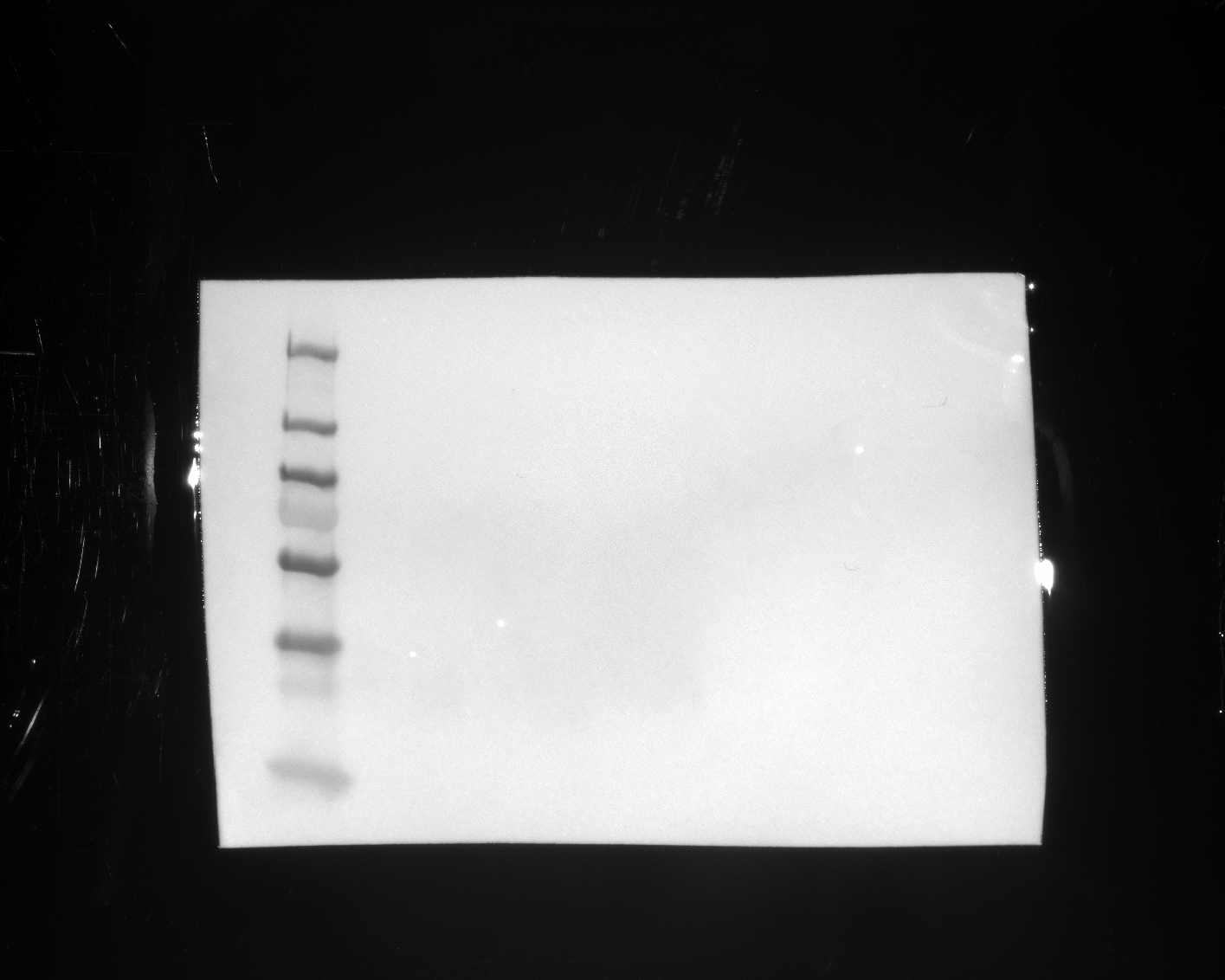

Supplement: Supplementary file 20 — Source Data for Expanded View [file EMBJ-42-e112100-s016.zip › FigureEV2/Source_Data_FigEV2C/Blot2/Blot2_anti-Tub_ladder.tif]

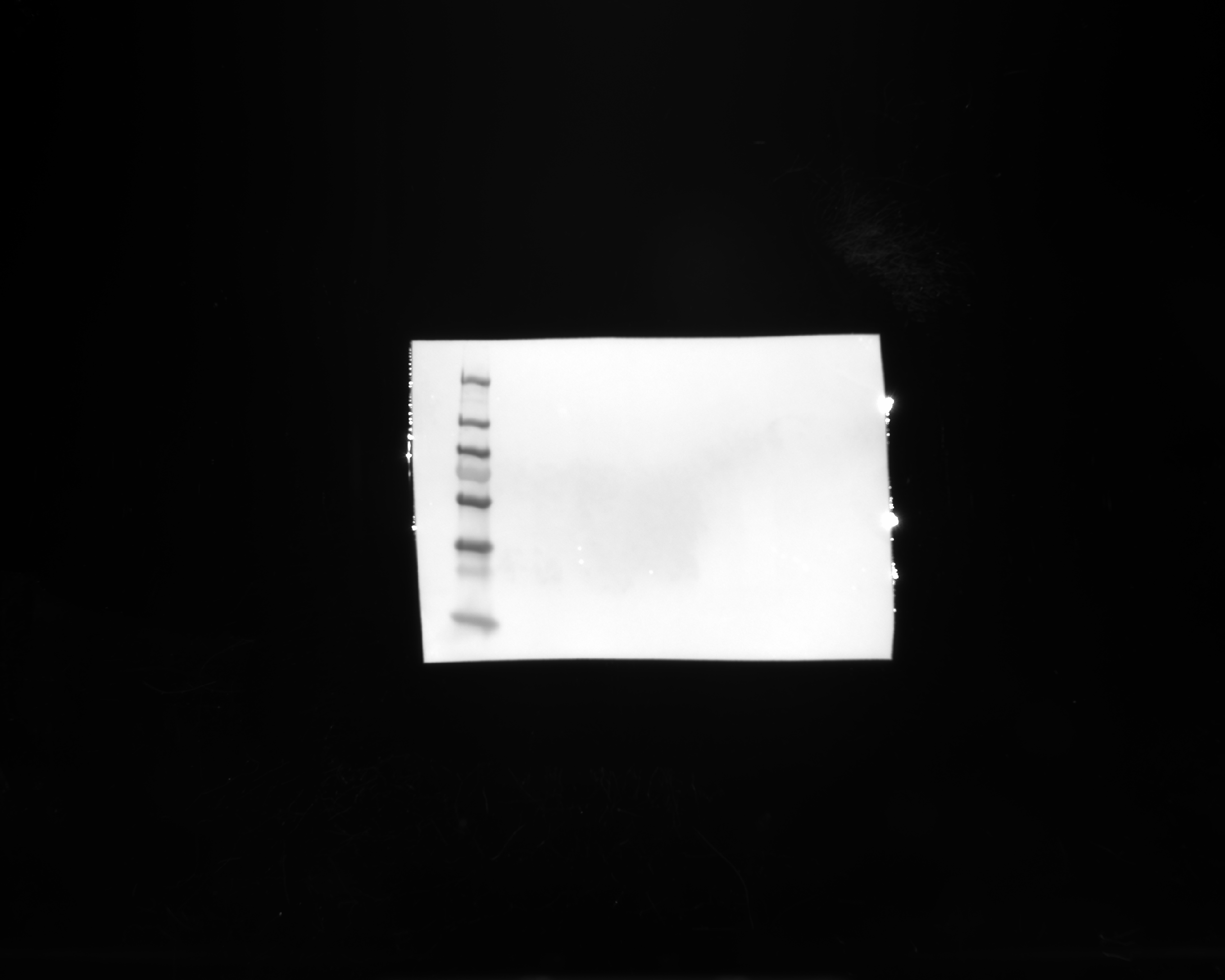

Supplement: Supplementary file 20 — Source Data for Expanded View [file EMBJ-42-e112100-s016.zip › FigureEV2/Source_Data_FigEV2C/Blot2/Blot2_anti-FLAG_ladder.tif]

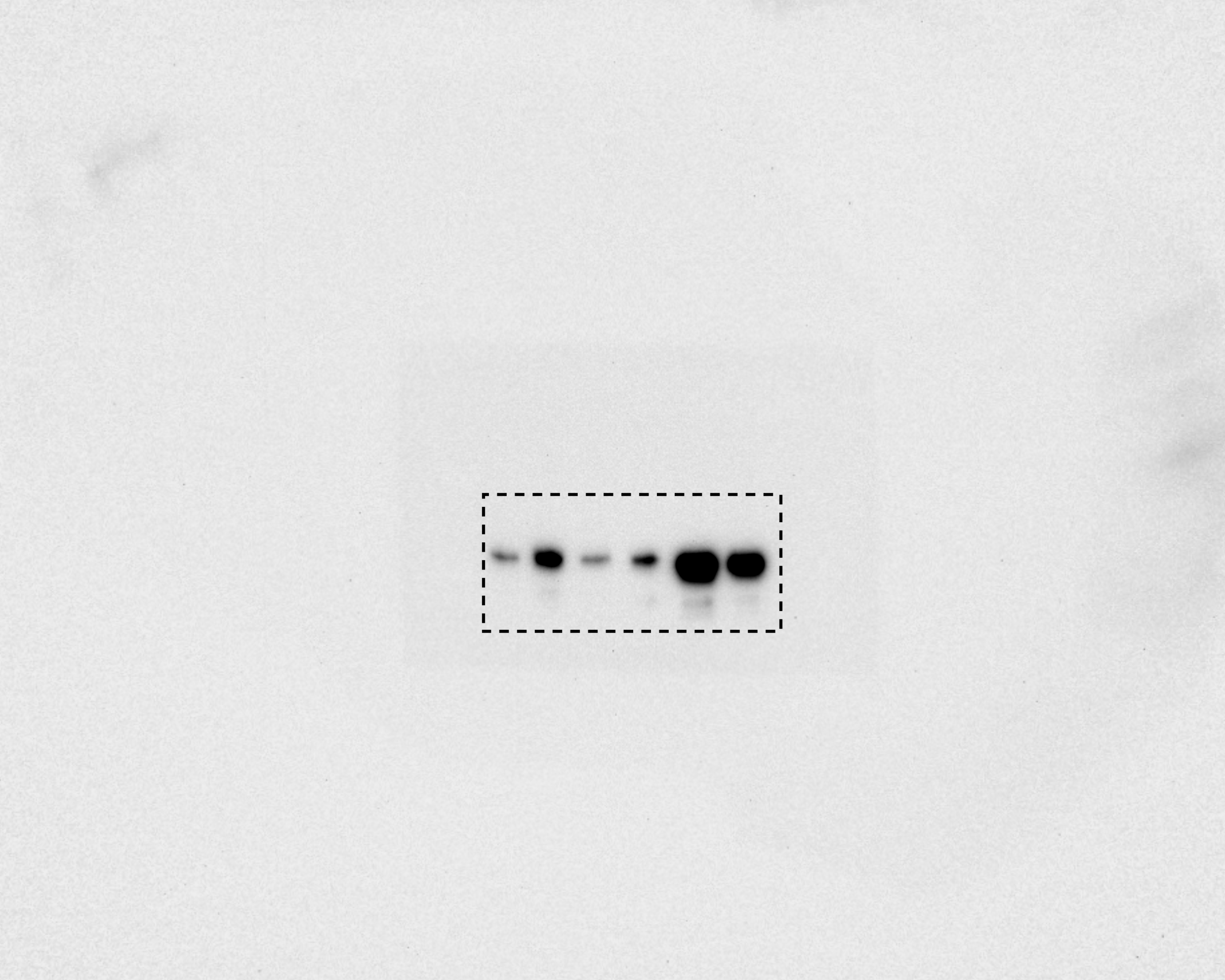

Supplement: Supplementary file 20 — Source Data for Expanded View [file EMBJ-42-e112100-s016.zip › FigureEV2/Source_Data_FigEV2C/Blot2/Blot2_anti-FLAG.tif]

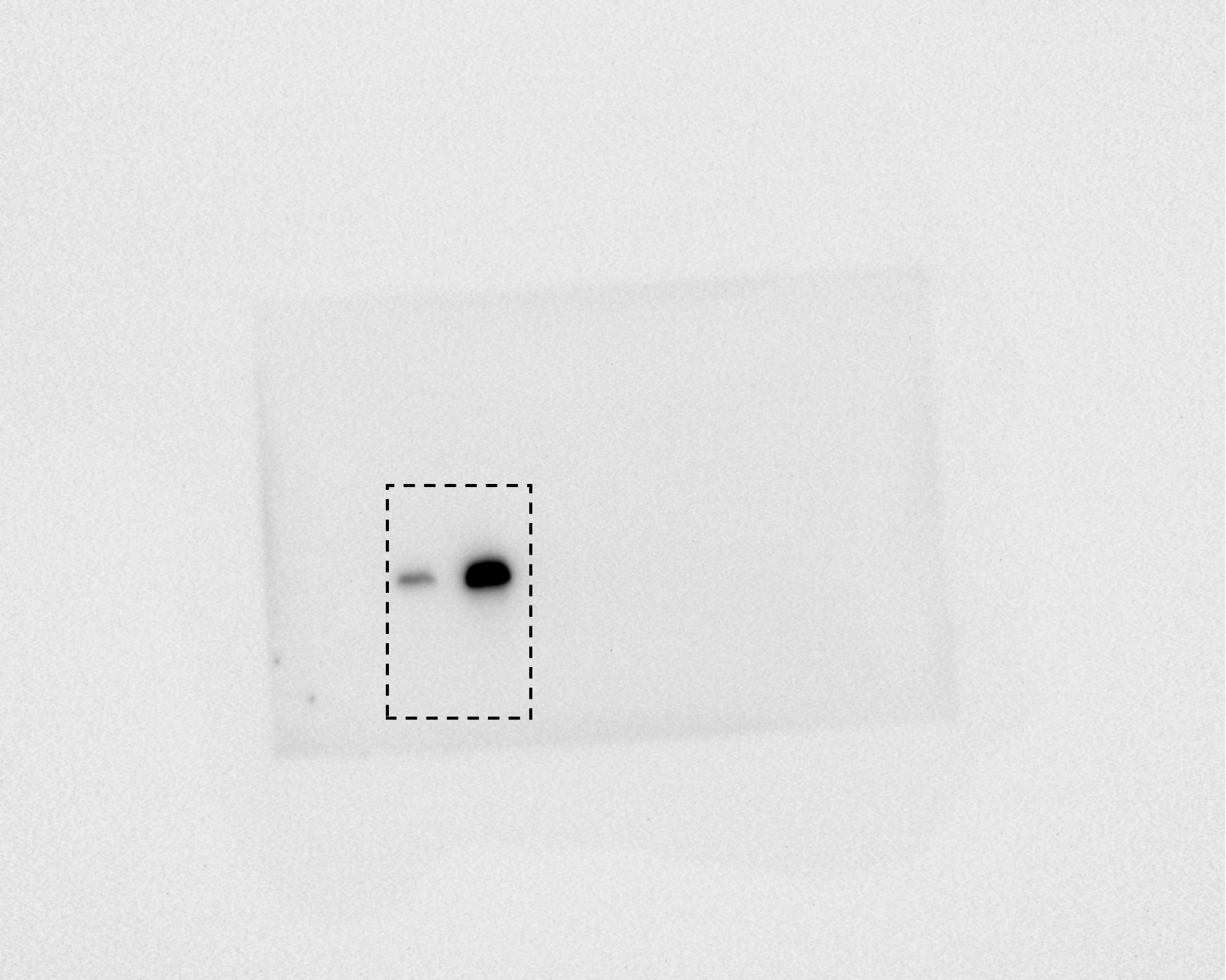

Supplement: Supplementary file 20 — Source Data for Expanded View [file EMBJ-42-e112100-s016.zip › FigureEV2/Source_Data_FigEV2C/Blot3/Blot3_anti-FLAG.tif]

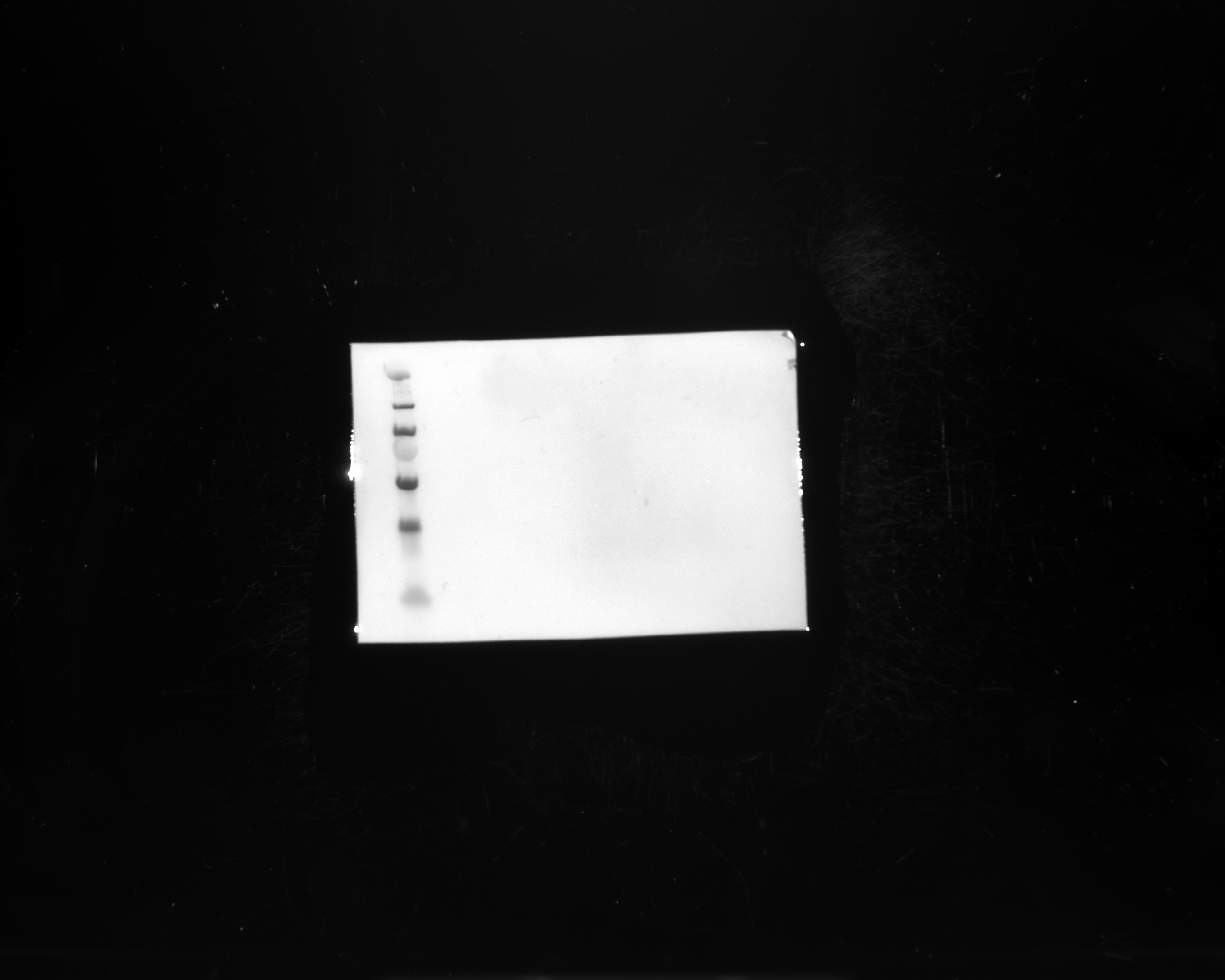

Supplement: Supplementary file 20 — Source Data for Expanded View [file EMBJ-42-e112100-s016.zip › FigureEV2/Source_Data_FigEV2C/Blot3/Blot3_anti-Tub_ladder.tif]

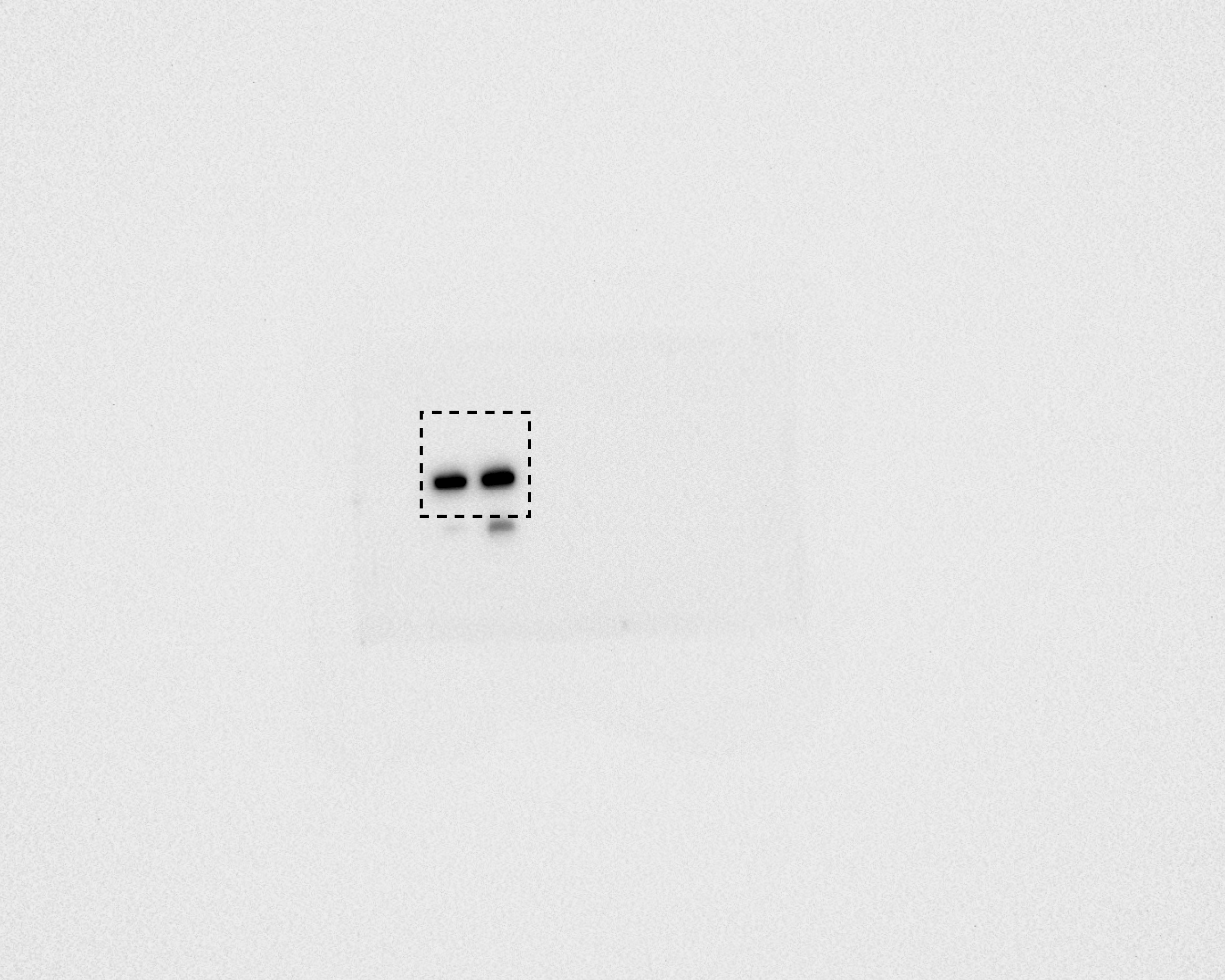

Supplement: Supplementary file 20 — Source Data for Expanded View [file EMBJ-42-e112100-s016.zip › FigureEV2/Source_Data_FigEV2C/Blot3/Blot3_anti-Tub.tif]

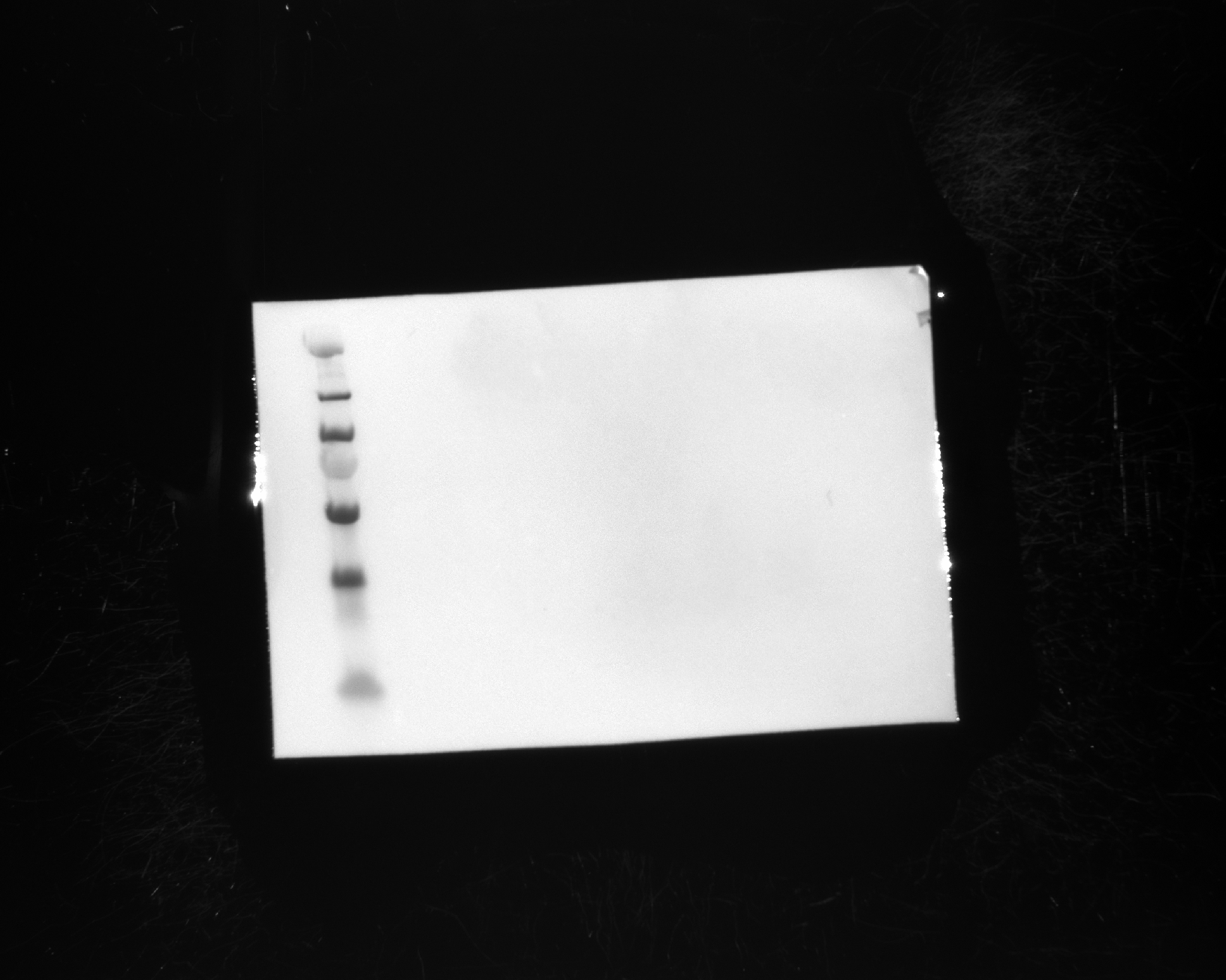

Supplement: Supplementary file 20 — Source Data for Expanded View [file EMBJ-42-e112100-s016.zip › FigureEV2/Source_Data_FigEV2C/Blot3/Blot3_anti-FLAG_ladder.tif]

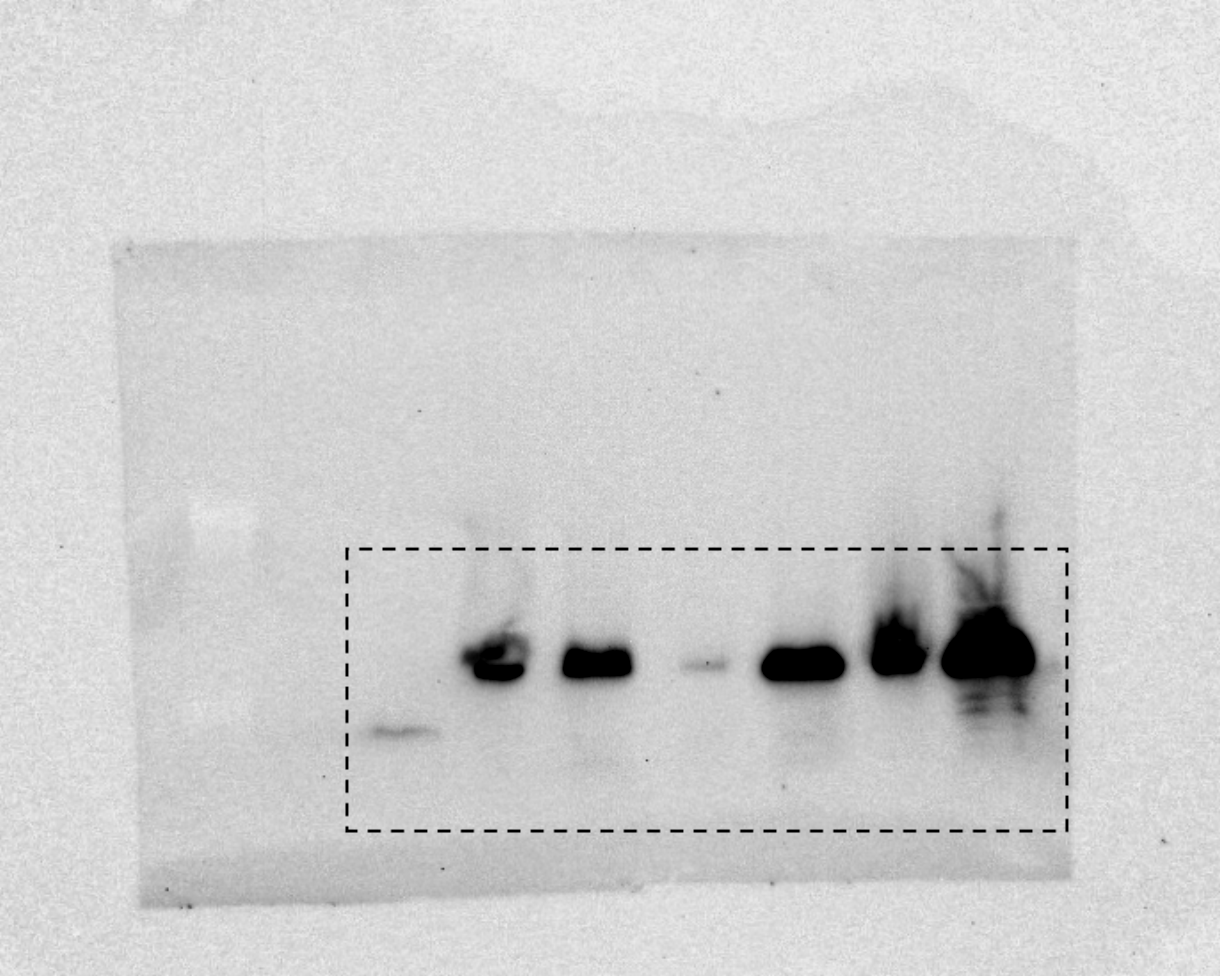

Supplement: Supplementary file 20 — Source Data for Expanded View [file EMBJ-42-e112100-s016.zip › FigureEV2/Source_Data_FigEV2C/Blot1/Blot1_anti_FLAG.tif]

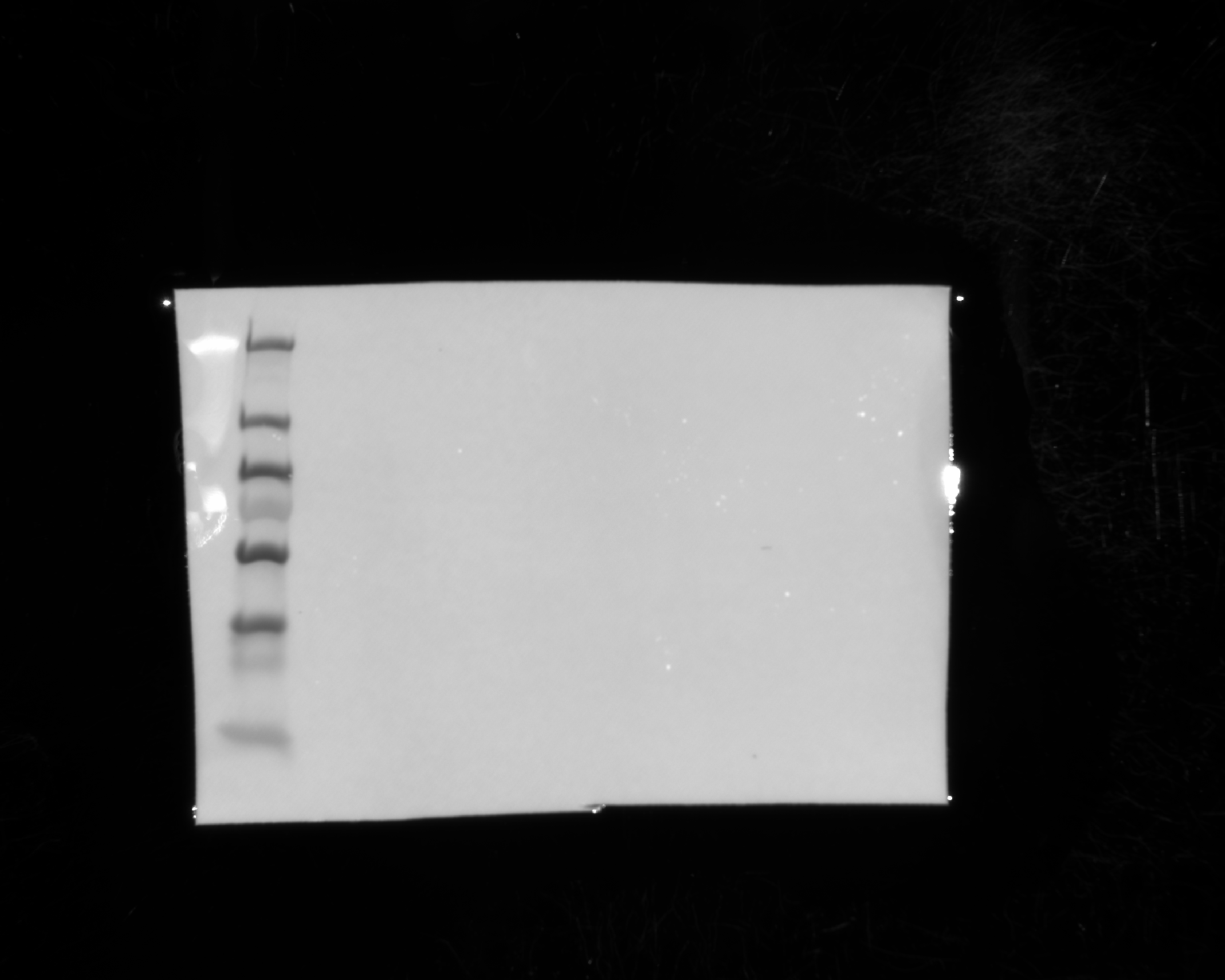

Supplement: Supplementary file 20 — Source Data for Expanded View [file EMBJ-42-e112100-s016.zip › FigureEV2/Source_Data_FigEV2C/Blot1/Blot1_anti-Tub_ladder.tif]

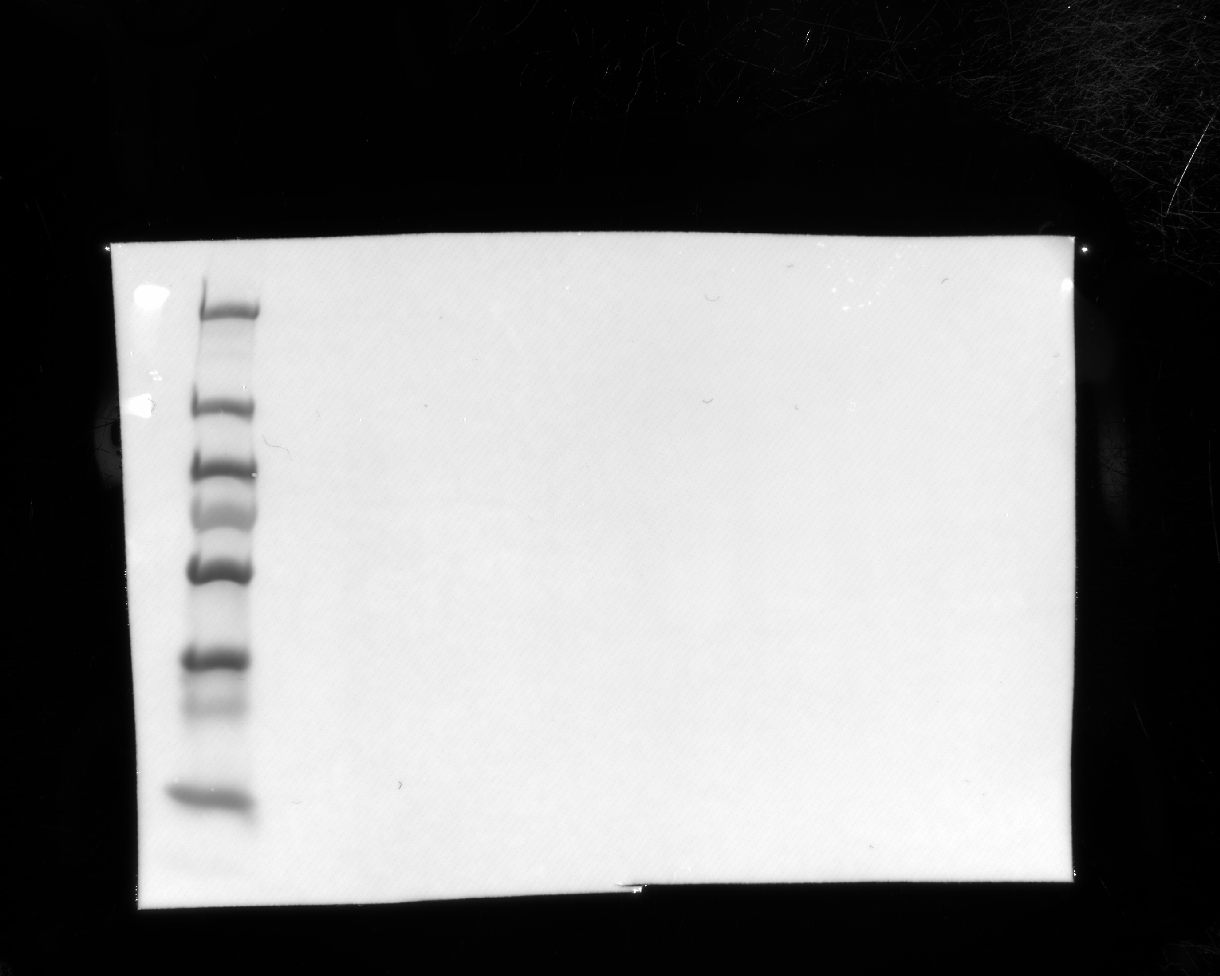

Supplement: Supplementary file 20 — Source Data for Expanded View [file EMBJ-42-e112100-s016.zip › FigureEV2/Source_Data_FigEV2C/Blot1/Blot1_anti_FLAG_ladder.tif]

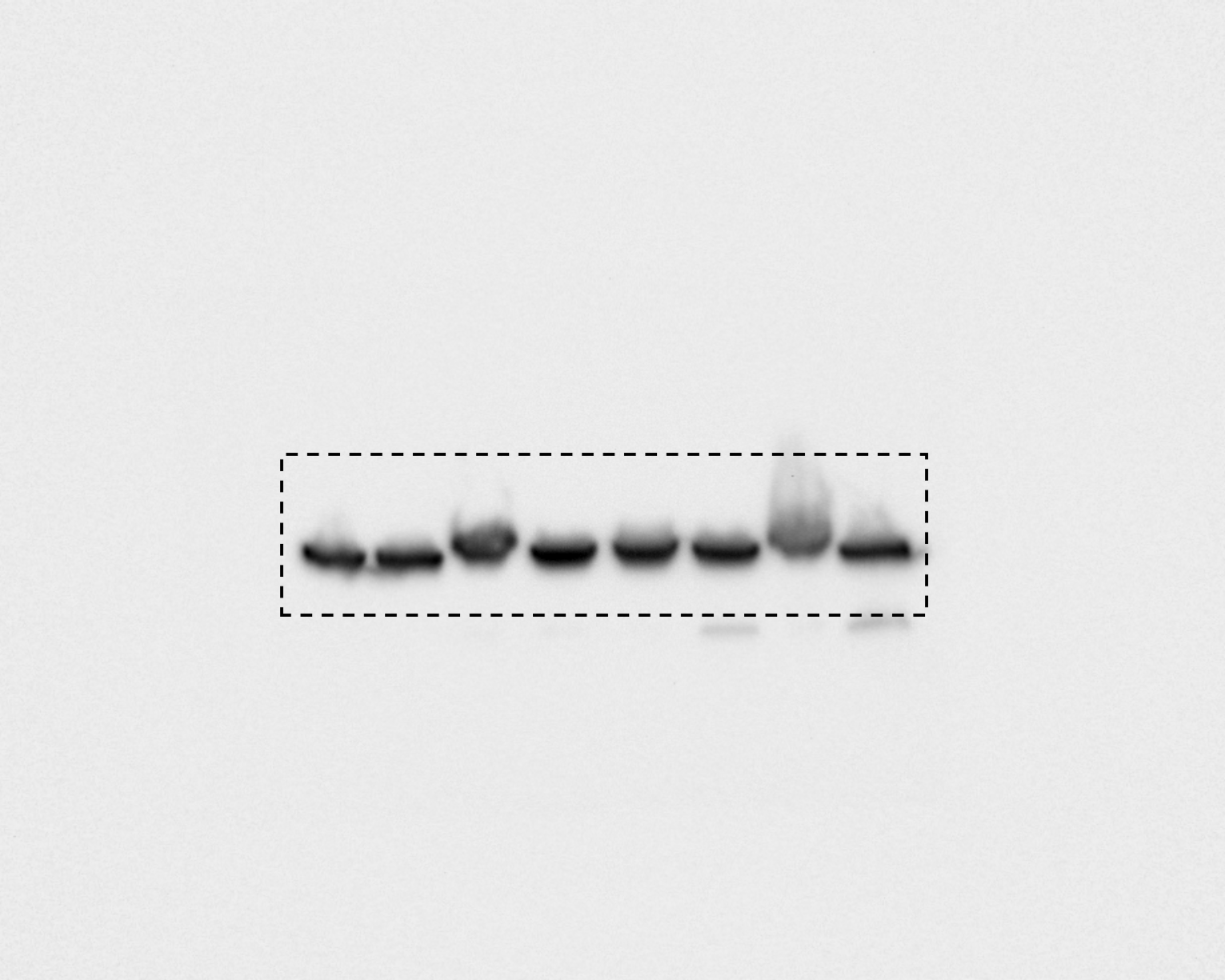

Supplement: Supplementary file 20 — Source Data for Expanded View [file EMBJ-42-e112100-s016.zip › FigureEV2/Source_Data_FigEV2C/Blot1/Blot1_anti-Tub.tif]
