# Supplementary material for: Systematic identification and characterization of repressive domains in Drosophila transcription factors
Source: EMBO J. 2022 Dec 22;42(3):e112100. doi: 10.15252/embj.2022112100 (PMC9890238; doi:10.15252/embj.2022112100)
Supplement: Supplementary file 25 [file EMBJ-42-e112100-s009.pdf]

THE EMBO JOURNAL

Systematic identification and characterization of repressive domains in Drosophila transcription factors

Loni Klaus, Bernardo de Almeida, Anna Vlasova, Filip Nemčko, Alexander Schleiffer, Katharina Bergauer, Lorena Hofbauer, Martina Rath, and Alexander Stark
DOI: 10.15252/embj.2022112100

Corresponding author(s): Alexander Stark (stark@starklab.org)

Review Timeline:

Submission Date:	13th Jul 22
Editorial Decision:	9th Aug 22
Revision Received:	17th Oct 22
Editorial Decision:	7th Nov 22
Revision Received:	21st Nov 22
Accepted:	1st Dec 22

Editor: Stefanie Boehm

Transaction Report:

(Note: With the exception of the correction of typographical or spelling errors that could be a source of ambiguity, letters and reports are not edited. Depending on transfer agreements, referee reports obtained elsewhere may or may not be included in this compilation. Referee reports are anonymous unless the Referee chooses to sign their reports.)

1st Editorial Decision 9th Aug 2022

Thank you again for submitting your manuscript reporting the systematic identification of transcriptional repressive domains to The EMBO Journal. We have now received two referee reports on your study, which are included below. In light of the referees' comments, we would like to invite you to prepare and submit a revised manuscript.

As you will see, the reviewers are overall positive and appreciate the findings and their interest to the field. Referee #1 nonetheless raises a number of points, that can however potentially be addressed by additional computational analyses and adding to the discussion. Please carefully consider these and all other referee comments and revise the manuscript and figures as needed, as well as providing a detailed response to each comment. Please also remember that the revised manuscript should then fulfill all EMBO Journal formatting requirements (please see below and: https://www.embopress.org/page/journal/14602075/authorguide#submissionofrevisions). We would also ask you to please upload it using the manuscript type "resource" (RES).

Referee #1:

This manuscript describes a systematic identification of linear (contiguous) transcriptional repression domains in Drosophila proteins. It is carefully written, easy to understand and provides a valuable resource for all molecular biologists, whether they are interested in Drosophila or human biology. It should be published, with just some minor additions or corrections:

1. Descriptions of the "dpse" and "ent1" promoters could not be found in the indicated publications under this name. Either provide a different reference, or a synonym, or describe these elements in the Methods section. Similarly, the TF database could not be accessed. Just add another supplemental table with the information that was drawn from that db: all transcription factors (including those that were not included in the current analysis), as well as their score.

2. The authors state that several of the identified RD-SLIMs "resemble" other motifs that were published in the literature or in databases. This statement is correct - at the same time, the newly found motifs are not identical to the published motifs. Therefore, the authors should spell out the "published motifs" (as drawn from the cited references) in the text so as to to let the readers compare them.

3. As to the conservation of the "repression motifs" in human repressors (Fig 4):
First, it is not clear to me how exactly the motifs were defined - e.g. did the authors simply search for "AAXXL", or did they use the PAMs from Fig 2C (as described in Table S6)?
Second, the experiment shown in Fig 4C was intended to demonstrate the evolutionary conservation (hence functional significance) of the "repression motifs", as compared to the conservation of the flanking sequences. As judged by the bar plots, 4 of the 5 motifs seem to be more conserved than the flanks - but the authors need to provide some statistical evaluation of this comparison (p-values).
Third, the authors argue that "AAxL" is not more conserved than its surrounding because the "repression motif" extends beyond "AAxL" into the surrounding. This argument is convincing, but the authors cannot simply stop there - instead, they have to repeat this analysis with an extended "repression motif" that comprises all of the conserved sequence. Along the same lines: the sequences flanking the other 4 motifs (grey boxes in Fig 4C) show quite different median conservation; e.g. the sequence surrounding "PLKKR" is more highly conserved than the "repression motif PxDLS", which itself is clearly more conserved than its surroundings. This suggests that some motifs extend beyond the definition that was used here, and that this analysis should also be repeated with an extended motif definition e.g. for "PLKKR".

4. Indicate the presence of all identified motifs within the RDs that were used as bait for the coIP analyses in Fig 3. For example, all baits used for Fig 3A contain the "PxDLS" motif, but according to Table S6 Tio-RD contains the "ent1" motif in addition. Considering that baits are vastly more abundant in the MS (e.g. in Fig 3A: 9 logs above CtBP), it is conceivable that a "secondary motif" in only one of the pooled baits might be responsible for recruiting the highlighted co-repressor (rather than the "primary motif" that is highlighted in the figure).

5. Fig 3A-D shows MS analyses of proteins that associate with GAL4-DBD-RD fusions. In each of these experiments, there are some significantly de-riched proteins (i.e. enriched in the GAL4-DBD control). Are any of these proteins transcription-associated factors, that might positively contribute to transcription in the absence of a fused RD domain and whose loss might explain part of the repressive activity of the RD? Do these de-riched proteins overlap between the different experiments?

6. Table S4 provides information on a "per protein" basis (i.e. it lists overlaps of the different proteins with various domains), but the main text speaks of "overlaps of RDs [i.e. individual domains] with other domains". The latter raises the possibiliy that two RD located within one protein may overlap different domains. The authors need to update Table S4 to contain "per RD" information.

7. The authors need to fully describe the statistical analysis they used for their various analyses. For example, a re-calculation of Table S3 gave 2 p-values slightly above 0.05 (for CHES-1-like-RD2 & ph-p; presumably because of assuming "2-tails"). This does by no means invalidate the authors' results, but reinforces the notion that parameters should be fully disclosed.

8. the observation that 53% of RD overlap with IDRs is interesting. However, to be able to judge the importance of such an observation the authors also have to provide the overall frequency of IDRs in Drosophila proteins.

9. p 6: "Two additional motifs were of low sequence complexity with multiple glutamate (motif 6) ..." This should read "glutamine" instead of "glutamate".

10. it is intriguing that (almost) all motif-mutant RDs are expressed at significantly higher levels than the corresponding wild type RDs (Fig S2C). Several of these motifs contain lysine residues, raising the possibility that motif ubiquitination might be involved in their destruction - and possibly also their activity (just a comment).

11. A more appropriate reference for the identification of Sin3A as a vertebrate corepressor, as well as the SID would be Ayer et al 1995, 1996.

Referee #2:

Transcriptional repressors are a key part of the gene regulatory infrastructure as evidenced by many examples where they contribute to correct gene expression programmes. However in general this class of proteins is poorly characterised in comparison to transcriptional activators. Here the authors have set out to systematically identify features of transcription factors that confer repressive properties, using a high throughput approach in Drosophila cells. The strategy builds nicely on previous ones from the Stark lab. In this case they screen for short proteins fragments that can repress expression from a GFP reporter. They start with a library in which the peptide motifs are fused to the GAL4 DNA binding domain and use reporter plasmids containing UAS sites adjacent to a constitutively active enhancer-promoter combination. Sequencing the plasmids enriched in the GFP negative cells identifies those containing repression motifs. In total they detect 195 repression domains and analyzing the sequences they identify common peptide motifs- 11 in total. They further investigate the partners for 5 of these, and show that each acts via a distinct corepressor.

As stated in the abstract: this work constitutes an invaluable resource and advances our understanding of repressors. For example they have identified novel peptide motifs conferring repression, as well as some that were previously known. The experiemtns that link some of those motifs to specific co-repressors are very nice and provide a valuable framework for future models of repressor function in different contexts. Its also notable that they can show conservation of some of these motifs including in human transcription factors

There are no major concerns. Altogether, it is a very nice and well executed study, all the data are of high quality and the authors have carried out a range of different experiments to substatiate their conclusions.

Minor concerns:
195 seems a relatively small number of repressors from the total screened. The authors mention possible reasons why not all repressor domains may be captured in their assay in the discussion but it was not clear whether they have screened for matches to the identified motifs outside those 195 proteins?

A thorough set of tables is provided- it would be helpful to have a summary list of these explaining what each is and a clear title for each.

Reviewer comments and answers

We would like to thank all reviewers for their comments. You can find our answers below each comment (text in blue).

Referee #1:
This manuscript describes a systematic identification of linear (contiguous) transcriptional repression domains in Drosophila proteins. It is carefully written, easy to understand and provides a valuable resource for all molecular biologists, whether they are interested in Drosophila or human biology. It should be published, with just some minor additions or corrections:

Thank you for the positive assessment of our manuscript. We address each of your comments below and made corresponding adjustments to the text, figures, and supplement.

1. Descriptions of the "dpse" and "ent1" promoters could not be found in the indicated publications under this name. Either provide a different reference, or a synonym, or describe these elements in the Methods section. Similarly, the TF database could not be accessed. Just add another supplemental table with the information that was drawn from that db: all transcription factors (including those that were not included in the current analysis), as well as their score.

Thank you for the heads up. We adjusted the methods section to better describe the promoter and enhancer elements we used. Moreover, we added more specific information to Table EV2, which also contains the whole plasmid sequences.

We apologize for the outdated link to the TF database. Apparently, the FlyTF database is currently being moved to another server (as indicated on this website: https://www.mrc-lmb.cam.ac.uk/genomes/FlyTF/). The FlyTF version we used, was downloaded on FlyTF.org on December 5, 2018. Following your suggestion, we now created a separate sheet in Table EV1 containing all 1168 factors, including the FlyTF scores.

2. The authors state that several of the identified RD-SLIMs "resemble" other motifs that were published in the literature or in databases. This statement is correct - at the same time, the newly found motifs are not identical to the published motifs. Therefore, the authors should spell out the "published motifs" (as drawn from the cited references) in the text to let the readers compare them.

This is a very good point, many thanks. We adjusted the respective paragraph in the results section to spell out motifs from the literature. In addition, we prepared Table EV7 which contains the consensus motifs of the MEME motifs we found, as well as regular expressions of known CoR-interacting SLiMs from the ELM database and additional literature examples. We also mention the MEME motif that resembles each of the “published motifs” for comparison.

3. As to the conservation of the "repression motifs" in human repressors (Fig 4): First, it is not clear to me how exactly the motifs were defined - e.g. did the authors simply search for "AAXXL", or did they use the PAMs from Fig 2C (as described in Table S6)?

Thank you for your comment. We indeed used the full PWMs from the initial MEME *de novo* discovery as input for FIMO searches among the human transcription-related proteins. We expanded the respective methods section to make this clearer.

Second, the experiment shown in Fig 4C was intended to demonstrate the evolutionary conservation (hence functional significance) of the "repression motifs", as compared to the conservation of the flanking sequences. As judged by the bar plots, 4 of the 5 motifs seem to be more conserved than the flanks - but the authors need to provide some statistical evaluation of this comparison (p-values).

Thank you for your comment. In the first version of the manuscript, we had used bar plots and color code to indicate statistical significance (FDR-corrected p-values) and are sorry that this had not been sufficiently clear. To improve clarity, we now indicate the P-values for the comparison of the conservation of motifs over their flanking sequences in the respective box plots (see Fig 4C,D).

Third, the authors argue that "AAxL" is not more conserved than its surrounding because the "repression motif" extends beyond "AAxL" into the surrounding. This argument is convincing, but the authors cannot simply stop there - instead, they have to repeat this analysis with an extended "repression motif" that comprises all of the conserved sequence. Along the same lines: the sequences flanking the other 4 motifs (grey boxes in Fig 4C) show quite different median conservation; e.g. the sequence surrounding "PLKKR" is more highly conserved than the "repression motif PxDLS", which itself is clearly more conserved than its surroundings. This suggests that some motifs extend beyond the definition that was used here, and that this analysis should also be repeated with an extended motif definition e.g. for "PLKKR".

This is a very interesting point, many thanks. To understand if some motifs extend beyond the lengths used here and to explore the other observations, we have now systematically analyzed the conservation of extended regions centered on each motif (+/-100 AA) and show the results Fig EV4C,D). This analysis shows that in contrast to the other 4 motifs, AAxxL is not more conserved than its surroundings (Fig EV4C), irrespective of the threshold used for motif matching (Fig EV4D). We now discuss this result and emphasize that it does not discount the functionality of the motif, which we validated experimentally, but rather suggests an increased sequence flexibility. Indeed, AAxxL-like Sin3A-interacting motifs in e.g. the fly TF Cabut or the human TF TIEG2 have AAEVAL or EAVEAL core sequences, respectively (Belacortu et al. 2012, PLoS one; Cook et al. 1999, J. Biol. Chem.), which both do not strictly follow the “AAxxL” pattern.

In addition, this new analysis sheds light on the higher conservation levels of the flanks around the HKKF and – to a lesser extent – PLKKR motifs. As the higher conservation has a broader shape and holds at longer distances from the motif core, it might result from overall more highly conserved proteins and/or protein domains/regions. We added these analyses to Fig EV4 and discuss them in the main text.

4. Indicate the presence of all identified motifs within the RDs that were used as bait for the coIP analyses in Fig 3. For example, all baits used for Fig 3A contain the "PxDLS" motif, but according to Table S6 Tio-RD contains the "ent1" motif in addition. Considering that baits are vastly more abundant in the MS (e.g. in Fig 3A: 9 logs above CtBP), it is conceivable that a "secondary motif" in only one of the pooled baits might be responsible for recruiting the highlighted co-repressor (rather than the "primary motif" that is highlighted in the figure).

Thank you for your comment. Indeed, some of the baits that we used for the IP-MS contain a second motif, however these were only found with lenient cutoffs (Table EV6 provides the FIMO results for both, a stringent and – for comprehensiveness – a lenient cutoff) and don’t fit well to the consensus core motifs. Therefore, and because several of the bait RDs lose their repressive activity upon mutation of the primary motif (for example PLKKR in Kr-h1-RD2, see Figure 2I), we don’t think that the secondary, sub-threshold motifs in one of four RDs per pool contributes to the repressive activity

or CoR interaction. However, we agree that this is an important point and now discuss our reasoning in the respective results paragraph and provide a new sheet in Table EV9 which lists the RDs of the different pools and the secondary motifs which some of them contain including the matched motif sequences. Many thanks.

5. Fig 3A-D shows MS analyses of proteins that associate with GAL4-DBD-RD fusions. In each of these experiments, there are some significantly de-riched proteins (i.e. enriched in the GAL4-DBD control). Are any of these proteins transcription-associated factors, that might positively contribute to transcription in the absence of a fused RD domain and whose loss might explain part of the repressive activity of the RD? Do these de-riched proteins overlap between the different experiments?

Thank you for your comment, that's indeed an intriguing idea. However, the top hits seem to be unspecific binders unrelated to transcriptional regulation, including lysosomal proteins (e.g. CP1 Cysteine proteinase-1). Only among the weakly de-enriched proteins are a few transcription-related factors, both activators and repressors, but these don't overlap between the different experiments. We now added this notion to the figure legend.

6. Table S4 provides information on a "per protein" basis (i.e. it lists overlaps of the different proteins with various domains), but the main text speaks of "overlaps of RDs [i.e. individual domains] with other domains". The latter raises the possibility that two RD located within one protein may overlap different domains. The authors need to update Table S4 to contain "per RD" information.

Thank you for pointing this out. The table (Table EV5) now contains an additional column with the identity of the RD which overlaps with the known domain, to allow a clear distinction between multiple RDs in the same protein.

7. The authors need to fully describe the statistical analysis they used for their various analyses. For example, a re-calculation of Table S3 gave 2 p-values slightly above 0.05 (for CHES-1-like-RD2 & ph-p; presumably because of assuming "2-tails"). This does by no means invalidate the authors' results, but reinforces the notion that parameters should be fully disclosed.

Thank you for pointing out that we had not explained the statistical analyses sufficiently well. For the main validation set we used paired, two-tailed student T-tests comparing the log2 median GFP signals in the control versus the RD condition for three replicates. For the mutagenesis experiments and the RNAi experiments we applied the same statistical test but compared the log2 FC repression values between wild type and mutant, or between noRNA versus dsRNA treatment, respectively. We adjusted the respective method sections to clarify the statistical tests and reviewed all values in the respective extended view tables (fixing a copy-and-paste mistake) and updated the respective figure panels. Many thanks!

8. the observation that 53% of RD overlap with IDRs is interesting. However, to be able to judge the importance of such an observation the authors also have to provide the overall frequency of IDRs in Drosophila proteins.

This is a good point! We have now checked the prevalence of IDRs among 50 amino acid fragments (same size as the RDs) from *Dmel* transcription-related proteins or all *Dmel* proteins (excluding sequences that overlap RDs). Among the transcription-related proteins 36% of these 50 amino acid fragments overlap IDRs. For all *Dmel* proteins the number is 28%. We now add these comparisons

and discuss the fact that IDRs are more prevalent among RDs (53%) than other proteins, many thanks.

9. p 6: "Two additional motifs were of low sequence complexity with multiple glutamate (motif 6) ..." This should read "glutamine" instead of "glutamate".

Thank you for pointing out this typo. We changed it to the correct amino acid.

10. it is intriguing that (almost) all motif-mutant RDs are expressed at significantly higher levels than the corresponding wild type RDs (Fig S2C). Several of these motifs contain lysine residues, raising the possibility that motif ubiquitination might be involved in their destruction - and possibly also their activity (just a comment).

Yes, this is indeed interesting! While we have not seen a link between ubiquitination and repression in the literature, a previous study reports that some transcription activating domains overlap with degrons, which might present a way by which such effector domains can be regulated (Salghetti et al. 2000, PNAS; Geng et al. 2012, Annu Rev Biochem.). This might be an interesting commonality between activating and repressive domains!

11. A more appropriate reference for the identification of Sin3A as a vertebrate corepressor, as well as the SID would be Ayer et al 1995, 1996.

Thank you for this tip! We added this reference to the respective paragraphs.

Referee #2:

Transcriptional repressors are a key part of the gene regulatory infrastructure as evidenced by many examples where they contribute to correct gene expression programs. However, in general this class of proteins is poorly characterized in comparison to transcriptional activators. Here the authors have set out to systematically identify features of transcription factors that confer repressive properties, using a high throughput approach in Drosophila cells. The strategy builds nicely on previous ones from the Stark lab. In this case they screen for short proteins fragments that can repress expression from a GFP reporter. They start with a library in which the peptide motifs are fused to the GAL4 DNA binding domain and use reporter plasmids containing UAS sites adjacent to a constitutively active enhancer-promoter combination. Sequencing the plasmids enriched in the GFP negative cells identifies those containing repression motifs. In total they detect 195 repression domains and analyzing the sequences they identify common peptide motifs- 11 in total. They further investigate the partners for 5 of these and show that each acts via a distinct corepressor.

As stated in the abstract: this work constitutes an invaluable resource and advances our understanding of repressors. For example, they have identified novel peptide motifs conferring repression, as well as some that were previously known. The experiments that link some of those motifs to specific co-repressors are very nice and provide a valuable framework for future models of repressor function in different contexts. Its also notable that they can show conservation of some of these motifs including in human transcription factors.

There are no major concerns. Altogether, it is a very nice and well executed study, all the data are of high quality and the authors have carried out a range of different experiments to substantiate their conclusions.

Thank you for the very positive assessment of our manuscript. We address each of your comments below and made adjustment in the text, figures, and supplements.

Minor concerns:

1) 195 seems a relatively small number of repressors from the total screened. The authors mention possible reasons why not all repressor domains may be captured in their assay in the discussion but it was not clear whether they have screened for matches to the identified motifs outside those 195 proteins?

Thank you for pointing out that we had not explained sufficiently clearly that we indeed screened all indicated ORFs, including regions that matched to motifs outside the 195 proteins. Inspired by this comment, we determined matches of the 5 main motifs (EH1, AAxxL, PxDLS, PLKKR, HKKF) within non-RD fragments. Interestingly, these fragments had only background RD-seq signals, suggesting that they were indeed inactive rather than missed by our stringent cutoff (Fig EV5A). We therefore compared the sequences of functional motifs within RDs and non-functional motifs (Fig EV5B-F), which revealed that functional motifs fit better to the consensus motifs (as in Fig 2C) and that their flanking sequences were enriched for certain types of amino acids (e.g. serine residues in the case of EH1 motifs). We now added both analyses as a new supplementary figure (Fig EV5) and discuss them in the main text. Many thanks.

2) A thorough set of tables is provided- it would be helpful to have a summary list of these explaining what each is and a clear title for each.

This is a very good idea. We now include a summary list as Table EV19.

Thank you again for submitting your revised manuscript. We have now received comments from referee #1 (please see below) and I am happy to say they now support publication. Therefore I would ask you to please resolve a number of editorial issues that are listed in detail below. Please use the document that the data editors have added their comments to for any changes (see below). If you have any further questions regarding the specific points listed below or the final manuscript version, please feel free to contact me. Once these final issues are resolved we will be happy to formally accept the manuscript for publication.

Referee #1:

The authors have diligently addressed the (many, but minor) comments. I strongly suggest publication of this work in the present form!

All editorial and formatting issues were resolved by the authors.

2nd Revision - Editorial Decision 1st Dec 2022

Thank you for submitting the final revised version of your manuscript and addressing the remaining points. I am happy to inform you that we have formally accepted your study for publication in The EMBO Journal.

EMBO Press Author Checklist

Corresponding Author Name: Alexander Stark
Journal Submitted to: EMBO Journal
Manuscript Number: EMBOJ-2022-112100R

USEFUL LINKS FOR COMPLETING THIS FORM
The EMBO Journal - Author Guidelines
EMBO Reports - Author Guidelines
Molecular Systems Biology - Author Guidelines
EMBO Molecular Medicine - Author Guidelines

Reporting Checklist for Life Science Articles (updated January
This checklist is adapted from Materials Design Analysis Reporting (MDAR) Checklist for Authors. MDAR establishes a minimum set of requirements in transparent reporting in the life sciences (see Statement of Task: 10.31222/osf.io/9sm4x). Please follow the journal's guidelines in preparing your
Please note that a copy of this checklist will be published alongside your article.

Abridged guidelines for figures
1. Data
The data shown in figures should satisfy the following conditions:
- the data were obtained and processed according to the field's best practice and are presented to reflect the results of the experiments in an accurate and unbiased manner.
- ideally, figure panels should include only measurements that are directly comparable to each other and obtained with the same assay.
- plots include clearly labeled error bars for independent experiments and sample sizes. Unless justified, error bars should not be shown for technical
- if n<5, the individual data points from each experiment should be plotted. Any statistical test employed should be justified.
- Source Data should be included to report the data underlying figures according to the guidelines set out in the authorship guidelines on Data

2. Captions
Each figure caption should contain the following information, for each panel where they are relevant:
- a specification of the experimental system investigated (eg cell line, species name).
- the assay(s) and method(s) used to carry out the reported observations and measurements.
- an explicit mention of the biological and chemical entity(ies) that are being measured.
- an explicit mention of the biological and chemical entity(ies) that are altered/varied/perturbed in a controlled manner.
- the exact sample size (n) for each experimental group/condition, given as a number, not a range;
- a description of the sample collection allowing the reader to understand whether the samples represent technical or biological replicates (including how many animals, litters, cultures, etc.).
- a statement of how many times the experiment shown was independently replicated in the laboratory.
- definitions of statistical methods and measures:
  - common tests, such as t-test (please specify whether paired vs. unpaired), simple χ2 tests, Wilcoxon and Mann-Whitney tests, can be unambiguously identified by name only, but more complex techniques should be described in the methods section;
  - are tests one-sided or two-sided?
  - are there adjustments for multiple comparisons?
  - exact statistical test results, e.g., P values = x but not P values < x;
  - definition of 'center values' as median or average;
  - definition of error bars as s.d. or s.e.m.

Please complete ALL of the questions below.
Select "Not Applicable" only when the requested information is not relevant for your study.

Materials

Newly Created Materials	Information included in the manuscript?	In which section is the information available? (Reagents and Tools Table, Materials and Methods, Figures, Data Availability Section)
New materials and reagents need to be available; do any restrictions apply?	Yes	Reagents and Tools Table, Materials and Methods

Antibodies	Information included in the manuscript?	In which section is the information available? (Reagents and Tools Table, Materials and Methods, Figures, Data Availability Section)
For **antibodies** provide the following information:<br>- Commercial antibodies: RRID (if possible) or supplier name, catalogue number and or/clone number<br>- Non-commercial: RRID or citation	Yes	Reagents and Tools Table, Materials and Methods

DNA and RNA sequences	Information included in the manuscript?	In which section is the information available? (Reagents and Tools Table, Materials and Methods, Figures, Data Availability Section)
**Short novel DNA or RNA including primers, probes**: provide the sequences.	Yes	Materials and Methods, Table EV12

Cell materials	Information included in the manuscript?	In which section is the information available? (Reagents and Tools Table, Materials and Methods, Figures, Data Availability Section)
**Cell lines:** Provide species information, strain. Provide accession number in repository **OR** supplier name, catalog number, clone number, and/**OR** RRID.	Yes	Reagents and Tool, Materials and Methods
**Primary cultures:** Provide species, strain, sex of origin, genetic modification status.	Not Applicable	
Report if the cell lines were recently **authenticated** (e.g., by STR profiling) and tested for mycoplasma contamination.	Yes	Reagents and Tool, Materials and Methods

Experimental animals	Information included in the manuscript?	In which section is the information available? (Reagents and Tools Table, Materials and Methods, Figures, Data Availability Section)
**Laboratory animals or Model organisms:** Provide species, strain, sex, age, genetic modification status. Provide accession number in repository **OR** supplier name, catalog number, clone number, **OR** RRID.	Not Applicable	
**Animal observed in or captured from the field:** Provide species, sex, and age where possible.	Not Applicable	
Please detail **housing and husbandry conditions**.	Not Applicable	

Plants and microbes	Information included in the manuscript?	In which section is the information available? (Reagents and Tools Table, Materials and Methods, Figures, Data Availability Section)
**Plants:** provide species and strain, ecotype and cultivar where relevant, unique accession number if available, and source (including location for collected wild specimens).	Not Applicable	
**Microbes:** provide species and strain, unique accession number if available, and source.	Yes	Reagents and Tools Table, Material and Methods

Human research participants	Information included in the manuscript?	In which section is the information available? (Reagents and Tools Table, Materials and Methods, Figures, Data Availability Section)
If collected and within the bounds of privacy constraints report on age, sex and gender or ethnicity for all study participants.	Not Applicable	

Core facilities	Information included in the manuscript?	In which section is the information available? (Reagents and Tools Table, Materials and Methods, Figures, Data Availability Section)
If your work benefited from core facilities, was their service mentioned in the acknowledgments section?	Yes	Acknowledgments

Design

Study protocol	Information included in the manuscript?	In which section is the information available? (Reagents and Tools Table, Materials and Methods, Figures, Data Availability Section)
If study protocol has been **pre-registered, provide DOI in the manuscript**. For clinical trials, provide the trial registration number **OR** cite DOI.	Not Applicable	
Report the **clinical trial registration number** (at ClinicalTrials.gov or equivalent), where applicable.	Not Applicable	

Laboratory protocol	Information included in the manuscript?	In which section is the information available? (Reagents and Tools Table, Materials and Methods, Figures, Data Availability Section)
Provide DOI OR other citation details if **external detailed step-by-step protocols** are available.	Not Applicable	

Experimental study design and statistics	Information included in the manuscript?	In which section is the information available? (Reagents and Tools Table, Materials and Methods, Figures, Data Availability Section)
Include a statement about **sample size** estimate even if no statistical methods were used.	Yes	Materials and Methods
Were any steps taken to minimize the effects of subjective bias when allocating animals/samples to treatment (e.g. **randomization procedure**)? If yes, have they been described?	Not Applicable	
Include a statement about **blinding** even if no blinding was done.	Not Applicable	
Describe **inclusion/exclusion criteria** if samples or animals were excluded from the analysis. Were the criteria pre-established?<br><br>If sample or data points were omitted from analysis, report if this was due to attrition or intentional exclusion and provide justification.	Not Applicable	
For every figure, are **statistical tests** justified as appropriate? Do the data meet the assumptions of the tests (e.g., normal distribution)? Describe any methods used to assess it. Is there an estimate of variation within each group of data? Is the variance similar between the groups that are being statistically compared?	Yes	Materials and Methods, Figure Legends

Sample definition and in-laboratory replication	Information included in the manuscript?	In which section is the information available? (Reagents and Tools Table, Materials and Methods, Figures, Data Availability Section)
In the figure legends: state number of times the experiment was **replicated** in laboratory.	Yes	Figure Legends
In the figure legends: define whether data describe **technical or biological replicates**.	Yes	Figure Legends

Ethics

Ethics	Information included in the manuscript?	In which section is the information available? (Reagents and Tools Table, Materials and Methods, Figures, Data Availability Section)
Studies involving **human participants**: State details of **authority granting ethics approval** (IRB or equivalent committee(s), provide reference number for approval.	Not Applicable	
Studies involving **human participants**: Include a statement confirming that **informed consent** was obtained from all subjects and that the experiments conformed to the principles set out in the WMA Declaration of Helsinki and the Department of Health and Human Services Belmont Report.	Not Applicable	
Studies involving **human participants:** For publication of **patient photos**, include a statement confirming that consent to publish was obtained.	Not Applicable	
Studies involving experimental **animals**: State details of **authority granting ethics approva**l (IRB or equivalent committee(s), provide reference number for approval. Include a statement of compliance with ethical regulations.	Not Applicable	
Studies involving **specimen and field samples:** State if relevant **permits** obtained, provide details of authority approving study; if none were required, explain why.	Not Applicable	

Dual Use Research of Concern (DURC)	Information included in the manuscript?	In which section is the information available? (Reagents and Tools Table, Materials and Methods, Figures, Data Availability Section)
Could your study fall under dual use research restrictions? Please check biosecurity documents and list of **select agents and toxins** (CDC): https://www.selectagents.gov/sat/list.htm	Not Applicable	
If you used a select agent, is the security level of the lab appropriate and reported in the manuscript?	Not Applicable	
If a study is subject to dual use research of concern regulations, is the name of the **authority granting approval and reference number** for the regulatory approval provided in the manuscript?	Not Applicable	

Reporting

The MDAR framework recommends adoption of discipline-specific guidelines, established and endorsed through community initiatives. Journals have their own policy about requiring specific guidelines and recommendations to complement MDAR.

Adherence to community standards	Information included in the manuscript?	In which section is the information available? (Reagents and Tools Table, Materials and Methods, Figures, Data Availability Section)
State if relevant guidelines or checklists (e.g., **ICMJE, MIBBI, ARRIVE, PRISMA**) have been followed or provided.	Not Applicable	
For **tumor marker prognostic studies,** we recommend that you follow the **REMARK** reporting guidelines (see link list at top right). See author guidelines, under 'Reporting Guidelines'. Please confirm you have followed these guidelines.	Not Applicable	
For **phase II and III randomized controlled trials**, please refer to the **CONSORT** flow diagram (see link list at top right) and submit the CONSORT checklist (see link list at top right) with your submission. See author guidelines, under 'Reporting Guidelines'. Please confirm you have submitted this list.	Not Applicable	

Data Availability

Data availability	Information included in the manuscript?	In which section is the information available? (Reagents and Tools Table, Materials and Methods, Figures, Data Availability Section)
Have **primary datasets** been deposited according to the journal's guidelines (see 'Data Deposition' section) and the respective accession numbers provided in the Data Availability Section?	Yes	Data Availability Section
Were **human clinical and genomic datasets** deposited in a public access-controlled repository in accordance to ethical obligations to the patients and to the applicable consent agreement?	Not Applicable	
Are **computational models** that are central and integral to a study available without restrictions in a machine-readable form? Were the relevant accession numbers or links provided?	Not Applicable	
If publicly available data were reused, provide the respective **data citations in the reference list**.	Not Applicable	
